# Social support, educational, and behavioral modification interventions for improving household disaster preparedness in the general community-dwelling population: a systematic review and meta-analysis

**DOI:** 10.3389/fpubh.2023.1257714

**Published:** 2024-02-23

**Authors:** Taryn Amberson, Tara Heagele, Tamar Wyte-Lake, Mary Pat Couig, Sue Anne Bell, Manoj J Mammen, Valerie Wells, Jessica Castner

**Affiliations:** ^1^Health Systems and Population Health, University of Washington, Seattle, WA, United States; ^2^Hunter-Bellevue School of Nursing, Hunter College, The City University of New York, New York City, NY, United States; ^3^Veterans Emergency Management Evaluation Center, Los Angeles, CA, United States; ^4^Department of Family Medicine, Oregon Health and Science University, Portland, OR, United States; ^5^College of Nursing, University of New Mexico, Albuquerque, NM, United States; ^6^University of Michigan, Ann Arbor, MI, United States; ^7^University of Rochester, Rochester, NY, United States; ^8^MRC/CSO Social and Public Health Sciences Unit, University of Glasgow, Glasgow, United Kingdom; ^9^Castner Incorporated, Grand Island, NY, United States; ^10^University at Albany School of Public Health, Albany, NY, United States

**Keywords:** systematic review, meta-analysis, public health, disaster preparedness, household preparedness, emergency preparedness, disaster planning, disasters

## Abstract

**Background:**

The efficacy of household emergency preparedness interventions for community-dwelling, non-institutionalized people is largely unknown.

**Objective:**

To ascertain the state of the science on social support, educational, and behavioral modification interventions to improve all-hazard household disaster preparedness.

**Design:**

Systematic review and meta-analysis.

**Methods:**

Databases, trial registers, reports, and websites were searched, and citation trails followed utilizing replicable methods. Individual, cluster, and cross-over randomized controlled trials of non-institutionalized, community-dwelling populations and non-randomized controlled trials, controlled before-after, and program evaluation studies were included. At least two review authors independently screened each potentially relevant study for inclusion, extracted data, and assessed the risk of bias. Risk of bias was assessed using Cochrane’s RoB2 tool for randomized studies and ROBINS-I tool for nonrandomized studies. Meta-analyses were applied using a random-effects model. Where meta-analysis was not indicated, results were synthesized using summary statistics of intervention effect estimates and vote counting based on effect direction. The evidence was rated using GRADE.

**Results:**

17 studies were included with substantial methodological and clinical diversity. No intervention effect was observed for preparedness supplies (OR = 6.12, 95% 0.13 to 284.37) or knowledge (SMD = 0.96, 95% CI −0.15 to 2.08) outcomes. A small positive effect (SMD = 0.53, 95% CI 0.16 to 0.91) was observed for preparedness behaviors, with very low certainty of evidence. No studies reported adverse effects from the interventions.

**Conclusion:**

Research designs elucidating the efficacy of practical yet complex and multi- faceted social support, educational, and behavioral modification interventions present substantial methodological challenges where rigorous study design elements may not match the contextual public health priority needs and resources where interventions were delivered. While the overall strength of the evidence was evaluated as low to very low, we acknowledge the valuable and informative work of the included studies. The research represents the seminal work in this field and provides an important foundation for the state of the science of household emergency preparedness intervention effectiveness and efficacy. The findings are relevant to disaster preparedness practice and research, and we encourage researchers to continue this line of research, using these studies and this review to inform ongoing improvements in study designs.

## 1 Introduction

Around the globe, disasters have had and will continue to have an impact on individuals, households, communities, states, regions, and nations. In October 2020, the Center for Research on the Epidemiology of Disasters (CRED) and the United Nations Office for Disaster Risk Reduction (UNDRR) released a report on the toll of disasters. This report concluded that during the time from 1999 to 2019, 7,348 disaster events were recorded worldwide, claiming approximately 1.23 million lives, for an average of 60,000 *per annum*, and affected a total of over 4 billion people. Additionally, disasters led to approximately USD 2.97 trillion in economic losses worldwide (1). Furthermore, the COVID-19 pandemic demonstrated that with globalization, people may be indirectly affected by product supply chain disruption for supplies necessary to maintain life during home quarantine or isolation (2). Our systematic review synthesizes existing research on household emergency preparedness interventions and fills a current gap in the literature. Adjacent topics in currently published literature include the following: a literature review on how social capital can be used to foster household emergency preparedness (3); a literature review on the effectiveness of various preparedness educational activities targeted at health professionals (4); a literature review on the social and physical determinants of disaster-related morbidity and mortality of older adult and medically frail community members (5); a scoping review on how community-based service providers can foster household emergency preparedness for community-dwelling clients (6); a literature review on how home health agencies can improve the disaster preparedness of patients and providers (7); an integrative review describing knowledge and skills that healthcare providers need to provide appropriate care for older adult community members during disaster response efforts (8); a methodological review on how practitioners evaluate the effectiveness of disaster education programs targeted to children (9), and; a systematic review of post-disaster chronic disease outcomes for older adults (10).

This systematic review of household emergency preparedness interventions for community-dwelling non-institutionalized people of the general population is the first such review to the authors’ knowledge. Research on the effectiveness of household emergency preparedness interventions is greatly needed. This review will assist public health, emergency management, and healthcare professionals with evidence-based decisions on specific interventions to be implemented in their respective communities and enable researchers to ascertain gaps and strengths in the existing evidence. This review provides evidence-based recommendations to guide policymakers across multiple disciplines to support all-hazard preparedness decision-making. It also assists other stakeholders (public and private) in prioritizing how to best invest in disaster preparedness efforts to enhance effective community response while minimizing loss of life.

This review synthesized available evidence on the effects of social, educational, and behavioral modification interventions to improve all-hazard household disaster preparedness in residential settings. For conciseness, “all-hazard household disaster preparedness” will be referred to as “household preparedness” and “all-hazard household disaster preparedness behaviors” as “preparedness behaviors.” Household preparedness is defined as evidence of individual household supplies, knowledge, and established communication plans to shelter-in-place, evacuate, and locate other loved ones (or social support persons) who do not reside in the same household. Household preparedness information disseminated through public health and clinician education, social networks, and behavioral modification interventions is widely assumed and delivered as best-evidence practice (3, 11, 12).

Disaster is defined as “a situation or event that overwhelms local capacity, necessitating a request at the national or international level for external assistance; an unforeseen and often sudden event that causes great damage, destruction and human suffering” (13, 14). When operationalizing this definition for the International Disaster Database (EM-DAT), CRED requires one or more of the inclusion criteria be present: 10 or more people reported killed, 100 or more people reported affected, declaration of a state of emergency, and/or a call for international assistance (14). Disasters are classified as natural or technological, emphasizing that human causes are linked to both disaster groups, and that human agency can prevent and mitigate their impact (14). In this EM-DAT classification system, natural disasters are subdivided into subgroups of geophysical, meteorological, hydrological, climatological, biological, and extraterrestrial. Technological disasters are subdivided into subgroups of industrial, transport, and miscellaneous (collapse, explosion, fire, other) accidents.

It is important to clarify the distinction between disaster and hazard. Hazards are defined by CRED as extreme or severe events (earthquakes, floods, heat waves, etc.) that naturally occur all over the world (14). These hazards are considered disasters only when they affect a vulnerable human settlement and lives are lost or livelihoods affected (15). In this review, the term “natural disaster” denotes a natural hazard that has affected a human settlement that was not appropriately organized or resourced to withstand the hazard’s impact. This highlights the potential power of disaster risk governance to effectively reduce and manage disaster risk (15). The type of hazard exposure is not the focus of our review. Rather, we focus on disaster preparedness at the household level for any or all hazards.

A more prepared public leads to more resilient communities and therefore more effective recovery and response in the post-disaster period (16). Disaster preparedness research over the past 20 years reveals that some households have made efforts to be prepared. However, many studies have found that households remain unprepared for disasters, even in disaster-prone areas (17). Research from Asia (18, 19), North America (20), New Zealand (21), Australia (22), and Africa (23), as well as from the Middle East (24, 25), has revealed low levels of household preparedness (26). In the United States, surveys at both national and local levels consistently demonstrate that the public remains under-prepared (27–30). Another study involving 3,541 households from four regions in China found poor household preparedness levels (31).

To improve preparedness behaviors, interventionists working to effect behavior change would first need to understand the barriers and motivators associated with adopting said behavior (32). Considerations should be made to engage individuals at varying levels of awareness, motivation, and preparedness. Therefore, interventions must target or tailor messages to specific population groups, as well as to those at different stages of preparedness (33). Thus, household preparedness interventions may be tailored to one or more of the most common or most threatening disaster hazards in the study setting, and to the unique vulnerabilities applicable to the population included in the study. Multi-component interventions with social support, educational, and behavioral modification intervention components as described below are well poised to develop a tailored approach to the different stages of preparedness when developing translatable and adaptable preparedness behaviors. Ultimately, healthcare utilization, mortality, and post-disaster functioning may be improved for members within a household who are better prepared for disasters.

Our PICO research question was as follows: in the general, non-institutionalized, community-dwelling population (P), do social support, educational, and behavioral modification interventions (I) compared to no or non-interaction interventions, including usual mass public service messaging (C), improve household preparedness behaviors, supplies, and/or knowledge (O)? We also sought to assess whether these interventions have effects on healthcare utilization, mortality, and mental health or physical functioning post disaster.

## 2 Materials and methods

Household preparedness social support, educational, and behavioral modification interventions are developed and implemented to improve knowledge, motivation, and resources and are expected to translate into concrete preparedness behaviors.

### 2.1 Social support interventions

Social support interventions include the provision of philanthropic, or public, social services and peer support. Social interventions are particularly relevant for households with economic vulnerability or independent functioning that may not have the resources to affect the desired outcome otherwise. Social support interventions often mitigate the inability to achieve household preparedness without material support or human networked co-functioning, particularly in circumstances where individual or collective household knowledge or motivation alone is insufficient to achieve the intended outcome. Social support interventions are emotional and financial and involve resource-sharing, peer-training, social network information dissemination, and companionship offered among family, friends, peers, faith-based or service communities (including non-governmental aid organizations), or neighbors. Social support may include social service agency interventions for subsidized housing, materials, and supplies, or structured support groups. Outside of train-the-trainer models, social support typically does not include training, professional counseling, or educational interventions consisting of professional, paid services from a public health worker, a health educator, or a clinician. However, paraprofessionals provide social support as structured components of community health worker networks or successful referrals to social service agencies. Referrals for disaster registries, transportation, or other disaster resources are considered social support interventions.

Lack of social support is a major risk factor for poor household preparedness and worsened post-disaster mental and physical health outcomes (34, 35). Social support improves self-management and self-reliance for people with complex chronic diseases such as diabetes (36, 37). Incorporating small group discussions and social support has led to greater improvement in household preparedness education interventions compared to population-level media education alone (38). Social connections are a key aspect of rural older adult household disaster preparedness (39). Direct provision of disaster supplies can improve longitudinal household preparedness as seen in families of children with special needs (40). Well-established social networks and community social support services enhance disaster preparedness and resilience after disasters (3, 41).

### 2.2 Educational interventions

Educational interventions may include systematic instruction, structured information-sharing, or professional provision of self-care information and information resources. Household preparedness educational interventions can take place in clinical and community education settings and can be provided as take-home reading materials and Internet-available or pre-prepared video/audio instruction.

Structured educational interventions on household preparedness include information on supplies and/or evacuation, sheltering-in-place, and communication planning (42), as well as drills and exercises to practice household disaster response (31). Education may be tailored to address one or more of the most prevalent disaster hazard vulnerabilities of the region in which the study takes place. Education may also be tailored to the specific needs of a population with a chronic disease or disability. Household preparedness interventions have been tested in households (42), in community education (38, 43), as part of virtual reality (44), and in clinical settings (40), and have been explored for use in pregnant women (43), as well as in families with special needs children (40, 45), members of Hispanic/Latine communities (38), and general community members (31, 42).

As defined by Wakefield (46), mass media campaigns utilize existing media channels such as mail, Internet, radio, and television to expose large numbers of people to messages that encourage behavior change. Population-based household preparedness education tends to involve a comprehensive approach, with additional optional materials tailored to specific vulnerabilities. For the purposes of this review, we did not consider population-level mass media campaigns as educational interventions. Rather, they served as controls.

### 2.3 Behavioral modification interventions

Behavioral modification interventions are intended to change human behavior patterns through motivational techniques, often with positive and negative reinforcement. These interventions include motivational interviewing, cognitive or behavior therapy, and report-back interventions. The interventions are generally delivered individually or by household via a professional clinician such as a psychotherapist. Behavioral modification interventions have been shown to enhance healthy household environmental modifications (47), but with little sustainment of behavior change over time (48, 49). Emergency preparedness messages generally focus on telling people how to prepare. Still, it is important to ensure that this education is delivered to encourage behavior change, and that it is translated into concrete actions (50). Although many organizations have developed interventions to address emergency preparedness communication or have devised educational interventions, inclusion of behavioral modification intervention components within the intervention can be critical in attaining concrete preparedness.

### 2.4 Conceptual framework

The Behavior Change Wheel conceptual framework illustrates how behavior change occurs as a function of social, educational, and behavioral modification interventions (51). We used this framework to define (not compare) intervention categories, as an intervention can be classified into more than one category. Interventions and intervention components function by enhancing household members’ capability, opportunity, and motivation to achieve outcome targets. These interventions are anticipated to result in improved household preparedness.

First, social interventions function by increasing opportunity for household members to achieve the desired outcome. Examples of social interventions include environmental and social restructuring and enablement. For example, a social restructuring intervention may consist of a faith community organizing a communication list for all members of a vulnerable group that includes contact information of those members and an agency or individual willing to provide disaster response aid. Enablement is another example of a social intervention component, whereby an interventionist or a community support group places the household member on a disaster registry or connects the member to a social service that provides a disaster supply kit. The disaster registry may enable rescue evacuation when required and/or the disaster supply kit may become essential for sustaining life during sheltering-in-place at the time of a disaster. These intervention components are anticipated to result in improved disaster evacuation, shelter-in-place conditions, first aid, and communication, all of which subsequently decrease morbidity and mortality or improve post-disaster functioning.

Second, educational intervention components are achieved through the Behavior Change Wheel intervention elements of education and training (51). Examples of education and training include providing information about how to create a household disaster preparedness plan and the health consequences of poor household preparedness. Videos, patient education or public health handouts, demonstrations, and checklists are examples of the educational components of an intervention; they function by enhancing memory, cognition, physical skills, knowledge, and self-efficacy, and thereby the capability of the household member to achieve the desired outcome.

Third, behavioral modification interventions may be achieved as a function of improved motivation (51). Intervention components to improve motivation may include persuasion, incentive, coercion (compensation or cost/fine), modeling, and environmental restructuring. An example of motivation modeling may involve a celebrity or a person of substantial influence in a social network modeling the value and importance of household preparedness. The intervention may motivate teenage members of the household to encourage household conformity with the positively modeled behavior.

### 2.5 Justification for inclusion of domestic violence intervention studies in this review

Despite major data gaps, it is known that domestic violence, or intimate partner violence, is endemic globally, with clusters of increased risk and incidence that meet disaster definitions, especially when associated with increased community stressors such as economic instability or as part of cascading post-disaster sequelae (52, 53). Domestic violence is based on social gender inequality, behavioral violations of social norms, and the abuse of power (54). Behavioral violations of social norms can spread like contagions (54, 55). While there are few standardized reporting systems or data sources tracking domestic violence outcomes, the United Nations estimates that over 500,000 women were killed by intimate partners or family members in 2019 (56). The clustered increases in domestic violence-related morbidity and mortality during community stress (e.g., post-disaster) meet the above-mentioned definition of disaster. Due to the number killed or injured and calls for international assistance by human rights organizations and the United Nations, domestic violence can be classified as a technological-miscellaneous-other disaster using the EM-DAT criteria. In addition, domestic violence interventions mirror household preparedness interventions, such as having an emergency evacuation and communication plan and a packed ready-to-go bag (57).

### 2.6 Study protocol

This systematic review was conducted according to a published protocol (58) and the methods outlined in the *Cochrane Handbook for Systematic Reviews of Interventions* (59). Details on criteria for considering studies for this review, types of participants, types of outcome measures, key words, search methods, data collection and analysis, data extraction and management, assessment of risk of bias, measures of treatment effect, unit of analysis issues, dealing with missing data, heterogeneity, reporting bias, and data synthesis can be found in the published protocol (58).

We included randomized controlled trials (RCTs), including individual, cluster (cRCTs), and cross-over trials; non-randomized controlled trials (nRCTs); and controlled before-after (CBAs) studies. CBAs and nRCTs were included in line with Cochrane Effective Practice and Organization of Care Group (EPOC) criteria, wherein controlled studies require more than one intervention and more than one control, contemporaneous data are collected for intervention and control groups, and selection of control sites is appropriately justified (60). We also included program evaluation studies wherein the intervention was delivered as part of a health services or social services program, participants were assigned or included in the service non-randomly, and were tested with contemporaneous comparator groups. nRCTs were included due to the unplanned and often sudden nature of disaster events; thus, we included instances of nRCTs in which the intervention was delivered just-in-time during the initial disaster onset period to participants, with a comparator group that may have been assigned non-randomly. Trial registry summaries were included. Unpublished data, conference abstracts, preprint deposits, and theses/dissertations were excluded. The search was conducted using English language terms. If a reference, abstract, or full-text report was available in a language other than English, German, or French, translation was performed.

Study participants included individuals or households as a unit of measurement. Studies with participants who were non-institutionalized, community-dwelling adults were included. Studies with participants residing in rental housing or an apartment were included. Studies of individuals who were homebound in a residential setting or under house arrest were included.

We included social support, behavioral modification, and educational interventions that may be delivered at the organizational, household, or individual level, while excluding interventions aimed at general messaging. Comparators consisted of no intervention or interventions that were passively available to any member of the public including mass media, mass public health messaging campaigns, Internet, or publicly available educational materials, with no aligned effort to distribute or translate these materials into meaningful action change or household/individual education. Mailed or otherwise passively provided physical educational pamphlets or handouts, such as handouts included with take-home materials at the end of a clinic visit but not discussed with the patient, that the authors judged were not individually tailored and likely to contain the same content as publicly available material were classified as the comparator condition.

Primary outcomes considered critical for this review included index measures of all-hazard household preparedness supplies, behaviors (including written communication and evacuation plans), and knowledge. These primary outcomes are understood to mitigate post-disaster losses, morbidity, and mortality across disasters, settings, and subpopulations, and are prioritized as meaningful to the public, to practitioners, and to policymakers (25). Because of this, indexes, composite scores, or proportions of the three primary outcomes were synthesized and considered critical to the review. Individual components considered in the definitions of primary outcomes were categorized as (a) important, but not critical, or (b) of limited importance based on the lifesaving or life-sustaining potential for each intervention in the event of a disaster (28, 61). Individual components classified as “important, but not critical” to this review were analyzed as secondary outcomes. For all-hazard household preparedness supplies, components of this composite measure considered important but not critical to the review were water, non-perishable food, prescription medications, light source, communication equipment, and first aid supplies. For all-hazard household preparedness behaviors, components of this composite measure considered important but not critical to the review were written disaster plan, written evacuation plan, written communication plan, documents, list of prescriptions, and health history. For all-hazard household preparedness knowledge, components of this composite measure considered important but not critical to the review were knowledge of how to shut off utilities, has a fire escape plan, local disaster risk knowledge, and knows the location of an emergency shelter. Secondary outcomes included health care utilization, mortality, mental health functioning, and physical functioning. We also considered adverse effects.

We used the draft search strategy for MEDLINE in our protocol as the prototype for search strategies in other databases. The search terms and Boolean operators to combine search terms are included in Appendix 1 of our published protocol (58). The database search strategy was reviewed by a Cochrane Public Health Information Specialist utilizing the PRESS checklist (62) and two librarians, resulting in minor recommended changes from the initial protocol. In addition to MEDLINE (OVID), we searched the databases and websites listed in our published protocol (58). The initial searches were run in all databases and trial registers on May 17, 2021, and updated on May 17, 2022, with the exception of the EU Clinical Trials Registers, which was searched on December 22, 2022.

Multiple authors on this team work clinically in health care and/or as first responders for the US National Disaster Medical System. The authors who completed title and abstract screening and/or full text review and risk of bias assessments varied slightly from the protocol due to personal disaster experiences and disaster deployments. All records were independently reviewed by two authors (TA and TH for the majority of the studies with mutual support from JC, SB, MC, or TL when TA or TH were unavailable) for inclusion in two stages: title and abstract screen, and full text screening. A third author (JC for the majority of studies with mutual support from SB or TL when JC was an initial screener) reviewed any differences in the first two reviewers’ determination at each stage. Any further areas of disagreement that could not be resolved by the third author were reviewed by a fourth author (SB or TL for the majority of studies with mutual support from JC when not reviewing at an earlier stage). If uncertain, the study remained included for full team consensus.

**Figure 1:**
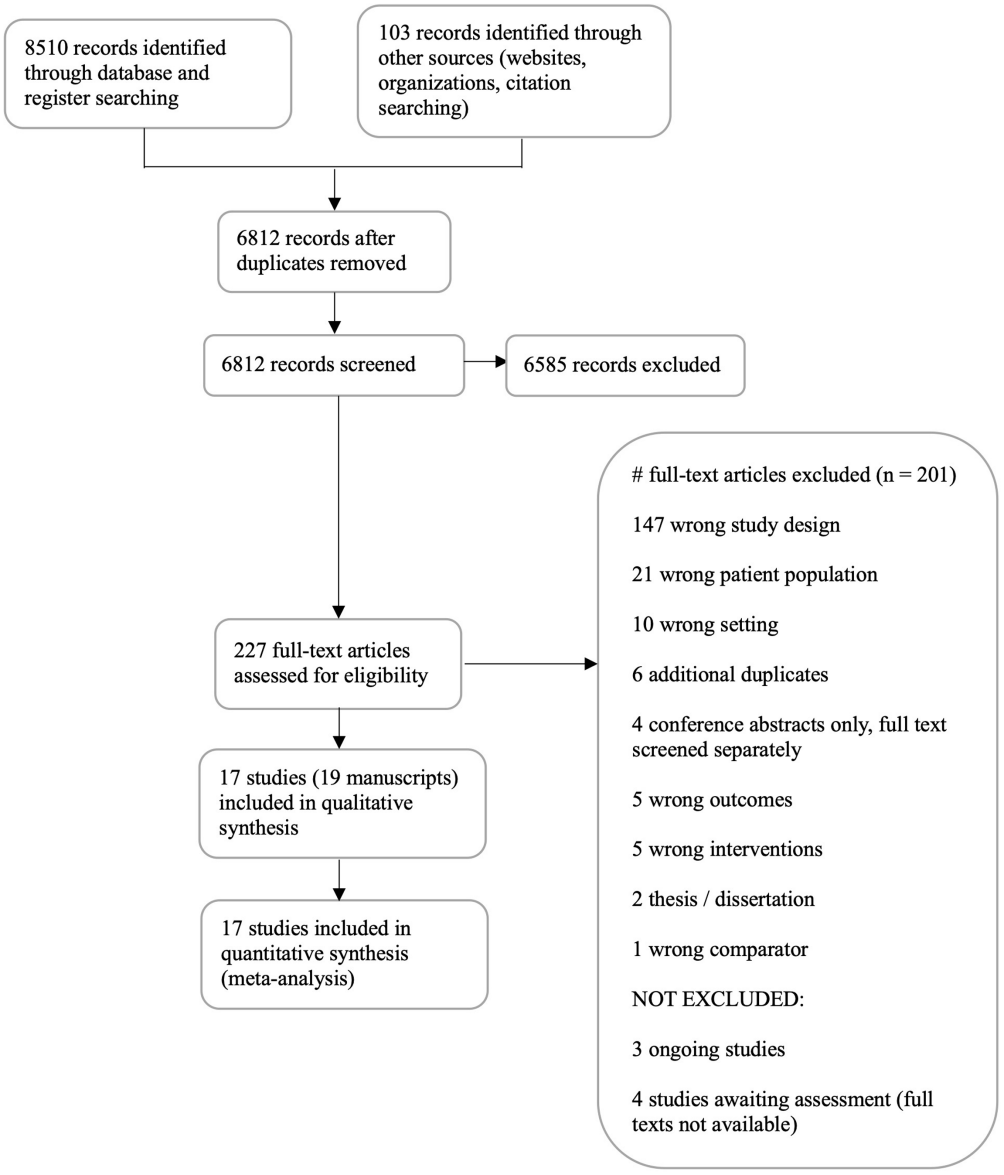
This figure shows the results of our search as of May 17, 2022. Details on criteria for considering studies for this review, types of participants, types of outcome measures, key words, and search methods can be found in the published protocol (58). Among the 17 studies included for analysis, there were 19 manuscripts.

Characteristics of excluded studies are recorded in aggregate form and reported in the PRISMA flow chart (Figure 1) (63). Characteristics of included studies are recorded in detail in Section 1 of the Online Supplementary Material. Two authors independently extracted data from the included studies (TA and TH). Any disagreements were resolved by discussion and, as needed, by a third author (JC). We developed a review-specific extraction and assessment form in Covidence based on the Cochrane Public Health Group Data Extraction and Assessment Template in Review Manager Web (64). We included an assessment of risk of bias for the included studies, which can be found in Analyses 1.1 to 1.23 and 2.1 to 2.24 in Section 2 of the Online Supplementary Material. We used either the ROBINS-I tool (used to assess risk of bias in non-randomized studies of interventions) (65), or the ‘Risk of Bias 2 (RoB2)’ tool for randomized trials (66). We assessed risk of bias for all primary and secondary outcomes.

We calculated standardized mean differences (SMDs) for continuous outcome data and odds ratios (ORs) for outcomes with binary data only, with 95% confidence intervals (CIs), using post-intervention measurements (rather than changes from baseline). For outcomes that included both continuous and binary data, we presented these as SMDs and pooled binary and continuous outcome measures by calculating SMDs from ORs.

We requested missing data from the corresponding study authors by e-mail (two attempts made for each author, 1 month apart). If the author had a profile on ResearchGate (a social networking website for researchers) or LinkedIn (a social networking site for professionals) we requested the missing data via a private message through those websites. In addition to e-mail, it was our intent to use the phone and postal address to contact authors for missing data if that contact information was presented in the published manuscript. However, for those authors who did not respond to e-mail, their telephone numbers were not reported. In addition, the postal addresses were either not reported or no longer valid with no forwarding address.

Due to the scope and nature of this review, we anticipated heterogeneity among all included studies. Because we anticipated heterogeneity among included participants, as some studies recruited from the general population and others targeted specific, vulnerable groups, we grouped all studies with participants that met our inclusion criteria and considered subgroup analysis in response to detecting statistical heterogeneity. We assessed statistical heterogeneity among included studies using the I^2^ statistic. We considered an I^2^ result of ≥50% as substantial and serious heterogeneity (67). Because no outcome included the results from 10 or more studies, funnel plot generation was not indicated to assess reporting bias.

When studies reported data on multiple outcomes, we only analyzed data for outcomes relative to our inclusion criteria. We performed both fixed-effect and random-effects meta-analyses of primary and secondary outcomes, with the intent to present the random-effects result unless there was evidence of funnel plot asymmetry, of which none was found. Separate meta-analyses were undertaken for RCTs and nRCTs assessing the same outcome, with the effect estimate derived from the meta-analysis of RCTs considered for the primary analysis. We pooled adjusted intervention effects instead of unadjusted intervention effects.

In the event of (a) limited evidence for comparison (i.e., no studies or only one study provides evidence for our pre-specified outcome); (b) intervention effects that are incompletely reported; (c) different effect measures used to measure the same outcomes that are clinically incompatible (such as time-to-event); (d) clinical/methodological diversity; or (e) statistical heterogeneity determined by I^2^ ≥ 50% (67), meta-analysis would not be indicated. If meta-analysis was not indicated for the reasons outlined above, we proceeded to synthesis without meta-analysis following methods recommended by the *Cochrane Handbook for Systematic Reviews of Interventions* Synthesis Without Meta-analysis (SWIM) guidelines (67, 68). Specifically, we reported the SMD or OR (for outcomes for which only binary data are presented) for each study and for any method used to transform binary outcome data (for outcomes where both continuous and binary data are presented) to calculate the SMD. We calculated a summary statistic of intervention effect estimates and reported a count of studies based on the direction of effect. Heterogeneity by participants and methods was analyzed. As no empirically based minimally important difference has been established for household preparedness outcome measures, we determined clinical relevance through consensus of the content expert members of the review team to guide interpretation of review results.

For each outcome, two review authors (TL and SB) used then verified (JC and MM) the GRADE approach to assess the three main outcomes, clinically most important additional three outcomes, and adverse events: all-hazard household preparedness supplies, all-hazard household preparedness behaviors, and all-hazard household preparedness knowledge; water supplies, non-perishable food supplies, prescription medication supplies, and adverse events.

We generated a Summary of Findings (Table 1) that includes the following outcomes: all-hazard household preparedness *supplies* index (critical), *behaviors* index (critical), *knowledge* index (critical), water supplies, non-perishable food supplies, prescription medication supplies, and adverse events. The Summary of Findings table was generated using GRADEpro GDT software (71).

**Table 1:** Summary of findings.

Outcomes	Anticipated absolute effects^*^ (95% CI)		Relative effect (95% CI)	Number of participants (studies)	Certainty of the evidence (GRADE)	Comments
	Risk with no or non- interactive interventions, including usual public messaging	Risk with Social support, educational, and behavioral modifications				
Preparedness Supplies	180 per 1,000	**573 per 1,000** (28 to 984)	**OR 6.12** (0.13 to284.37)	403 (2 RCTs)	⊕⊝⊝⊝Verylow^a,b,c,d^	Interventions with education, training, modeling, environmental change to motivate behavior and increase social support, enablement, persuasion, and/or coercion had no statistically significant effect on household supplies for disaster.
Preparedness Behavior	–	SMD**0.53 SD higher** (0.16 higher to 0.91 higher)	–	1,343 (6 RCTs)	⊕⊝⊝⊝Verylow^e,f,g,h^	Interventions with enablement, social restructuring, virtual or in-person environmental modification to increase social support and motivate behavior, education, training, persuasion, incentive, coercion, and/or modeling may have a small positive effect on household preparedness behaviors.
Preparedness Behavior—nRCT	–	SMD**0.82 SD higher** (0.39 higher to 1.26 higher)	–	436 (3 observational studies)	⊕ ⊕ ⊝⊝low^h,i,j^	Interventions with enablement, social restructuring, environmental modifications to increase social support and/or motivate behavior, education, training, incentive, persuasion, and/or modeling may have a small positive effect on household disaster preparedness behaviors.
Preparedness Knowledge	–	SMD**0.96 SD higher** (0.15 lower to 2.08 higher)	–	1,316 (4 RCTs)	⊕⊝⊝⊝Verylow^k,l,m^	Interventions with education, training, modeling, environmental change to motivate behavior and increase social support, enablement, persuasion, incentive and/or coercion had no statistically significant effect on knowledge of household disaster preparedness.
Preparedness Knowledge—nRCT	–	SMD**0.69 SD higher** (0.15 higher to 1.24 higher)	–	61 (1 observational study)	⊕⊝⊝⊝Verylow^d,n,o^	Based on a single non-randomized study, interventions with enablement, environmental modification to increase social support and motivate behavior, education, training, and modeling may have a small and positive effect on disaster preparedness knowledge.
Water	800 per 1,000	**954 per 1,000** (872 to 984)	**OR 5.19** (1.70 to15.84)	187(1 RCT)	⊕⊝⊝⊝Verylow^d,p,q^	Based on a single study, education, training, modeling, and environmental change to motivate behavior may have an effect on increasing water supplies in the household for disaster.
Water—nRCT	175 per 1,000	**96 per 1,000** (19 to 359)	**OR 0.50** (0.09 to 2.64)	61 (1 observational study)	⊕⊝⊝⊝Verylow^d,o,p^	Based on a single study, education has no statistically significant effect on keeping additional water supplies in the household for disaster.
Food	800 per 1,000	**954 per 1,000** (872 to 984)	**OR 5.19** (1.70 to 15.84)	187(1 RCT)	⊕⊝⊝⊝Verylow^d,r,s^	Based on a single study, education, training, modeling, and environmental change to motivate behavior may have a small effect on increasing nonperishable food supplies in the household for disaster.
Medications	290 per 1,000	**242 per 1,000** (143 to 380)	**OR 0.78** (0.41 to 1.50)	187(1 RCT)	⊕⊝⊝⊝Verylow^d,r,s^	Based on a single study, education, training, modeling, and environmental change to motivate behavior may have no statistically significant effect on keeping medication supplies in the household for disaster.
Adverse Effects—not measured	–	–	–	–	–	No adverse effects were measured relative to social, behavioral, or educational interventions meant to increase household disaster preparedness.

Social support, educational, and behavioral modifications compared to no or non-interactive interventions, including usual public messaging, for household disaster preparedness in the community dwelling population Patient or population: household disaster preparedness in the community dwelling population Setting: non-institutionalized, community households Intervention: Social support, educational, and behavioral modifications Comparison: no or non-interactive interventions, including usual public messaging. a We downgraded 1 level for serious risk of bias due to only two RCTs being included for analysis. Critical bias related to the selection of the reported results and limitations for multiple criteria, sufficient to lower confidence in the estimate of effect. b We downgraded 1 level for serious inconsistency due to substantial statistical heterogeneity, and wide confidence intervals without overlap. c We downgraded 1 level for serious indirectness due to study participants limited to a specific ethnicity or employee status, introducing substantial indirectness to the community-dwelling adult population. d We downgraded 1 level for serious imprecisions due to optimal information size not met, to a wide confidence interval crossing harm and benefit. e We downgraded 2 levels for very serious risk of bias due to seven RCTs being included for analysis, six demonstrating a high risk for bias. Crucial bias related to the outcome measurement and selection of reported results sufficient to lower confidence in the effect estimate. f We downgraded 1 level for serious inconsistency due to substantial statistical heterogeneity with missing data from 1 included RCT on this outcome. g We downgraded 1 level for serious indirectness due to participants from six RCTs limited to specific ethnicities (Jewish, Latine), disability status, gender, or intimate partner violence experience, and only 1 RCT (69) utilized a community population sampling frame, introducing substantial indirectness to the community-dwelling adult population. h Upgraded by one level because, there is evidence that the influence of all plausible confounding would reduce a demonstrated effect or suggest a spurious effect when results show no effect. i We downgraded 2 levels for serious risk of bias due to four nRCTs assessed, one with missing data related to this outcome. Critical or serious risk of bias in the measurement of outcomes for two studies. Serious risk of bias for two studies for potential deviations from intended interventions. j We downgraded 1 level for serious inconsistency due to substantial statistical heterogeneity with missing data from one nRCT in this outcome. k We downgraded 1 level for serious risk of bias due to that only two RCTs being evaluated with crucial limitations of report results selection bias. l We downgraded 1 level for serious inconsistency due to substantial statistical heterogeneity for RCTs. m We downgraded 1 level for serious indirectness because participants from three RCTs were limited to specific employment settings, disability statuses, gender, or intimate partner violence experience. One RCT (70) utilized a single healthcare setting sampling frame focused on parents of small children, introducing substantial indirectness to the community-dwelling adult population. n We downgraded 2 levels for a very serious risk of bias due to only one study evaluated with a critical risk of bias in the measurement of outcomes and a serious risk of bias in multiple other domains of confounding, classification of interventions, and deviation from intended interventions. o We downgraded 1 level for serious indirectness because study participants were limited to pregnant women, introducing substantial indirectness to the community-dwelling adult population. p We downgraded 2 levels for serious risk of bias due to crucial bias related to the selection of the reported results and limitations for multiple criteria, sufficient to lower confidence in the estimate of effect. q We downgraded 1 level for serious indirectness because study participants were a specific ethnicity in a high-income country context, introducing substantial indirectness to the community-dwelling adult population. r We downgraded 2 levels for a very serious risk of bias due to crucial bias related to the selection of the reported results and limitations for multiple criteria, sufficient to lower confidence in the estimate of effect. s We downgraded 2 levels for very serious inconsistency because study participants were limited to specific ethnicity in a high-income country context, introducing substantial indirectness to the community-dwelling adult population. *The risk in the intervention group (and its 95% confidence interval) is based on the assumed risk in the comparison group and the relative effect of the intervention (and its 95% CI).

## 3 Results

### 3.1 Description of studies

The database search returned 8,510 results. We identified 97 additional studies from the gray literature and six from citation trails. After removing duplicates, we screened 6,812 titles and abstracts and excluded 6,585. We were able to access and screen 227 full-text articles, resulting in 201 more exclusions. We identified four studies awaiting assessment due to full text not retrievable (72–75) and three potentially eligible ongoing studies (76–78). One program evaluation study was included in the full text screening but was subsequently excluded for study design prior to extraction during full team evaluation of included studies (79). One full text study was translated into English for the review team (80). We included a total of 17 studies (6,149 participants), reported in 19 manuscripts. Interrater agreement expressed as Cohen’s kappa was 0.40 for title and abstract screening and 0.37 for full text screening. See Figure 1 for our PRISMA diagram.

### 3.2 Included studies

The included studies were conducted primarily in the United States (*n* = 8, 47%) and Japan (*n* = 2, 12%), with one study conducted in each of the following countries: Australia, Haiti, Hong Kong, Iran, Israel, Nepal, and Turkey. The Turkey sample was part of one of the United States multi-site study protocols. According to the 2021 World Bank income classification, high (*n* = 5, 56%), upper-middle (*n* = 2, 22%), lower-middle (*n* = 1, 11%), and low-income (*n* = 1, 11%) countries were represented. The types of disasters targeted for the interventions in the included studies were intimate partner and gender-based violence (*n* = 6), earthquakes (*n* = 8), fires (*n* = 1), flooding (*n* = 3), war/armed conflicts (*n* = 1), and all hazards disaster preparedness (*n* = 2).

We included 12 RCTs (38, 69, 70, 81–89), one of which was a community-based cRCT (88). The remaining RCTs were parallel group design. We also included five nRCTs (43, 80, 90–92). A summary of the important characteristics of each included study is provided in the Overview of Synthesis and Included Studies (OSIS; Table 2). Nine of the 17 studies had missing data (38, 69, 80–85, 89). We received additional data for three studies (38, 80, 85).

**Table 2:** Overview and synthesis of included studies.

Author, Year Location (World Bank income group classification)	Study Design	Overall risk of bias^a^	Other key details of the intervention	Reported outcomes	Sample size (intervention/ comparator)	Time point for each outcome measured	Type of disaster risk^b^	Other variable(s)^c^
**RCTs**								
Gielen 2007 United States (high income)	RCT - parallel group	Some concerns	computer kiosk in waiting room with message tailoring/ personalized colorful 4-page report	Fire preparedness safety behaviors*/knowledge*	Parents of young children (4–66 months of age) T1 (*n* = 448/453) T2 (*n* = 384/375)	T1 = baseline T2 = 2–4 weeks after intervention	Technological - miscellaneous accident - fire	income, intervention exposure intensity
Tiwari 2012 Hong Kong (high income)	RCT - parallel group	Some concerns	one-on-one, 1 individual face-to-face interview/ education session and 12 scheduled weekly telephone calls and 24-h access to a hotline for additional social support, consisting of a non-judgmental listening, discussion with the women about their needs, offering of information when requested, and making referrals to other professionals (health/social services) and/or agencies (voluntary/ statutory) when clinically relevant.	safety promoting behavior* (hid money*, hid extra clothing*, hid extra set of keys, established code with family and friends*, asked neighbors to call police*, removed weapons, identity card*, birth certificates, school report documents, bank account numbers, marriage license, valuable jewelry, telephone numbers*)	Adult females experiencing violence T1 (*n* = 100/100) T2 (*n* = 100/100) T3 (*n* = 100/100)	T1 = baseline T2 = 3 months T3 = 9 months	Technological - miscellaneous accident - other - interpersonal violence	receiving social security assistance
Katayama 2021 Japan (high income)	RCT - parallel group	Some concerns	classroom setting, 30 min a week for four weeks, consisting of disaster preparation and evacuation behaviors	strength, flexibility, balance, evacuation distance, evacuation time*, expected obstacles, and height of climbing; general self-efficacy, quality of life, physical functioning*, mental health functioning*, social functioning	Adults (aged 56 years or older) T1 (*n* = 49/48) T2 (*n* = 45/43)	T1 = baseline T2 = 1 week	Natural - geophysical - earthquake; hydrological - flood; tsunami	N/A
McFarlane 2002 United States (high income)	RCT - parallel group	High	6 1:1 safety intervention phone calls	safety behaviors* (hid money*, hid keys, established code*, hid extra clothing*, asked neighbors to call police*, social security number*, receipts, birth certificate, driver’s license, telephone numbers*, removed weapons, bank account numbers, insurance policy number, marriage license, valuable jewelry).	Adult women T1 (*n* = 75/75) T2 (*n* = 75/74) T3 (*n* = 75/74)	T1 = baseline T2 = 3 months T3 = 6 months	Technological - miscellaneous accident - other - interpersonal violence	age
Eisenman 2009 United States (high income)	RCT - parallel group	High	classroom setting, 1-h session led by the trained promotoras from a manual designed for the study with provision of materials and group discussion	communication plan*, individual disaster supplies* (water*, food*, radio*, battery*, first aid kit*, flashlight*, extra batteries*, documents*, prescribed medicine*, pet food*, cash*, blanket*, rain gear, supplies kit*), disaster preparedness behaviors*	Adult Latines T1 (*n* = 123/119) T2 (*n* = 87/100)	T1 = baseline T2 = 3 months	all-hazards disaster preparedness	gender, marital status, perceived self-efficacy, perceived self-responsibility
Gillum 2009 United States (high income)	RCT - parallel group	High	1:1 personalized counseling session, 6 follow up phone calls over 3 months, including a discussion of safety promoting behaviors and individual needs	safety promoting behaviors*	Adult females experiencing intimate partner violence T1 (*n* = 21/20) T2 (*n* = 21/20	T1 = baseline T2 = 3 months	Technological - miscellaneous accident - other - interpersonal violence	type of violence (physical vs. nonphysical), PTSD symptoms, risk for lethal harm
Robinson-Whelen 2010 United States (high income)	RCT - parallel group	High	computer-based assessment tool with audio-video vignettes of survivors who describe their abuse and survival experiences, offer affirming messages, identify warning signs, and discuss safety promoting strategies, local abuse and safety resources after the intervention, and a cell phone preprogrammed to contact 911 or a local crisis line	safety behaviors*; abuse awareness/knowledge*, safety self-efficacy	Adult women with a disability T1 (*n* = 172/157) T2 (*n* = 126/133)	T1 = baseline T2 = 3 months	Technological - miscellaneous accident - other - interpersonal violence	stratified by type of violence experienced
Eisenman 2014 United States (high income)	RCT - parallel group+	High	classroom setting, four 2-h classes (8 h total) held twice a week for 2 weeks, consisting of in class demonstrations and homework to identify hazards	disaster preparedness behaviors*/knowledge*	Adults with intellectual and developmental disabilities T1 (*n* = 42/40) T2 (*n* = 42/40)	T1 = baseline T2 = 1 month	Natural - geophysical - earthquake Technological- miscellaneous accident- fire	age, race/ethnicity, living alone vs. with family member; Analysis stratified by primary source of support
Jassempour 2014 Iran (upper middle income)	RCT - parallel group	High	classroom setting, 3 h total over 8 weeks, consisting of 3 films of to stimulate group discussions	disaster preparedness behaviors*/knowledge*, perceived susceptibility, perceived severity, perceived benefits, perceived barriers, self-efficacy.	Adult low-income factory workers T1 (*n* = 105/111) T2 (*n* = 105/111)	T1 = baseline T2 = 16 weeks	Natural - geophysical - earthquake; hydrological - flood	N/A
Bodas 2019 Israel (high income)	RCT - Parallel group	High	internet-based with 5 arms: 1) prompted to read about future possible war and to view a 2nd Lebanon War video; 2) offered a cash reward for completing household preparedness actions; 3) preparedness promoting cognitions; 4) view short animation video made by the civil defense authorities with defense brochure download on households’ preparedness	disaster preparedness behaviors*	Adult Israli Jewish population T1 (increased threat perception *n* = 110, external reward *n* = 100, internal motivation *n* = 101, first control *n* = 35, second control *n* = 35) T2 (increased threat perception *n* = 110, external reward *n* = 100, internal motivation *n* = 101, first control *n* = 35, second control *n* = 35)	T1 = baseline T2 = 2 weeks	Technological - miscellaneous - explosion (war/armed conflicts/explosions/power outages)	gender, age, children
James 2020^ Haiti (low income)	RCT - parallel group	High	classroom setting, 3 full days of group discussion and disaster preparedness education with peer support and peer-based help seeking/giving	disaster preparedness behaviors*, depression, anxiety, post-traumatic stress*, social cohesion, willingness to provide mental health and disaster- related help to others, willingness to engage in mental health and disaster preparedness-related help-seeking	Adults T1 (*n* = 240/240) T2 (*n* = 162/146) T3 (*n* = 174/160)	T1 = baseline T2 = 3–4 months T3 = 8–9 months	Natural - geophysical - earthquake; hydrological - flood	disaster exposure, anxiety, depression, post-traumatic stress disorder, functional impairment, social cohesion, clustered within community
Taft 2015 Australia (high income)	RCT - cluster	High	one-on-one violence screening/referral and a self-completion maternal health and wellbeing checklist (given at the commencement of the three- or four-month visits)	safety behavior intervention delivered*, screening completed, referrals provided	Adult postpartum women with babies ≤12 months T1 not specified T2 not specified T3 (*n* = 1269/1352)	T1 = baseline T2 = 12 months T3 = 24 months	Technological - miscellaneous accident - other - interpersonal violence	type of violence, income, health care access, education
**nRCTs**								
Joffe 2016 United States (high income) and Turkey (upper middle income)	nRCT	Serious	classroom setting (2 three-h sessions) with self-monitoring (homework review), rehearsal (videogame play), coping skills learning, social encouragement and support and feedback	overall preparedness behavior*, earthquake preparedness, fire preparedness	Adults US T1 (*n* = 100/100) T2 (*n* = 85/72) T3 (*n* = 73/72) T4 (*n* = 66/61) Turkey T1 (*n* = 100/100) T2 (*n* = 90/101) T3 not specified T4 (*n* = 67/67)	T1 = baseline (US and Turkey sample) T2 = 1 week T3 = 3 months (US sample only) T4 = 12 months (US and Turkey sample)	natural - geophysical - earthquake and technological - miscellaneous accident - fire/earthquake	empowerment, social cohesion, trust, corruption, self-efficacy, collective efficacy, anxiety age, fatalism, location
Welton-Mitchell 2018^ Nepal (lower middle income)	nRCT - cluster step-wedge	Serious	classroom setting, 3 full days, with a manual of culturally-specific stories, group discussions, hands-on training, and provision of a disaster supply kit	disaster preparedness behaviors*, depression (PHQ), PTSD (PCL-C)*, social cohesion, help-seeking (mental health-related), help-seeking (disaster preparedness-related)	Adults T1 (*n* = 98/104) T2 (*n* = 98/103) T3 (*n* = 97/105)	T1 = baseline T2 = “some weeks after intervention” T3 not specified	Natural - geophysical - earthquake	nested within community, social cohesion
Yasunari 2011 Japan (high income)	nRCT	Critical	6 sessions added to a pre-existing childbirth class with audiovisual and disaster preparedness pamphlet provided during a childbirth education class	disaster preparedness behaviors (designated family member, family contact information*, secure items, respond to glass shattering, evacuation bag*, safe sleeping place)/knowledge (examination information, hazard map, evacuation site*, message board, location of hospital/clinic, emergency numbers)	Pregnant women in their second trimester T1 (*n* = 99/104) T2 (*n* = 99/104)	T1 = baseline T2 = 1 month after intervention/examination	Natural - geophysical - earthquake	stratified to only analyze primiparous women without disaster experience
Hamberger 2014 United States (high income)	nRCT	Critical	Clinic based counseling and provision of patient education materials, including brochures and posters, training of clinic staff, clinic policy and procedure revision.	safety promoting behaviors*; care satisfaction; conflict tactic scale; connection to community; healthcare utilization*; potential harms from violence screening (qualitative)	Adult females experiencing intimate partner violence T1 (*n* = 20/14) T2 (*n* = 13/11) T3 (*n* = 13/9)	T1 = baseline T2 = 12 months T3 = 18 months	Technological - miscellaneous accident - other - interpersonal violence	pregnancy-related health care utilization, clinic site, age
Watanabe 2020 Japan (high income)	nRCT - parallel group	Critical	classroom setting, 2 60-min classes (total 140 min) and a booklet	disaster preparedness behaviors* (11*/35 individual behaviors)/knowledge* (23 individual items)	Adult pregnant women T1 (*n* = 22/45) T2 (*n* = 21/41) T3 (*n* = 21/40)	T1 = baseline T2 = 1 month T3 = 3 months	Natural - geophysical - earthquake	weeks of gestation, education, previous preparedness

Table is organized starting with the lowest risk of bias.^a^For all assessed outcomes, unless otherwise noted.^b^classified by CRED (13).^c^used in multi-variable analysis of the outcome.*outcomes included in synthesis.^ published manuscripts from the same study protocol, tested in different populations with tailored/revised design elements. + while study authors identify this study as a crossover, only parallel group results are reported prior to crossover intervention delivery.

We identified curricular diversity across interventions and intervention delivery components. Interventions were conducted during one-on-one sessions (in-person or telephone), with lectures in classroom settings, during drills, on computer kiosks, via homework booklets, by providing pamphlets/brochures/posters (mail or direct provision), and by internet-based content such as social media sites, online videos, and self-completion surveys/checklists. Intervention delivery formats included counseling, group discussions, video games, and disaster supply kit shopping lists. Some interventions included the provision of a disaster supply kit. The Behavior Change Wheel Classification (Table 3) demonstrates if social, educational, and/or behavioral modification techniques were implemented in the multi-component interventions for each included study.

**Table 3:** Behavior change wheel intervention components.

	Enablement	Social environmental restructuring	Education	Training	Persuasion	Incentive	Coercion	Modeling	Environmental restructuring to motivate individual behavior change
Intervention domain	Social		Educational		Behavioral modification				
Bodas et al. (81)									
Intervention: Basic measures			X	X			X		
Intervention: Elevated threat perception			X	X	X		X		
Intervention: External reward			X	X		X	X		
Intervention: Internal motivation		X (virtual)	X	X			X		X (virtual)
Eisenman et al. (38)									
Comparator (Media)	X					X			
Intervention (Platica)			X	X		X		X	X
Eisenman et al. (82)									
Intervention	X	X	X	X	X	X		X	
Gielen et al. (70)									
Comparator		X	X			X			
Intervention		X	X		X	X			X
Gillum et al. (83)									
Intervention	X	X	X	X		X			X
Hamberger et al. (90)									
Intervention			X						
James et al. (69)									
Intervention	X	X	X	X		X		X	X
Jassempour et al. (84)									
Intervention	X	X	X	X	X		X	X	X
Joffe et al. (91)									
Comparator									X
Intervention	X	X	X	X	X	X		X	X
Katamaya et al. (85)									
Comparator	X	X	X	X	X			X	X
Intervention	X	X	X	X	X			X	X
McFarlane et al. (86)									
Intervention	X	X	X	X		X			X
Robinson-Whelen et al. (87)									
Intervention	X		X					X	
Watanabe et al. (80)									
Comparator			X						
Intervention	X	X	X	X				X	X
Taft et al. (88)									
Intervention	X	X	X	X					X
Tiwari et al. (89)									
Intervention	X		X	X	X	X	X		
Welton-Mitchell et al. (92)									
Intervention	X	X	X	X	X	X		X	X
Yasunari et al. (43)									
Intervention	X	X	X	X	X			X	

We identified substantive methodologic diversity regarding the level of intervention delivery. Interventions were delivered as individual-facing (70, 81, 83, 86, 89, 90), including three delivered by computer software (70, 81, 87), or aggregate group-facing. Aggregate intervention delivery included participant small group discussion, participants in workshop courses or classrooms, or included clinic staff-facing interventions. In one study it was unclear if education was delivered to the intervention group as an aggregate or individuals assigned to the intervention group (85). Further, two studies reported enabling individual participants the option to receive the intervention and complete outcome measures as a dyad with a participant-chosen support person (80, 82), while an additional study may be reasonably judged to have engaged an unmeasured additional co-parent or support (43), such as both parents contributing to a computer kiosk assessment in the emergency department waiting room (70). Studies included analysis of measures of between groups alone with individual measures (70, 85), both within and between group with individual measures (38, 43, 81–84, 86, 87, 89–91), households with one individual informant per household (69, 91, 92), and aggregate within a clinical team (88).

Comparators included non-interactive interventions, such as standard care, written or online materials, or exposure to mass media campaigns. The most active comparator condition was provided in the Eisenman study (38), where the comparison group participants were mailed disaster preparedness pamphlets, disaster kit shopping lists, and cards to assist the participants with creation of a family communication plan. The intervention group attended four 1-h classes where they received the same materials, but also participated in lectures, group discussions, and practiced carrying out preparedness actions. Similarly, in the Gielen study (70), the comparison group received a generic report with child health and household safety recommendations and the intervention group received a personalized report, tailored to the participants’ child health and household safety educational needs according to the study assessment. In all instances, the comparator condition was passively received without interpersonal interaction or computerized personalization of the information and this information was comparable to that which is publicly available as part of mass media and public education campaigns.

For studies with participants who were experiencing intimate partner violence, a written or recorded disaster plan or communications plan may increase the danger for violence if discovered by the perpetrator (86–89). Thus, we included “established a code” with our recorded disaster plan and grouped “asked neighbors to call police” with our recorded communications plans outcomes as tailored forms of the recorded disaster and communications plans that were responsive to the risk and disaster context.

### 3.3 Social context of the studies

The social context of the study and participant characteristics provide important considerations for household disaster preparedness. Housing conditions and exposure to a recent disaster were addressed in a variety of ways across study inclusion and exclusion criteria. For example, recruitment targeted those who had not experienced recent disaster in the Joffe study (91), while participants living in public housing were excluded from analyses. Alternatively, Welton-Mitchell (92) and James (69) targeted communities in active disaster recovery phases with the most extensive damage documented to recruit those in preparation for subsequent or cascading disasters. Here, 60–70% of participants had relocated to temporary structures outside their homes in the disaster recovery period, including roughly a third of the sample who had recently struggled to meet basic needs like access to food and water. Several studies targeted recruitment from low-income communities by geography (38, 69), occupation (84), or clinical service sites (70, 83, 88, 90). Studies within social contexts of high levels of interpersonal violence were also assessed, from war conflict (81) to domestic violence (83, 86–90).

### 3.4 PROGRESS health equity considerations

The acronym PROGRESS represents sample characteristics commonly used to stratify health equity considerations: place of residence, race/ethnicity/culture/language, occupation, gender/sex, religion, socioeconomic status, and social capital. We reviewed the included studies for PROGRESS considerations.

The focus of two studies were individuals with disabilities (82, 87). Race, ethnicity, and culture defined the inclusion criteria for one study focused on people who identify as Jewish living in Israel (81) and those who identify as Latine in the United States (38). Race, ethnicity, and culture measures were gathered as equity considerations for participants overall (87), those who predominantly identified as African American (70, 82, 83) or in which African American participants were over-represented compared to national demographics (86, 90), by country of origin or birth (38, 81, 89), and even caste (92). Two interventions were intentionally delivered by bilingual staff as an equity consideration (38, 86). In two studies with sites in more than one country, interventions were translated, interpreted, or read for participants where literacy levels varied in each language (69, 91, 92). Alternately, two studies excluded participants whose preferred language was not English (70, 90). Participant language spoken was also collected by McFarlane (86) and Taft (88).

Occupation was measured in Welton-Mitchell (92) as agriculture, professional, business/labor, student, or home. Laborers and clerks were included from a single factory site in Jassempour (84). Occupation was also considered as an equity consideration in measures as “currently employed” (69, 87, 89, 91); work status (not working, currently working, housekeeping) (38), or employment status (unemployed, employed at one job, employed at two jobs, disabled) (83); Tiwari (89) also measured spouse employment.

Inclusion criteria limited participation to women in seven studies (43, 80, 83, 86, 88–90). Gender or sex of participants was measured in most studies (38, 69, 81, 82, 84, 85, 92) and in child–parent dyads in Gielen (70). Women were in the substantial minority of participants only in the Jassempour study (84), which recruited from a single workplace.

Religion was measured within those with Jewish ethnicity as secular, traditional, religious, or ultra-orthodox by Bodas (81), with the majority of participants as secular; Protestant, Catholic, or other Christian in Haiti by James (69) with no clear majority; Hindu, Christian, or other in Nepal by Welton-Mitchell (92) with the majority as Hindu; reported as Christian or non-religious in Joffe (91) with the majority as non-religious.

Formal education of household member participants was measured in 11 studies (38, 70, 80, 81, 83, 84, 86, 87, 89–91) while literacy was measured as a proxy for education in Welton-Mitchell (92). Clinical team education level was measured in Taft (88). Income data was reported as a continuous variable (87), in specified dollar range categories (90), broad qualitative categories (81), or as binary cutoffs such as below or above poverty level (38, 70, 84). Tiwari (89) reported a subjective perception measure of having financial difficulties. Other equity-related measures of social capital included owning or renting a home (38, 80, 91), type of health insurance (90), or receiving public income assistance (89).

### 3.5 Moderators

We looked at four variables as possible moderators for baseline household preparedness level: age, presence of chronic illness, household composition/familial structure, and veteran status. Age was measured in 15 studies, demonstrating middle age (between 35 and 44 years) as the predominant mean age of study participants (38, 43, 69, 81, 82, 84, 86, 89, 92) and the Turkey sample of the Joffe (91) study. The mean age of the study participants designated as early adulthood (18 to 34 years) was demonstrated in the Gielen (70) and Hamberger (90) studies and as late middle age (45 to 64 years) in the US sample of the Joffe (91) study and the Taft (88) and Robinson-Whelen (87) studies. The only sample with a mean age of participants classified as late adulthood (65 years and older) was from the Katayama (85) study.

Two studies reported on the health status of their participants, measured directly or indirectly. In the Robinson-Whelen (87) sample, 46.2% of the total sample reported having an “ongoing health condition.” In the Welton-Mitchell (92) study, 17% of the total sample identified as having “poor health.”

Of the eight studies with data provided on marital/partnered status, married/coupled participants were overrepresented in three studies (88, 89, 91), and underrepresented in five studies (38, 70, 83, 87, 90). Children in the home status was reported for six studies (38, 69, 80, 81, 89, 92), with the majority of participants reporting having children and the mean number of children as two. In the Eisenman (82) study of community dwelling adults with developmental disabilities, household composition was measured as “lives alone” (29.3% total sample), “lives with roommate” (46.3% total sample), “lives with family” (22% total sample), and “lives with other” (2.4% total sample).

Veteran status is a variable that is known to impact household disaster preparedness as prior military survival skills training could function as a moderator for household preparedness level. None of the included studies reported measures about the veteran status of participants.

### 3.6 Applicability to other populations

Yasunari (43) homogenized the participant sample by only including primiparous women without disaster experience in the reported analysis after data collection. Eisenman (38) recruited participants utilizing chain referrals prior to randomization, which may have over-represented individuals from a unique social network or social connection. One study offered literacy and language accommodation (82) or interpersonal interventions that may have been influenced by the charisma or interpersonal connection of the interventionist with the participant. The potentially chaotic environment of the emergency department waiting room in the Gielen (70) study, while the similarly potentially chaotic post-disaster recovery community environment for participants in James (69) may have introduced a tradeoff between pragmatic, real-world environments and bias due to limited control of conditions. Socialized gender expectations and norms may have influenced the occupation and job participation in the study by gender in Jassempour (84).

### 3.7 Excluded studies

Studies were mainly excluded because they deviated from the intended study parameters - study design, population, or study setting. See the PRISMA diagram (Figure 1) for an aggregate list of reasons for exclusion of full text papers.

### 3.8 Risk of bias judgements in included studies

Risk of bias judgments are reported separately for RCTs, the cRCT, and nRCTs. The risk of bias summaries for each RCT outcome can be found alongside the analysis forest plot in Analyses 1.1 through 1.23 in Section 2 of the Online Supplementary Material. The risk of bias judgments for each study by domain for the nRCTs are provided in Analyses 2.1 through 2.24 in Section 2 of the Online Supplementary Material. We used the Risk of Bias 2 (RoB 2) tool (93) to evaluate the risk of bias in the included RCTs and the Risk of Bias 2 for Cluster Randomized Trials (RoB 2 CRT) tool (94) to evaluate the risk of bias in one included cRCT (88). The cRCT was not included in forest plots or pooled analysis due to clinical and measurement heterogeneity. Specifically, data in this cRCT was collected with a clinician as informant and their clinical documentation as the data source where the other studies collected directly from the household member. For the primary outcomes with multiple time points, domain judgments were consistent across initial and last reported measures.

Five RCTs were assessed as low risk of bias for the randomization process (38, 69, 70, 87, 89), four were assessed as some concerns (81, 83, 85, 86), and two were assessed as high risk (82, 84). The authors of the studies assessed as ‘some concerns’ did not explicitly comment on allocation sequence concealment and/or randomization methods. The studies judged as high risk of bias did not describe randomization methods. Eisenman (82) did not discuss concealment and results showed a gender imbalance between groups. Jassempour (84) also did not provide information on allocation sequence concealment, and certain information between groups was omitted.

Bias arising from the randomization process of the cRCT (88) was judged as high risk. The allocation sequence was not concealed from the clinical staff being assessed, but was concealed from the patient participants. There were also imbalances in the full-time or part-time work status, age, education, and home visitation program participation among the clinical staff participants. The descriptive statistics of the patient groups demonstrated possible imbalances as well. Bias arising from the timing of identification and recruitment of individual participants in relation to timing of the intervention was judged as low risk of bias, with randomization occurring prior to clinical site and patient participant recruitment.

All outcomes of six studies were assessed to be of some concern in evaluation of bias due to deviations from the intended interventions (38, 69, 81, 82, 86, 89). In the outcomes among these studies, participants and personnel were either explicitly or likely aware of the intervention assignment groups and/or no information on concealment of participants or personnel or deviations arising from the trial context were disclosed. All outcomes of four studies (70, 83, 85, 87) were assessed as low risk of bias. This domain in the cRCT (88) was judged as low risk of bias across outcomes as patients were blinded from the intervention and opportunities for deviations were limited by pre-specified guidelines only implemented at the intervention sites with nurse mentor oversight. Finally, the outcomes from Jassempour (84) were assessed as high risk of bias owing to the fact that participants in this worksite intervention were working in the same factory, making intervention information sharing (or contamination) between the intervention and control group likely to have affected the results.

All outcomes of six studies (70, 82, 83, 85, 86, 89), and the outcomes in the cRCT (88), were assessed as low risk of bias in the domain of missing outcome data. All the outcomes assessed in two of the studies (38, 69) were assessed to be of some concern when evaluating risk of bias due to substantial attrition without evidence that results were not biased by missing data. The outcomes of the remaining three RCTs (81, 84, 87) were assessed as high risk of bias due to substantial attrition coupled with the likelihood that missingness depended on the true value of the outcome.

Outcomes from only two studies (70, 85) were assessed as low risk of bias in the domain concerning measurement of the outcome, as participants were unaware of their study arm assignment and/or outcomes were measured objectively. Outcomes from five studies (38, 81–83, 89) were assessed as some concerns. This was due to assessors’ and participants’ awareness of the intervention received and/or self-reported outcome assessments. In these studies, assessment could have been influenced by knowledge of the intervention, but this was deemed unlikely. The cRCT (88) was also assessed as some concerns since the clinicians were aware of their group allocation and their documentation was utilized to assess several outcomes, which was also unlikely to have influenced the outcome. Outcomes from four studies (69, 84, 86, 87) were assessed as high risk of bias due to self-reporting with participants aware of intervention received and a likelihood of assessment being influenced by this knowledge. In McFarlane (86), investigators were not blinded to participant group assignment and also performed the intervention. Likewise, in James (69), outcome assessors were intervention facilitators. Self-reported outcomes and knowledge of intervention group status by participants were deemed likely to have influenced responses.

No outcomes of the included RCTs were assessed as having a low risk of bias in selection of the reported result. Outcomes in six studies (69, 70, 81, 82, 85, 89) were assessed as some concerns due to lack of a pre-registered trial protocol or had a pre-registered protocol that lacked an analysis plan. While in some outcomes/studies, the methods relayed sufficient detail, but it was often unclear if these methods were determined before or after initial analyses had begun. Outcomes from five studies (38, 83, 84, 86, 87) were assessed as high risk of bias due to lack of pre-registered trial protocol or analysis plan coupled with multiple outcome measurements and analyses by investigators, some explicitly aware of the intervention arm. A published protocol was available for the Taft (88) study, which was also assessed as having a high risk of bias due to multiple eligible outcome measures.

Overall, we judged the risk of bias of all assessed outcomes as high in eight RCTs and one cRCT and some concerns in outcomes from three RCTs (70, 85, 89). Studies were determined to be too biased to provide reliable evidence, however, we recommend this section be referenced by researchers in this field to strengthen future generated evidence.

We used the Risk of Bias in Non-randomized Studies of Interventions (ROBINS-I) (65) tool to evaluate the risk of bias of the five nRCTS included in our analysis. Our analysis identified two nRCTS with a serious overall risk of bias for assessed outcomes (91, 92) and three with a critical overall risk of bias for assessed outcomes (43, 80, 90).

None of the nRCTs were designated as having a low risk of bias due to confounding. The Joffe (91) and Welton-Mitchell (92) studies were designated as having a moderate risk of bias due to confounding because of differences in baseline characteristics of participants. The Hamberger (90) and Yasunari (43) studies were designated as having a critical risk of bias due to baseline differences in disaster experience and pregnancy history between the study groups, several unmeasured demographic characteristics, and possible exposure of the control group to disaster preparedness educational materials external to the study, which was also unmeasured. The Watanabe (80) study was designated as having a serious risk of bias because participants could self-select study arm and the researchers had to alter recruitment methods from the planned RCT protocol for the intervention group due to low recruitment.

The Joffe (91) study was the only nRCT designated as having a low risk of bias due to selection of participants. There were no group differences in baseline disaster preparedness and selection was based on residence in a clearly defined geographic region. The Welton-Mitchell (92) study was designated as having a moderate risk of bias because the participants were highly transient, with 70% having to relocate to a temporary shelter during data collection, and social cohesion measures differed between groups. The Watanabe (80) study was designated as having a moderate risk of bias because some spouses participated as dyads in the intervention group and some did not, and this data was not recorded. There were no dyads in the control group. Group interactions in the intervention group likely influenced the intervention and results, as compared to the control group. There were also group differences in average number of weeks pregnant between the intervention and control group.

The Yasunari (43) study was designated as having a critical risk of bias due to selection of participants because the researchers only analyzed data of primiparous participants. The Hamberger (90) study was also designated as having a critical risk of bias, but for several other reasons: (a) participants could have experienced the intervention in previous clinic visits, prior to the initial study assessment, but there was no baseline assessment; (b) most participants who were eligible to participate did not enroll in the study; (c) data collection was not continuous, and; (d) only women who were unaccompanied by their partner were selected to participate.

Of the five nRCTs, three were designated as having a low risk of bias due to classification of interventions (90–92). These studies designed interventions clearly defined by geographical location. The Watanabe (80) study was designated as having a serious risk of bias because participants self-selected their study arm, and it was unclear if switching arms was possible after allocation. The Yasunari (43) study was designated as having a critical risk of bias in this domain because the intervention sites had more participants with disaster experience enroll than the control sites, possibly reflective of overall clinic population.

Of the five nRCTs, only one (92) was designated as having a low risk of bias due to deviations from the intended interventions because fidelity checklists were utilized for manualized interventions. The other four nRCTs were designated as having a serious risk of bias. The Joffe (91) study addressed contextual and historical events that impacted the intervention and control groups alike. Yet, there were no measures of co-interventions by community groups, which were likely to have occurred and varied by geographical locations. The Yasunari (43) study did not address variations in usual practice by clinic or provider, nor did they include a formal measure of intervention fidelity. In the Hamberger (90) study, provider intervention fidelity was assumed, not measured, and, upon medical record review, missing information was found regarding how often the providers documented violence screenings, discussions of intervention materials/topics with patients, and referrals completed. In the Watanabe (80) study, some workshops were only attended by one couple when they were meant for group sharing/dialog. In these instances, the group sharing was led by the instructor instead. In addition, individual versus group participation between the first and second workshops was unclear.

All of the nRCTs were designated as having an elevated risk of bias due to missing data for assessed outcomes. The Joffe (91) study was designated as a moderate risk of bias due to attrition (up to 30%), which was also uneven by group. The Welton-Mitchell (92) study was designated as a moderate risk because baseline disaster preparedness scores were not reported. The Yasunari (43) study was designated a serious risk of bias because the post-intervention response rate was only 25.9%. The Hamberger (90) study was designated as a serious risk of bias due to incomplete reporting related to the analysis model and 35% attrition, with participants with higher educational attainment overrepresented among those lost to follow up. The Watanabe (80) study was designated as a serious risk of bias because the differences between those who consented to participate and those who did not were not reported. In addition, the differences were not reported for participants with attrition in the control group.

The only nRCT with a low risk of bias due to measurement of outcomes was the Joffe (91) study. The researchers provided readers the data collection surveys and clearly defined their observational measures. The Hamberger (90) study was designated as a moderate risk of bias due to self-report data and missing data regarding healthcare utilization outside the clinic study site. The Welton-Mitchell (92) study was designated as a serious risk of bias due to self-report data, with social desirability bias likely, and participants and data collectors were aware of group designations. The Yasunari (43) study was also designated as a serious risk of bias due to self-report data. The Watanabe (80) study was designated as a critical risk of bias due to self-report data, and we assessed a strong potential for contamination bias.

All nRCTs were designated as having an elevated risk of bias due to selection of the reported results. The Joffe (91) study, designated as a moderate risk, reported outcomes for the same protocol differently according to study site. The Watanabe (80) study was designated as a moderate risk because multiple analyses were applied, yet the analysis methods were not stipulated in a pre-specified, registered protocol. The Welton-Mitchell (92) study was assessed as a serious risk because multiple analyses not specified in the registered protocol were conducted. The Yasunari (43) study was designated as a critical risk of bias because the results for all participants were not reported. Only results for a subgroup of participants (primiparous with no disaster experience) were described in the manuscript. The Hamberger (90) study was designated as a moderate risk of bias due to selection of reported results because it was unclear which variables were controlled for in the regression models, several exploratory regression models were run with demographic variable covariates but not reported, and pregnant participants were excluded after the initial analysis.

### 3.9 Effects of interventions

The Summary of Findings (Table 1) presents social support, educational, or behavioral modification interventions with the comparator condition of no or non-interactive interventions. The interventions assessed in this review were diverse, with variations in intervention components, co-interventions, delivery format, duration, intensity, and complexity. Overall, high levels of heterogeneity (I^2^ ≥ 0.50) limited interpretation of the pooled analysis of RCT and nRCT designs for the majority of the outcomes assessed. Longitudinal measures were analyzed and reported only for studies where follow-up data were available for the outcome, for which only the last reported data was included.

### 3.10 Unit of analysis

After a clinic level intervention to change policies and standard clinic staff practice at two sites, one study used the nonrandomized intervention status at the clinic level to assign individual participants to treatment or intervention condition and measure individual outcomes over time (90). While participants were frequently included individually for knowledge assessments, the behavior and supplies measures were at the household or family level without including demographic or individual measures of all household members (70). Other studies specified limited recruitment to only one adult per household, which strengthened the ability to attribute household and individual measures to an independent participant without need for nested or dyadic considerations (69, 92).

We combined four intervention study arms to analyze as a single intervention arm in one study (81) as all the intervention arms met our inclusion criteria for intervention. Results were reported stratified by the type of abuse disclosed in one study, which we combined to calculate the overall intervention and comparator results (87). In cases where the outcome was stratified by stages of readiness, we combined the results of strata to conform to our outcome definition of enacting a disaster preparedness behavior/obtaining the supply or not yet enacting the behavior/obtaining the supply regardless of the intention, commitment, or readiness to enact the behavior or obtain the supply (38, 84).

### 3.11 Primary outcomes

#### 3.11.1 All-hazard household preparedness supplies

Preparedness supplies indices were based on checklists composed of one Jassempour (84) and three Eisenman (38) item assessments of disaster kit supplies. Overall, two RCTs (*n* = 403) were analyzed that assessed the preparedness supplies outcome as a composite, index, or proportion of all positively observed or endorsed items. When pooled, results were statistically insignificant (OR = 6.12, 95% 0.13 to 284.37; very low certainty of evidence) after the intervention versus the comparator condition. Substantial heterogeneity was also observed when pooling these results (I^2^ = 86%) with one RCT demonstrating no effect and another a large, positive effect.

#### 3.11.2 All-hazard household preparedness behaviors

Preparedness behavior indices were based on checklists or subscales composed of 3 (87), 7 (92), 15 (81, 83, 86, 89, 90), 17 (82), 20 (69), 29 (91), and 35 (80) possible items. Several behavioral indices combined obtaining or having supplies as behaviors, along with the behaviors listed in our protocol. Overall, 11 studies assessing this outcome met the inclusion criteria, but two had missing data that were not included in the pooled analysis (83, 90), leaving 9 studies (*n* = 1,779) analyzed assessing overall household preparedness behavior. Of these analyzed studies, six were RCTs and three were nRCTs. When pooled, results of the RCTs (*n* = 1,343) indicate that there may be a small positive effect (SMD = 0.53, 95% CI 0.16 to 0.91; very low certainty of evidence) after intervention versus the comparator condition. These pooled results also demonstrated heterogeneity (I^2^ = 89%) with three RCTs indicating no effect, three RCTs indicating a small positive effect of 0.91 to 0.95, and three nRCTS demonstrating a positive effect of 0.42 to 1.21.

#### 3.11.3 All-hazard household preparedness knowledge

Preparedness knowledge indices were based on tests or subscales composed of 3 (70), 5 (87), 7 (82), 10 (84), or 23 (80) items, harmonized as an overall correct score or proportion. Overall, five studies (*n* = 1,377) were included assessing indices of household preparedness knowledge, four of which were RCTs (*n* = 1,316), and one was an nRCT (*n* = 61). When RCT findings were combined, results indicate that there may be no effect (SMD = 0.96, 95% CI −0.15 to 2.08; very low certainty of evidence) after intervention versus the comparator condition. The pooled results demonstrated substantial heterogeneity (I^2^ = 99%) with two RCTs indicating no effect, two RCTs indicating a positive effect of 0.21 to 3.34 and one nRCT revealing a small, positive effect of 0.69.

### 3.12 Secondary outcomes (individual components of the primary outcomes measures)

The three primary outcomes addressed above were a composite, index, or proportion of all positively observed or endorsed items. Individual components of these primary outcome measures included here from our protocol are potable water, non-perishable food, prescription medications, light source, communications equipment, first aid kit, recorded disaster plan, recorded evacuation plan, recorded communication plan, documents, list of prescriptions, health history, knows how to turn off household utilities, has a fire escape plan, knows most likely type of disaster to occur locally, and knows location of an emergency shelter. Of these, meta-analysis of RCT results was indicated and completed only for the recorded communication plan outcome. Meta-analysis was also conducted on the pooled nRCT results for the outcome of knowing the location of an emergency shelter, for which no included RCT studies had measured. Meta-analysis was conducted on two additional individual components of the primary outcome measures which we had not planned to evaluate using meta-analysis (ready-to-go bag and recorded contact information), because data was extracted from four or more included studies.

Potable water supplies were assessed in one included RCT and one nRCT (*n* = 248). While the RCT (*n* = 187) demonstrated a positive effect of the intervention (OR = 5.19, 95% CI 1.70 to 15.84, very low certainty of evidence) the nRCT (*n* = 61) demonstrated no effect.

The non-perishable food supplies outcome was assessed in only one included RCT (*n* = 187) demonstrating a possible small effect from the intervention (OR = 5.19, 95% CI 1.70 to 15.84; very low certainty of evidence). Similarly, medications were assessed in the same RCT (*n* = 187), demonstrating no effect from the intervention (OR = 0.78, 95% CI 0.41 to 1.50; very low certainty of evidence).

One RCT (*n* = 187) and one nRCT (*n* = 61) both demonstrated no effect on the outcome of having a light source post-intervention (two studies, *n* = 248). The included RCT effect estimate odds ratio was 1.88 (95% CI 0.76 to 4.64). While pooling the results of the RCT and nRCT demonstrated low heterogeneity (I^2^ < 0.01), meta-analysis was not conducted because the nRCT demonstrated critical risk of bias.

Only one RCT (*n* = 187) addressed communication equipment (measured as having a radio) and first aid supplies as a kit. No intervention effect was observed for these outcomes (radio OR = 1.16, 95% CI 0.57 to 2.36; first aid kit OR = 1.65, 95% CI 0.90 to 3.05).

The interventions’ positive effect on the outcome of having a recorded disaster plan was supported by the data from two RCTs (*n* = 349) with substantial heterogeneity (I^2^ = 72%; OR = 4.90, CI 1.99 to 12.08) that both favored intervention and were consistent with effects observed in longitudinal follow up. Both of the studies that measured this outcome included only women with intimate partner violence exposures who established a code with others as their recorded disaster plan.

Evidence from a single nRCT (*n* = 61) study that measured the recorded evacuation plan was included with data collected at post-intervention and longitudinally, indicating no effect.

The recorded communication plan outcome was measured in three RCTs and one nRCT (total *n* = 596). Our meta-analyses of the three RCTs (*n* = 535) demonstrated evidence that social support, educational, and behavioral modification interventions increased having a recorded communications plan post-intervention (OR = 2.77, 95% CI 1.91 to 4.01) and continued longitudinally in two of these studies (*n* = 348). These studies demonstrated low heterogeneity (I^2^ < 0.01) in the pooled analysis. We did not include the nRCT in additional meta-analysis due to a critical risk of bias. Of the 596 participants in these studies, only 61 were men, as the majority of studies measuring this outcome focused on pregnant women or women experiencing intimate partner violence.

Of the three RCTs and one nRCT (four total studies, *n* = 595) that evaluated the outcome of stored documents, three studies demonstrated no effect (OR = 0.99–1.02 for the RCTs; OR = 1.5 for the nRCT) and only one RCT revealed a positive effect (OR = 3.93) with substantial heterogeneity among the RCTs (I^2^ = 76%; OR = 2.21, 95% CI 1.42 to 3.43). This finding was consistent in the longitudinal measures found in two RCTs and the nRCT as well. Thus, the pooled, positive effect of the RCTs was consistent with the findings of only one RCT and interpreted with caution due to heterogeneity.

One nRCT (*n* = 61) with a critical risk of bias assessed the intervention effect on a recorded health history, which was the only analysis in this review that favored the comparator condition both post-intervention (OR = 0.22, 95% CI 0.05 to 0.99) and upon longitudinal follow-up (OR = 0.05, 95% CI <0.01 to 0.89).

Evidence from a single RCT (*n* = 759) demonstrated the intervention improved knowledge of the most likely type of disaster to occur locally (OR = 1.63, 95% CI 1.21 to 2.20).

Evidence from two nRCTs (*n* = 264) both demonstrated no intervention effect on the outcome of knowing the location of the emergency shelter (OR = 2.28, 95% CI 0.77 to 6.71) with low heterogeneity when pooled (I^2^ = 32%). One of these nRCTs demonstrated a possible longitudinal effect (OR = 18.86, 95% CI 1.06 to 336.51). However, this longitudinal study result should be interpreted with great caution due to critical risk of bias and the wide confidence interval calculated.

Two RCTs (*n* = 349) demonstrated evidence that social support, educational, and behavioral modification interventions increased having recorded contact information post-intervention (OR = 4.16, 95% CI 2.24 to 7.76, I^2^ < 0.01) and with longitudinal follow-up for one of these RCTs. Two nRCTs corroborated this finding, with both individual studies favoring the intervention, which continued longitudinally within one of these nRCTs.

The same two RCTs (*n* = 345) that measured recorded contact information also demonstrated positive intervention effects for the ready-to-go bag outcome. Meta-analysis of the RCT results revealed acceptable statistical heterogeneity (I^2^ = 46%) and a pooled effect (OR = 4.00, 95% CI 2.08 to 7.71), which was corroborated by the findings of longitudinal measures. The results from two nRCTs both demonstrated an intervention effect that favored the intervention (OR = 2.01 to 4.25) corroborated by the longitudinal measures in one of these nRCT studies.

No data was extracted from the included studies specific to the outcomes list of prescriptions, knows how to turn off household utilities, and has a fire escape plan. Only a single study was included in our analysis for health care utilization (one nRCT, *n* = 34). No effect was observed for the intervention in relation to this secondary outcome. No data were available for the secondary outcome mortality. Only a single study was included in our analysis for physical functioning (one RCT, *n* = 88). No effect was observed for the intervention in relation to this secondary outcome. Two RCTs and one nRCT (three studies, *n* = 626) evaluated the mental health functioning outcome, all individually supporting no effect with a pooled RCT result that also demonstrated no statistically significant effect (SMD = −0.19, 95% CI −0.80 to 0.43) with high heterogeneity (I^2^ = 85%). No effect was observed for the intervention in relation to this secondary outcome.

### 3.13 Adverse effects

No adverse effects, such as interpersonal conflict among household members, stigmatization, or emotional distress were measured or reported using structured or validated quantitative methods in relation to the intervention. Our review and protocol did not intend to include observational measures of interpersonal conflict among household members, stigmatization, or emotional distress as associated with the disaster experience itself. We only intended to include these measures as a potential adverse effect of the intervention. Two included studies did measure variables such as interpersonal conflict and emotional distress, but only as observational measures in relation to the hazard or disaster exposure (69, 92). Thus, these results were excluded from our review of adverse effects.

## 4 Discussion

We intended to ascertain the overall efficacy of social support, educational, and behavioral modification interventions to improve all-hazard household disaster preparedness for the community-dwelling population. The primary outcomes examined were a composite, index, or proportion of all positively observed or endorsed items for household preparedness supplies (e.g., water, food, and prescription medication supplies), behaviors (including written communication and evacuation plans), and knowledge (e.g., knows how to turn off household utilities). Critical outcomes assessed were household preparedness supplies, behaviors, and knowledge. Additional outcomes assessed in detail and included in our Summary of Findings (Table 1) were water supplies, non-perishable food supplies, prescription medication supplies, and adverse events. We also included additional individual components of the critical outcomes and other secondary outcomes of health care utilization, mortality, mental health, and post-disaster physical functioning. Seventeen studies were included in this review. The intervention components and delivery methods of the interventions in these studies varied, introducing clinical diversity and heterogeneity, which was supported by our finding of high levels of statistical heterogeneity. While we had planned subgroup analysis, not enough studies were included that met the pre-planned criteria to complete these analyses. No sensitivity analysis was conducted due to insufficient numbers of manuscripts included that met the prespecified criteria from our protocol (58).

Overall, in studies with very low levels of certainty in the evidence, social support, educational, and behavioral modification interventions appear to not affect overall household preparedness supplies and medications; may have a small effect on household preparedness behavior and food supplies; and have none to a very small positive effect on disaster preparedness knowledge and water supplies. Our meta-analysis demonstrated evidence that favored social support, educational, and behavioral modification interventions for having a recorded communication plan and a ready-to-go bag post-intervention and longitudinally in the last measure post-intervention. Pooled analysis also indicated there was no effect on the outcome of knowing the location of an emergency shelter. The remaining outcomes were assessed based on only a single study, demonstrated substantial heterogeneity, or revealed no to little intervention effect. No studies measured adverse effects related to the intervention. Further research is needed in other populations, in addition to these studies with the majority of participants as pregnant women and women with a history of intimate partner violence.

This systematic review and meta-analysis revealed some limitations with implementing an RCT study design for this type of inquiry. These important studies were community-based, may have been co-developed with the communities of interest (69, 92), and/or conducted within chaotic disaster recovery zones. In general, RCT designs focus on internal validity while sacrificing external validity, or population generalizability. Further, the complex and often chaotic disaster context can be expected to introduce effect treatment modifiers that generate differences between enrolled RCT samples and the population. Given the nascent stage of household preparedness intervention research, there is no consensus on a single known primary outcome, or most crucial endpoint within the research community. Due to the assumed low risk of adverse effects or harms, household preparedness intervention research often begins as a phase III randomized controlled trial (assessing if the intervention is better than what is already available), bypassing earlier phases of translational research to ascertain if the intervention does what is expected, works, and to ascertain dose and feasibility. Thus, it is reasonable to anticipate that there would be bias in the measurement of outcomes with multiple outcome measurements and multiple analyses of the data. We found multiple outcomes appeared to be included as largely exploratory analyses to elucidate the priority of these outcomes for future work. Further, we found multiple analyses were also exploratory to further define those at highest risk for low disaster preparedness. For example, Robinson-Whelen (87) transparently detailed the exploratory nature of these analyses in the pre-specified analysis plan. These findings provide value to the practice and research community as a foundation for more rigorous, and less biased, designs and studies to emerge from the disaster research community.

Our Summary of Findings (Table 1) indicates that, overall, the certainty of the evidence is very low. This is mainly related to very serious risks of bias, serious indirectness, and serious imprecision. The recruitment and inclusion criteria for only three studies demonstrated directness to our population of interest of community-dwelling households (69, 91, 92). The remaining studies were restricted to clinical site, specific ages, ethnicity, gender, workplace, pregnancy, or disability status. Many of our reported critical outcomes had only one randomized clinical trial available for analysis. Additionally, several outcomes included both observational and randomized controlled trial data, which may demonstrate methodological and statistical heterogeneity that makes GRADE assessments and pooling data challenging. Due to the nature of many of the interventions, blinding of the participants was not possible, and for many of the outcomes, this led to a high risk of bias concerns.

While the overall strength of the evidence was evaluated as low to very low, we acknowledge the valuable and informative work of the included studies. The research represents the seminal work in this field and provides an important foundation for the state of the science of household preparedness intervention effectiveness and efficacy. The findings are relevant to disaster preparedness practice and research, and we encourage researchers to continue this line of research, using these studies to inform ongoing improvements in study designs. More research is warranted in populations at-risk due other health and disease conditions in both the natural history, or windows of motivation for action, associated with the natural progression of their health condition. Researchers should utilize intervention research designs that achieve health and disease state equipoise between intervention and comparison groups. Additionally, the increased risk of bias due to missing data from attrition represents a valuable research design trade-off for studies that targeted or included populations at risk for poor disaster outcomes, or those with high risk from social determinants of health like interpersonal violence exposures. To restate, it is important to recruit and include participants facing social or other barriers that make attrition likely, as these may be the individuals most at risk for poor outcomes from a disaster and are also paradoxically traditionally under-represented in formal research. Advancing methods to retain at-risk participants would strengthen disaster intervention research.

### 4.1 Potential biases in the review process

The authorship team included members who have deployed to lead, coordinate, and administer clinical care during disaster events. The interventions evaluated here have been integrated into our clinical and public health education, training, and practice as best-evidence care over our careers. As researchers, several of the authorship team have evaluated and tested household preparedness interventions in clinical and community settings using observational and non-randomized designs in studies that were not included in this review, based on the objective review criteria. We minimized bias in this review by adhering to our published protocol (58). Notwithstanding these efforts, there remains the possibility of outstanding eligible studies. Although as a team we made efforts to apply our eligibility decision rules consistently, others making these same decisions may reach different conclusions.

### 4.2 Agreements and disagreements with other studies or reviews

Our results reflect the limited number of reviews assessing interventions of household-level preparedness and the dearth of systematic reviews assessing preparedness interventions at all. Two reviews were identified, neither of which are systematic reviews. A review by Levac (3) described inconclusive findings in exploring factors that impede household emergency preparedness. Another review (95) of 23 studies of household preparedness for earthquakes found that adoption of actions that decrease vulnerability were correlated with perceptions of the hazard and alternative actions toward preparedness behaviors, demographic characteristics, and social influence.

## 5 Conclusion

Our findings suggest that household preparedness interventions may have a small positive impact on overall disaster preparedness behavior compared to no intervention or passive receipt of publicly accessible educational material alone, albeit with very low certainty of evidence. Our findings also suggest that research designs elucidating efficacy of the often pragmatic, complex, and multi-faceted social support, educational, and behavioral modification interventions present substantial methodological challenges where rigorous study design elements may not match contextual public health priority needs and resources. Future research is warranted with participants who represent the general, community-dwelling population and those among communities at highest risk for inequity and negative health outcomes after a disaster. If RCT designs are used, we recommend enhancing rigor in design by masking participants and investigators to intervention status.

Given the limitations of the RCT design in complex population health contexts, we recommend future systematic reviews of household disaster preparedness interventions also include health services research, additional non-randomized program evaluation designs, and population level time series designs with adequate spatial and temporal resolution to evaluate population health policy and mass media as household disaster preparedness interventions. Further, we found clinical practice interventions addressing safety planning for intimate partner violence exposures were well aligned with the intervention components and designs required to study all hazard household preparedness. We recommend policymakers, researchers, and clinicians expand conceptualization of intimate partner violence to consider it as a population health, technological (human-caused) type of disaster.

We recommend that national and international stakeholders, including policymakers, researchers, disaster response agency leaders, disaster responders, and healthcare professionals convene to develop a publicly available, gold-standard and pre-registered protocol and protocol template. This template could be modeled after the United States National Institutes of Health, National Institute of Environmental Health Sciences’ Rapid Acquisition of Pre- and Post-Incident Disaster Data (RAPIDD) Protocol Designer or be an expansion of the Center for Disease Control and Prevention’s Community Assessment for Public Health Emergency Response (CASPER) tool from observational to intervention design use. Given the heterogeneity of the research design elements and results we reviewed here, a gold-standard protocol tool promises to address critical methodological barriers in the field with applicability to just-in-time use to test household disaster preparedness intervention efficacy and effectiveness. We also recommend enhancing design rigor with objective measures of the study endpoints, rather than relying on participant self-report alone. These measurements may include observed behaviors in virtual reality, games, or simulation to provide an artificial disaster environment and unfolding scenarios.

We considered mass media communications as a control condition in this review. Future research studies to ascertain the efficacy of information seeking and various communication strategies that include mass media are warranted for both the general population and in tailored interventions to reach at-risk groups. While adverse effects from disaster exposure is well-described in the published literature, future research is warranted to measure adverse effects stemming from interventions addressing household disaster preparedness, such as emotional distress and financial hardship. The intervention research we evaluated here most often began as a phase III RCT (assessing if the intervention is better than what is already available). Our results indicate that earlier phases of translational research are warranted to tailor the intervention for at-risk groups and ascertain if the intervention works as expected, ascertain dose, and test initial feasibility. Intervention design and at-risk group tailoring may require fresh insights through qualitative inquiries to address intervention components and effect modifiers including facilitators and barriers to preparedness, measures of motivation, self-efficacy, personal resilience, personality traits, healthcare provider discussion of preparedness, and prior or vicarious experience with disasters. With data from these studies and insights from this review, household preparedness education, interventions, and public policies can be further developed to meet the unique needs of community members with differing education, means, and social support systems.

## Data availability statement

The original contributions presented in the study are included in the article/Supplementary material, further inquiries can be directed to the corresponding author.

## Author contributions

TA: Conceptualization, Data curation, Formal analysis, Investigation, Methodology, Project administration, Visualization, Writing – original draft, Writing – review & editing. TH: Conceptualization, Data curation, Formal analysis, Funding acquisition, Investigation, Methodology, Project administration, Resources, Supervision, Visualization, Writing – original draft, Writing – review & editing. TW-L: Conceptualization, Data curation, Formal analysis, Investigation, Methodology, Resources, Validation, Writing – original draft, Writing – review & editing. MC: Conceptualization, Data curation, Formal analysis, Investigation, Methodology, Resources, Validation, Writing – original draft, Writing – review & editing. SB: Conceptualization, Data curation, Formal analysis, Investigation, Methodology, Resources, Validation, Writing – original draft, Writing – review & editing. MM: Conceptualization, Data curation, Formal analysis, Investigation, Methodology, Resources, Validation, Visualization, Writing – original draft, Writing – review & editing. VW: Data curation, Investigation, Methodology, Resources, Software, Writing – original draft. JC: Conceptualization, Data curation, Formal analysis, Funding acquisition, Investigation, Methodology, Project administration, Resources, Software, Supervision, Validation, Visualization, Writing – original draft, Writing – review & editing.
