# Supplementary material for: Social support, educational, and behavioral modification interventions for improving household disaster preparedness in the general community-dwelling population: a systematic review and meta-analysis
Source: Front Public Health. 2024 Feb 23;11:1257714. doi: 10.3389/fpubh.2023.1257714 (PMC11003604; doi:10.3389/fpubh.2023.1257714)

***Supplementary Material***

Social Support, Educational, and Behavioral Modification Interventions for Improving Household Disaster Preparedness in the General Community-Dwelling Population: A Systematic Review and Meta-Analysis

Taryn Amberson^1†^, Tara Heagele^2†*^, Tamar Wyte-Lake^3,4^, Mary Pat Couig^5^, Sue Anne Bell^6^, Manoj J Mammen^7^, Valerie Wells^8^, Jessica Castner^9,10^

^1^Health Systems and Population Health, University of Washington, Seattle, Washington, USA

^2^Hunter-Bellevue School of Nursing, Hunter College, The City University of New York, New York, New York, USA

^3^Veterans Emergency Management Evaluation Center, Los Angeles, California, USA

^4^Deparment of Family Medicine, Oregon Health and Science University, Portland, Oregon, USA

^5^College of Nursing, University of New Mexico, Albuquerque, New Mexico, USA

^6^University of Michigan, Ann Arbor, Michigan, USA

^7^University of Rochester, Rochester, New York, USA

^8^MRC/CSO Social and Public Health Sciences Unit, University of Glasgow, Glasgow, UK

^9^Castner Incorporated, Grand Island, New York, USA

^10^University at Albany School of Public Health, Albany, New York, USA

^†^These authors contributed equally to this work and share first authorship

**Correspondence:**Tara Heagele*
[th1591@hunter.cuny.edu](mailto:th1591@hunter.cuny.edu)

**1 Characteristics of included studies**

**1.1 Bodas et al. (79)**

| ***Study characteristics Bodas et al. (79)*** | |
| --- | --- |
| Methods | RCT |
| Participants | Community dwelling adult Jewish individual participants  **Age range:** range not reported; mean and standard deviation of the age of participants who completed both rounds of questionnaires (n=381) was  42.33 (SD 15.38). For drop outs (n=121), the mean age was 38.36 (SD 13.51).  **Recruitment**: online through iPanel to existing users; opt in option, 502 participants randomly chosen (stratified random recruitment) according to preset age/gender/geographical distribution quotas. Quotas predetermined to generate a representative sample of Jewish population in Israel.  **Eligibility**: Israelis greater than 18 years of age, Jewish, and a panelist on the iPanel (N=502)  **Country**: Israel |
| Interventions | Multicomponent (n=346); 4 intervention groups: basic measures (n=35)a, elevated threat perception (n=110), external reward (n=100); internal motivation (n=101).  Participants in the 4 intervention groups all watched a 36 second video made by the civil defense authorities in Israel, and read a brochure. The elevated threat perception group also read a passage and watched a news broadcast (potentially propaganda). The external reward group was incentivized (cash prize raffle) for compliance with recommendations. The internal motivation group was exposed to automated supportive statements to increase motivation/engagement. The intervention delivery was automated via an online platform; 35/46 (76%) participants completed both rounds (baseline and 2 week follow up surveys). No additional medium/long term measurements.  **Control:** no intervention (n=35); possible exposure to disaster preparedness mass media campaign. Preparedness index instrument could have served as an educational intervention. |
| Outcomes | disaster preparedness behaviors* |
| Equity | Adult Jewish population in Israel with internet and computer skills. "Being constantly exposed to the threat of armed conflicts has developed mechanisms that suppress preparedness behavior. These mechanisms are adhering to the principles of victimization, that is, the behavior is governed mostly by fear and appraisal of control (Figure 1). Consequently, Israelis engage in preparedness behavior only when the threat becomes real and imminent, that is, when it is potentially too late. The findings suggest that this pattern of behavior is based on a conscious decision, rather than lack of awareness. In other words, Israelis actively and knowingly postpone their engagement in preparedness until they appraise the situation as one that requires immediate attention." (pp. 713-714). Non-Jewish individuals were not included [disparity not scientifically justified] Participation was solely online, limiting generalization of findings to those with internet access and computer skills. |
| Notes | aThe basic measures intervention group was listed as a control group in the published study. We included this as an intervention group due to the intent to transfer exposure to the intervention into meaningful action/change. Authors deemed it was unlikely that the video/brochure used by researchers were passively available to any member of the public.  External reward intervention group - offered "cash reward for completing as many household adjustment actions." Participants were also told that a cash prize of 500 NIS (approx. $130 US) would be raffled among participants who completed as many of the civil defense recommendations for household preparedness to armed conflicts. |

**1.2 Eisenman et al. (38)**

| ***Study characteristics Eisenman et al. (38)*** | |
| --- | --- |
| Methods | RCT |
| Participants | Adult Latine individuals living in Los Angeles County.  **Age range:** range not reported; the mean age of the intervention group (Platica) was 37.08 years, and the mean age of the control group (Media) was 36.97 years.  **Recruitment**: Respondent-driven sampling, a chain referral sampling method that uses social networks to gather a sample representative of the target population, was used to recruit participants. Community partners identified 7 Latine individuals living in the area of study as the initial participants, or “seeds.” Seeds initiate the chain referral by recruiting peers, who, in turn, recruit other peers into the study. Participants then became recruiters and were given four coupons to recruit other peers. Only the 187 participants who completed the 3-month follow-up were included in the analysis.  **Eligibility**: Adults over the age of 18 years who self-identify as Latino and live in a house or apartment in Los Angeles County. Only one adult per household was eligible to participate.  **Country**: USA |
| Interventions | Multicomponent (n=87)  The Program Para Responder a Emergencias con Preparacion (PREP) was a community based, participatory intervention lead by trained promotoras (competent, bilingual lay health workers) from the community of study. Two telephone assessments (pre intervention and at 3 months post interventions) were conducted (approximately 45 minutes each), and 1 hour in person discussion groups occurred weekly over 4 weeks.  **Control:** Also referred to as the media group (n=100); these participants were mailed culturally sensitive, written materials on preparedness developed specifically for PREP in Spanish and English. The mailer included a pamphlet, a laminated shopping card, and six perforated preprinted communication cards with instructions on how to fill them out. |
| Outcomes | communication plan*, individual disaster supplies* (water*, food*, radio*, battery*, first aid kit*, flashlight*, extra batteries*, documents*, prescribed medicine*, pet food*, cash*, blanket*, rain gear, supplies kit*), disaster preparedness behaviors* |
| Equity | This was a low-income, less-educated, mostly immigrant sample from Los Angeles, California (Eisenman et al., p. 516). New immigrants, people who do not speak the dominant language, those who are transient or illiterate and the poor who do not have internet access are often let out of mainstream disaster preparedness messaging. Latinos are less prepared than the general population, and this is only partially explained by socioeconomic disparities. They suffer disproportionately from health, social and economic consequences of disaster preparedness. "...participants in this study are poorer, and have spent less time in the United States than a representative population from a population-based survey conducted in Los Angeles prior to this study" (Glik et al., p. 6). |
| Notes | Eisenman 2009 and Glik 2014 were two reports of the same study and extracted/presented under Eisenman 2009. See Eisenman 2009 for study characteristics.  Each seed was given $25 USD For each eligible participant they recruited, and participants were given $25 USD per telephone assessment. Overall, participants could receive up to $50 USD total, even without recruitment. |

**1.3 Eisenman et al. (80)**

| ***Study characteristics Eisenman et al. (80)*** | |
| --- | --- |
| Methods | RCT Crossover |
| Participants | Individual adult participants affected by intellectual disabilities living in the community.  **Age range**: Waitlist control (n=42): M=38.4 (Range 20-62); Intervention group (n=40): M=41.5 (Range 21-68)  **Recruitment**: Researchers recruited a convenience sample of adult clients from Westside Regional Center via staff announcements at the center’s regularly scheduled activities and with posted flyers (p. 50). Several orientations were held for interested potential participants at the regional center. Here, health education staff introduced the program, provided information about the study, and invited enrollment. All of the adult clients of the center who attended the orientations were eligible and enrolled in the program (n=91), (p. 50).  **Eligibility**: To be eligible, participants had to be regional center clients, 18 years of age or older, living with family or independently with supported services in the community and not living in congregate housing, and able to speak and understand English well enough to provide informed consent (p. 50).  **Country**: USA |
| Interventions | Multicomponent (first intervention group, n=42)  The Peer-Mentored Preparedness (PM-Prep) consisted of four 2-hour classes held twice a week for 2 weeks. This intervention was led by mentors so that "participants would have (1) personal emergency plans, (2) portable and home emergency supply kits, and (3) knowledge to protect themselves from hazards in a disaster" (p. 51). The curriculum was designed for the learning needs of adults who have a developmental disability. Visual and auditory lessons predominated and were combined with interactive, hands-on activities led by peer mentors. Written material was kept to a minimum. Emphasis on working with participants in place social networks; peer mentors as co-teachers and invited primary support person of participants to attend courses with participant. Peer oriented learning environment, Interactive, hands-on activities and small group discussions led by peer mentors; homework assignments to identify home hazards. Class themes were as follows: 1) earthquake, fire, and related home hazard safety, 2) home emergency supplies, 3) personal disaster planning, including evacuation plans and communication in an emergency, 4) a review followed by an earthquake scenario exercise that allowed discussion and skills practice.  **Control:** Waitlist control (n=40) |
| Outcomes | disaster preparedness behaviors*/knowledge* |
| Equity | This study involved participants affected by intellectual and developmental disabilities (IDD). Persons with IDD are exceptionally vulnerable and "experience disproportionate risks from disasters and encounter greater obstacles at all phases of disasters, including preparedness, response, and recovery...Their overall reduced inclusion in society—characterized by social isolation, low employment rates, high poverty rates, and impaired access to health care—may amplify their susceptibilities." "The participants were given a developmentally appropriate informed consent...Health educators reviewed the informed consent with the participants line by line." |
| Notes | The Los Angeles Westside Regional Center is a state and federally funded nonprofit agency providing care coordination, health education, and resources to people with developmental disabilities living in Los Angeles to support adults living independently in the community. This study employed a waitlist control approach. The participants received a $10 USD gift card for each assessment (p. 51). $20 USD total (pretest-posttest) |

**1.4 Gielen et al. (81)**

| ***Study characteristics Gielen et al. (81)*** | |
| --- | --- |
| Methods | RCT |
| Participants | Family units (child and parent/guardian) seeking treatment from a pediatric emergency department in a level 1 pediatric trauma center serving predominantly low-income, urban families.  **Age range:** range not reported; only frequencies of categories for both children and parents/guardians.  **Recruitment**: Parents of young children were recruited at the time of the child’s ED visit regarding either an injury or a medical complaint. Study recruiters screened triage sheets to identify children who were in the study age range, and they approached potentially eligible parents in the waiting room. The parent of any child whose visit was noted with suspicion of child abuse or neglect or whose child was critically ill or injured was not approached. A computer kiosk located in the emergency department waiting room was used to assign participants to study groups, to collect baseline data, and to generate the parent reports.  To capture the full range of health and injury conditions seen over time and to minimize seasonality concerns, participants were recruited from 9/2004 through 12/2005. On the basis of an analysis of the previous year’s ED visits, 12 busiest shifts per week were selected for recruitment times.  **Eligibility**: English-speaking parent or guardian of a child between 4 and 66 months of age being seen for an injury or medical complaint, or an age- appropriate sibling of a child being seen for those reasons; living in Baltimore City; and living with the child at least some of the time (N=901)  **Country**: USA |
| Interventions | Multicomponent (n=384)  This intervention was designed to promote parents' care seat, smoke alarm and poison storage safety knowledge and behaviors. Participants in the control group were given a tailored, stage-based safety messages based on the parent behavior profile and the Precaution Adoption Process Model stage. Follow up phone calls were made by blinded interviewers at 2 and 4 weeks.  **Control:** Parents/guardians in the control group (n=375) were given personalized handout of low-literacy materials containing generic information on 4 child health related topics: development, sleep, neighborhood safety and dog bites |

**1.5 Gillum et al. (82)**

| ***Study characteristics Gillum et al. (82)*** | |
| --- | --- |
| Methods | RCT |
| Participants | Adult women seeking care in a primary care clinic for the uninsured in an urban setting, who screened positive for recent intimate partner violence (IPV)  **Age range:** range not reported; the mean age of the intervention group was 40 years, and the mean age of the control group was 46 years.  **Recruitment**: Women 18 years of age or older seeking care in a primary care clinic were screened for IPV in a private room by a study team member. Those women screening positive for recent (past year) IPV were invited to participate in the study.  **Eligibility**: Greater than or equal to 18 years of age; screened positive for recent IPV within the past year; wanted to participate in the study. It was not reported how many women were screened and positive for IPV within the past year, invited and declined/accepted the invitation to participate in the study.  **Country**: USA |
| Interventions | Multicomponent (n=21)  Personalized counseling session upon completion of initial interview, included a discussion of safety promoting behaviors and individual needs as identified by the woman. 6-phone calls over 3 months (1,2,4,6,8,10 weeks) consisted of goal setting, discussion of safety-promoting behaviors and individual needs as identified by the woman. "Referrals for community resources or assistance with primary care clinic visits were made based on need or at the research participant's request" (p. 1260).  **Control:** Participants in the control group (n=20) received health information brochures, a list of community resources, and a monthly telephone call to confirm contact information (p. 1259). |
| Outcomes | safety promoting behaviors* (Adjusted composite score for safety-promoting behavior checklist) |
| Equity | Data collection from a primary care clinic for the uninsured in an urban setting. Eighty-three percent of the participants were African American, young and middle-aged adults, half were single, sever were married, 80% met criteria for depression, 61% met criteria for PTSD, 34% in lethal danger, 56% experienced physical abuse and 95% experienced nonphysical abuse. |
| Notes | Both groups were incentivized with a $10 USD clinic credit or phone card for completing initial interview and a $15 USD clinic credit or phone card for the 3-month follow up interview. |

**1.6 Glik et al. (33)**

| ***Study characteristics Glik et al. (33)*** | |
| --- | --- |
| Methods | RCT (extracted under Eisenman 2009) |
| Participants |  |
| Interventions |  |
| Outcomes |  |
| Equity |  |
| Notes | Eisenman 2009 and Glik 2014 were two reports of the same study and extracted/presented under Eisenman 2009. See Eisenman 2009 for study characteristics. |

**1.7 Hamberger et al. (90)**

| ***Study characteristics Hamberger et al. (90)*** | |
| --- | --- |
| Methods | nRCT |
| Participants | Adult women seeking care in one of four community clinics who screened positive for recent intimate partner violence (IPV)  **Age range:** range not reported; the mean age of the intervention group was 31.5 years (SD 11.8), and the mean age of the control group was 28.0 years (SD 7.8).  **Recruitment**: A research assistant systematically/physically rotated through the four clinics and approached women who were alone in the waiting room, inviting them to complete a women's health survey. Those who agreed were taken to a private area of the clinic. The included IPV questions were adapted from the Abuse Assessment Screen to include non-pregnant women (high sensitivity, 0.93, moderate specificity, 0.55). If women provide a positive response to any of the IPV items, they were provided with a study description and invited to participate. If willing, they signed the consent.  **Eligibility**: Female patients experiencing IPV, 18 years of age and older, presenting to the four participating clinics, who were either co-habiting in a hetero- sexual, intimate partner relationship of at least 3 months duration, or reported a prior history of having done so  **Country**: USA |
| Interventions | Multicomponent (n=20)  A Patient Health Interview (60–90 minutes long) included data collection and the intervention. Participants completed the first initial research interview, the Patient Health Interview (within 2 weeks of recruitment), with subsequent interviews at 3, 6, 12 and 18 months. To facilitate safety, participants were offered the option of conducting the interviews by telephone, at the medical clinic, or a safe public location.  **Control:** Participants in the usual care/control group (n=14) were offered information about community resources and counseled to call 911 if in danger. They also received health information brochures, a list of community resources, and a monthly telephone call to confirm contact information (p. 1259). |
| Outcomes | safety promoting behaviors*; care satisfaction; conflict tactic scale; connection to community; healthcare utilization*; potential harms from violence screening (qualitative) |
| Equity | This study included only abused women; and excluded women who spoke a preferred language other than English. Participants were recruited from family medicine clinics in "low-income communities." Most participants reported at least a high school education (83 %), low income (60 % reported less than $30,000 annual income), and government-sponsored health insurance (92 %). Most rented (75 %) or lived in a residence owned by a family member (17 %)" (p. 586). Women affected by developmental delay and who preferred a language other than English were excluded. Also women who reported abuse from a same sex partner or sibling were excluded. |
| Notes | Select clinic staff were selected as IPV advocates (21 h of intense training) to support implementation and consult with clinicians and patients as needed; training of all clinical staff (physicians, nurses, physician assistants, medical assistants) at a 4 hour workshop that included information on IPV, discussion with IPV survivors, interactive training in clinical skills including IPV inquiry, intervention and primary prevention. Non-clinical staff completed a 3-hour workshop covering similar topics adapted to their clinical roles.  Incentive - $40 USD grocery vouchers for participation at 3- and 12- months, and $50 USD vouchers for completion at 18 months |

**1.8 James et al. (83)**

| ***Study characteristics James et al. (83)*** | |
| --- | --- |
| Methods | RCT |
| Participants | Adults between the ages of 18-65 years, living in metropolitan Port-au-Prince communities, who were the household decision maker.  **Age range:** Participants in the intervention group were 18-75 years of age, mean of 36 years (SD 12.7). Participants in the control group were 18-78 years of age, with a mean age of 38 years (SD 14.3).  **Recruitment**: In the selected communities, the research team collaborated with local community leaders to develop the sampling frame from which households were invited to participate in the research by trained Haitian interviewers. Interviewers approached and invited one decision making adult (using a recruitment script) from every fifth dwelling in the order encountered when walking through the community, starting from the side of the community most affected by flooding in past seasons. Gender representation attempts were made by requesting to speak with either a female or male household decision-makers at every other dwelling unit.  **Eligibility**: 18-65 years of age; household decision-maker; available to attend a 3-day intervention training  **Country:** Haiti |
| Interventions | Multicomponent (n=240)  A mental health integrated disaster preparedness intervention content was developed in collaboration with Haitian team members to be delivered over 3 days. This approach was considered best practice and culturally relevant. Haitian lay mental health workers (trained by researchers, both mental health professionals) delivered the intervention by facilitating discussion, space for sharing personal experiences and exchange of peer- support, establishing safety and practicing coping skills targeting disaster-related distress, and hands-on training in disaster preparedness and response techniques for use by participants in their own lives and to support other community members. By the end of the second day, participants were practicing providing disaster and mental health related peer support to each other.  480 participants were interviewed at baseline and 240 were randomized to the intervention and control groups. Only 144 participants completed the intervention. Total intervention group participants lost to follow up: n=66, 40 subjects lost at Time 2 and found at Time 3, 28 subjects newly lost at Time 3.  Control: Participants in the control group (n=240) may have been exposed to interventions from other NGOs, given the chronically disaster- exposed setting.  Total control group participants lost to follow up: n=80, 41 subjects lost at Time 2 and found at Time 3, 27 subjects newly lost at Time 3 |
| Outcomes | disaster preparedness behaviors*, depression, anxiety, post-traumatic stress*, social cohesion, willingness to provide mental health and disaster- related help to others, willingness to engage in mental health and disaster preparedness-related help-seeking |
| Equity | This setting was chosen due to its extreme vulnerability to disasters (earthquakes, hurricanes, flooding and landslides); potential for unemployed individuals to be over represented in this sample due to recruitment being conducted during the day. Approximately one third of participants identified as students. |
| Notes | Listed as same protocol in registry as Welton-Mitchell 2018. Extracted as separate studies because it was implemented differently at different sites.  All potential participants in the baseline interviews, including those who declined to participate, received drinks and snacks as compensation. Intervention participants received meals, materials (printed documents, note-taking materials), and a small gift likely to be useful during future natural hazards (a radio). At Time 2 and 3 interviews, all participants received snacks, drinks, and small gifts related to disaster relief (e.g., shovels, rakes, rubber boots)" (p. 2 supplementary material). |

**1.9 Jassempour et al. (84)**

| ***Study characteristics Jassempour et al. (84)*** | |
| --- | --- |
| Methods | RCT |
| Participants | Adult low-income factory workers  **Age range**: Not reported  **Recruitment**: Recruitment methods were not addressed other than "...out of the 221 participants enrolled in this study, data was collected from 105 participants in the intervention group and 111 in the control group."  **Eligibility**: Inclusion criteria was not reported.  **Country**: Iran |
| Interventions | Multicomponent (n=105)  The training program was completed in a continuous time period of 4 sessions in 8 weeks. Each session was 45 minutes long; 3 hours total over 8 weeks. Training materials included video clips, pamphlets, posters, and slides designed and produced according to the training program objectives and the appropriate stage of readiness (according to the Precaution Adoption Process Model) and to stimulate group discussion amongst participants. It was not reported who facilitated this intervention.  **Control:** Participants in the control group (n=111) did not receive an intervention. The instrument could have acted as an intervention. Potential for contamination as control and experimental groups worked at the same factory. |
| Outcomes | disaster preparedness behaviors*/knowledge*, perceived susceptibility, perceived severity, perceived benefits, perceived barriers, self-efficacy. |
| Equity | A sample of low-income factory workers in Ahwaz, Khuzestan Province, Iran. |
| Notes |  |

**1.10 Joffe et al. (91)**

| ***Study characteristics Joffe et al. (91)*** | |
| --- | --- |
| Methods | nRCT |
| Participants | Adults from communities in 2 different cities in 2 different countries  **Age range**: range not reported; the mean age of the intervention group was 50 (Seattle participants) and 41 (Izmir participants) years, and the mean age of the control group was 50 (Seattle) and 41 (Izmir) years.  **Recruitment**: In Seattle, a recruitment agency was used to recruit 100 participants from the intervention and control areas, and to recruit evenly across each age group (18-35, 36-54, 55-80), 50% male and 50% female within each age group, two-thirds home owners and one-third renters and 50% below $45,000 and 50% above $45,000 annual household income (and the equivalent respective salary in Izmir). All recruiting in Seattle was completed over the phone by the recruitment agency) (p. 1948).  In Izmir, participants were recruited in person through home visits to be culturally appropriate. Recruitment and baseline assessments were completed simultaneously at the first home visit of those who agreed to participate. Two streets (one for control and one for intervention group) were randomly selected by the agency. If a baseline assessment was completed in one household in one building, the next adjacent building was skipped. The agency aimed to recruit according to the Seattle quota.  **Eligibility**: Adults aged 18–80, one representative per household, resident of Seattle, USA or Izmir, Turkey.  **Country**: Turkey and USA |
| Interventions | Multicomponent (Turkey n=67; USA 66)  The Fix-it intervention consisted of two 3-hour workshops (6-hours total) over 2 evenings, 1 week apart. Workshops were face-face and interactive. Social encouragement and support via Facebook page for the intervention group, taking home photos and homework review. Group discussions, games and challenges were also incorporated into the workshops. Evaluation time points: baseline (1 week before intervention) and 1 week, 3 months, 12 months post intervention.  **Control:** Participants in the control group (Tukey n=67; USA 61) did not receive an intervention but did receive a home visit to complete a baseline survey and assessment of earthquake preparedness. This likely may have acted as an intervention. The Turkey sample perhaps experienced mass media exposure: "Istanbul Seismic Risk Mitigation and Emergency Preparedness Project32, the Neighborhood Disaster Support Project and the Turkish Red Crescent, among others" (Joffe et al, 2019). |
| Outcomes | overall preparedness behavior*, earthquake preparedness, fire preparedness |
| Equity | 11 participants were excluded from all post intervention analyses from the Seattle intervention group due to the fact they were living in subsidized/low-income housing and were not allowed to adopt the required preparedness measures at home (Joffe 2019). The Seattle sample was predominantly Caucasian. "Seattle and Izmir were chosen as both cities are located in highly seismic areas, are coastal with the concomitant risk of tsunami and have not endured a highly damaging earthquake in recent decades" (Joffe et al., 2019, p. 459). |
| Notes | Joffe 2016 and Joffe 2019 were two reports of the same study and extracted/presented here under Joffe 2016.  Monetary incentives are given for participation in the study. In Seattle, participants in the intervention group were paid $250 USD for completing baseline and post-intervention assessments, as well as a 6-h workshop divided over 2 days (3 h each day). Participants in the control group were given $100 USD for completing the baseline and post-intervention assessments. To ensure completion of the pretest and posttest assessments, participants in both groups were paid only after completion of the second assessment. Participants are paid $40 USD per completed follow-up assessment, at 3 and 12 months after the intervention. Study incentives for Izmir are equivalent to the monetary incentives in Seattle |

**1.11 Joffe et al. (25)**

| ***Study characteristics Joffe et al. (25)*** | |
| --- | --- |
| Methods | nRCT (extracted under Joffe 2016) |
| Participants |  |
| Interventions |  |
| Outcomes |  |

**1.12 Katayama et al. (85)**

| ***Study characteristics Katayama et al. (85)*** | |
| --- | --- |
| Methods | RCT |
| Participants | Community-dwelling Japanese adults  **Age range**: Intervention group - 56-86, mean 70.3 (SD 5.3); Control group - 63-63, mean 71 (SD 4.8)  **Recruitment**: recruited participants with the assistance of the local government through a public relations magazine. Interested participants were asked to mail a request form to investigators.  **Eligibility**: Participants must be willing/able to: (1) voluntarily participate in the study in May 2018 in Marugame city, Kagawa prefecture, Japan, (2) have no limitations on exercise placed on them by medical doctors; and (3) submit written informed consent  **Country**: Japan |
| Interventions | Multicomponent (n=43)  The Disaster Preventive Education Group consisted of weekly, 30-minute sessions over four weeks. Education and training provided participants with information on disaster occurrence mechanisms (specific to Japan) and how to prepare for and evacuate for a disaster. This was in addition to the general exercise education, with consisted of aerobic exercise and strength training by fitness instructors for 90 minutes per week over ten weeks.  **Control:** Traditional exercise control group (n=45)  Control group participants participated in a general exercise education program, with consisted of aerobic exercise and strength training by fitness instructors for 90 minutes per week over ten weeks. |
| Outcomes | strength, flexibility, balance, evacuation distance, evacuation time*, expected obstacles, and height of climbing; general self-efficacy, quality of life, physical functioning*, mental health functioning*, social functioning |
| Equity | Recruitment and availability for sessions may have resulted in targeting a higher number of retired participants who were free of job/child rearing responsibilities. There was only a small proportion of male participants in each group: 7 (16.2%) in the intervention group, 7 (15.6%) in the comparator/control group "enrolled participants in this study were thought to be more health conscious than the average person" P 7 |
| Notes |  |

**1.13 McFarlane et al. (86)**

| ***Study characteristics McFarlane et al. (86)*** | |
| --- | --- |
| Methods | RCT |
| Participants | Adult female victims of domestic violence asking for a protection order shortly after experiencing abuse.  **Age range**: Intervention group - mean 30.25 (SD 7.87); Control group - mean 34.61 (SD 9.91)  **Recruitment**: participants were recruited when they presented to a family violence unit in a large urban District Attorney's Office seeking a protection order.  **Eligibility**: adult females who spoke Spanish or English and qualified for a protection order against a "sexual intimate" were invited to participate by one of six investigators.  **Country**: USA |
| Interventions | Multicomponent (n=75)  6 1:1 safety intervention phone calls lasting an average of 9 minutes (range 3-25 minutes). The first call occurred within 48-72 hours of initial visit, followed by calls at 1,2,3,5, and 8 weeks following intake. Each call began with the safety behavior checklist, noting behaviors adopted since last contact. Strategies and coaching for adopting safety behaviors were discussed.  **Control:** No intervention (n=75)  Control group participants had access to the standard services of the district attorney's office. All applicants are routinely given the name and phone number of their intake counselor and encouraged to telephone the counselor for further assistance. These services are free of charge, offered on weekdays during business hours on a first come, first serve basis. |
| Outcomes | safety behaviors* (hid money*, hid keys, established code*, hid extra clothing*, asked neighbors to call police*, social security number*, receipts, birth certificate, driver's license, driver's license, telephone numbers*, removed weapons, bank account numbers, insurance policy number, marriage license, valuable jewelry). |
| Equity | Women who spoke a language other than English or Spanish may also have been excluded. Due to the District Attorney's Office qualification requirements, women < 18 years of age or women who were unable or unwilling to provide evidence of intimate partner assault (i.e., police or witness report, visible injury) may have been excluded. This study served an ethnically diverse population in a large urban District Attorney’s Office. |
| Notes | Women were given $20 cash USD for the first interview, a $30 USD money order for the 3-month interview and a $40 USD money order for the 6- month interview. |

**1.14 Robinson-Whelen et al. (87)**

| ***Study characteristics Robinson-Whelen et al. (87)*** | |
| --- | --- |
| Methods | RCT |
| Participants | Adult women with a disability  **Age range**: Intervention group - mean 51.78 (12.81); Control group - not reported  **Recruitment**: Participant recruitment methods included posting flyers at various social service, domestic violence/sexual assault, and disability organizations and agencies, referrals from representatives of agencies and organizations, announcements at community-based health and disability events, the researchers’ networks of disability community members at each site, and word of mouth  **Eligibility**: Women who self-identified as being affected by a disability, a physical/mental impairment that substantially limits at least one major life activity.  **Country**: USA |
| Interventions | Multicomponent (n=172 (T1), 126 (T2))  A computer-based assessment tool with audio-video vignettes of survivors who describe their abuse and survival experiences, offer affirming messages, identify warning signs, and discuss safety promoting strategies, local abuse and safety resources after the intervention, and a cell phone preprogrammed to contact 911 or a local crisis line. Most completed the program in 1-1.5 hours.  **Control:** Health awareness program (n=157 (T1), 133 (T2))  Control group participants participated in a health awareness education program. All participants were also offered local abuse and safety resources. |
| Outcomes | safety behaviors*; abuse awareness/knowledge*, safety self-efficacy |
| Equity | Women with diverse disabilities (most reported >1) who have been exposed to abuse; their median annual household income was below the established U.S. poverty line, and only one in five reported working for pay. Less than half reported using personal assistance or support and almost 75% used at least 1 assistive device. |
| Notes |  |

**1.15 Taft et al. (88)**

| ***Study characteristics Taft et al. (88)*** | |
| --- | --- |
| Methods | RCT Cluster |
| Participants | Adult postpartum women with babies ≤ 12 months  **Age range**: Intervention group - mean 34.1 (SD 4.5); Control group - mean 34 (SD 4.6) |
|  | Recruitment: This study recruited Maternal Child Health nurse teams from their participation in a previous study, and the same 8 nurse teams participated in this study. Nurse turnover within these teams is not discussed.  Eligibility: Adult postpartum women with babies ≤ 12 months who attended appointments with the Maternal Child Health nurse teams.  Country: Australia |
| Interventions | Multicomponent (n=1269)  MCH nurses facilitated one-on-one violence screening/referral and a self-completion maternal health and wellbeing checklist (given at the commencement of the three- or four-month visits).  Control: No intervention (n=1352)  Control group MCH teams received standardized, government required, one-off DV training and a manual with recommended questions for screening. |
| Outcomes | safety behavior intervention delivered*, screening completed, referrals provided |
| Equity | Participants included 8 MCH teams in the disadvantaged north-west suburbs of Melbourne. Compared to Victorian birth data, this sample in this study included fewer immigrant women from non-English speaking countries and "quite high socioeconomic status." "Mothers are at risk of domestic violence and its harmful consequences postpartum" (p. 1) in the "disadvantaged north-west suburbs of Melbourne" (p. 3). |
| Notes |  |

**1.16 Tiwari et al. (89)**

| ***Study characteristics Tiwari et al. (89)*** | |
| --- | --- |
| Methods | RCT |
| Participants | Adult community-dwelling females experiencing violence  **Age range**: Intervention group - mean 38.18 (SD 7.61); Control group - mean 37.99 (SD 6.79)  **Recruitment**: Participant recruitment occurred from a convenience sample of women using community center services (child care, recreational or social services), through personal invitation to mothers of children attending district schools and "discrete" invitations at community fairs.  **Eligibility**: Adult females in the community who screened positive for IPV, were not seeking protective orders and were able to speak/understand Cantonese or Putohghua  **Country**: Hong Kong |
| Interventions | Multicomponent (n=100)  One-on-one, 1 individual face-to-face interview/education session and 12 scheduled weekly telephone calls and 24-h access to a hotline for additional social support, consisting of a non-judgmental listening, discussion with the women about their needs, offering of information when requested, and making referrals to other professionals (health/social services) and/or agencies (voluntary/ statutory) when clinically relevant.  **Control:** No intervention (n=100)  Both control and intervention group participants had access to usual services (child care, health care and promotion, social services, etc.) provided by the community center. |
| Outcomes | safety promoting behavior* (hid money*, hid extra clothing*, hid extra set of keys, established code with family and friends*, asked neighbors to call police*, removed weapons, identity card*, birth certificates, school report documents, bank account numbers, marriage license, valuable jewelry, telephone numbers*) |
| Equity | Participants had to speak Cantonese or Putohghua to participate, potentially resulting in the exclusion of vulnerable women that do not know local dialects |
| Notes |  |

**1.17 Watanabe et al. (78)**

| ***Study characteristics Watanabe et al. (78)*** | |
| --- | --- |
| Methods | nRCT |
| Participants | Pregnant adult women  **Age range**: Intervention group - mean 33.9 (SD 5.6); Control group - mean 32.8 (SD 4.2)  **Recruitment**: Participant recruitment occurred through four hospitals. Women who attended maternity health checkups and expressed willingness to hear about the research were randomly selected and invited to participate.  **Eligibility**: Adult women 14-24 weeks pregnant at the time of initial survey (to start the program after 16th week of pregnancy), healthy pregnancy history, speak Japanese, had a partner (spouse)  **Country**: Japan |
| Interventions | Multicomponent (n=21)  Interactive workshops were provided in a classroom setting, two 60-minute classes (total 140 minutes). Participants shared photos and experiences of how they've prepared for disasters in their home, and gave presentations on what they discussed with others in the intervention group.  Participants were provided with a booklet on how to prepare for disasters.  **Control:** No intervention (n=40)  Participants of both intervention and control group received the same booklet on how to prepare for disasters. |
| Outcomes | disaster preparedness behaviors* (11*/35 individual behaviors)/knowledge* (23 individual items) |
| Equity | Women who did not speak Japanese and were not "partnered" were not represented in this study. |
| Notes |  |

**1.18 Welton-Mitchell et al. (92)**

| ***Study characteristics Welton-Mitchell et al. (92)*** | |
| --- | --- |
| Methods | nRCT, cluster step wedge |
| Participants | adults  **Age range**: Intervention group - mean 33.9 (SD 5.6); Control group - mean 32.8 (SD 4.2)  **Recruitment**: Researchers used recruitment scripts to identify eligible participants from dwelling units/homes within the preidentified sampling frame (areas with greatest earthquake damage),  **Eligibility**: one household decision-maker per household within each selected community was eligible to participate  **Country**: Nepal |
| Interventions | Multicomponent (n=98 (T1 and T2), n=97 (T3))  Participants were in a classroom setting for 3 full days, with a manual of culturally-specific stories, group discussions, hands-on skills training, and provision of a disaster supply kit.  **Control:** (n=104 (T1), n=103 (T2), n=105 (T3))  Waitlist control community. |
| Outcomes | disaster preparedness behaviors*, depression (PHQ), PTSD (PCL-C)*, social cohesion, help-seeking (mental health-related), help-seeking (disaster preparedness-related) |
| Equity | The two communities selected to participate in this study had the greatest damage from a recent earthquake and similar demographics. |
| Notes | Participant received drinks/snacks, meals, local travel compensation, intervention materials (disaster supply kit, documents, note-taking materials ) for a total cost of $15 USD as compensation. |

**1.19 Yasunari et al. (43)**

| ***Study characteristics Yasunari et al. (43)*** | |
| --- | --- |
| Methods | nRCT |
| Participants | Pregnant women in their second trimester  **Age range**: Intervention group - 17-45; Control group - 21-41  **Recruitment**: Hospital/clinic nurses recruited participants from childbirth classes  **Eligibility**: Pregnant women in their second trimester of pregnancy  **Country**: Japan |
| Interventions | Multicomponent (n=99)  15-minute intervention lectures were incorporated into 6 childbirth classes with audiovisual and disaster preparedness pamphlets. Participants were encouraged to look at hazard maps and evacuation routes near their homes, instructed on how to create disaster kits and how to increase the safety inside of their homes.  **Control:** No intervention (n=104) |
| Outcomes | disaster preparedness behaviors (designated family member, family contact information*, secure items 1, secure items 2, respond to glass shattering, evacuation bag*, safe sleeping place)/knowledge (6 items: examination information, hazard map, evacuation site*, message board, location of hospital/clinic, emergency numbers) |
| Equity | This study was conducted in a geographical area considered high risk for earthquake disasters. |
| Notes |  |

*Outcomes included in synthesis; SD=standard deviation; USD=United States Dollars; ED=Emergency department; IPV=Intimate partner violence; MCH=Maternal Child Health; nRCT=non-randomized control trial; RCT=randomized control trial. See Table 3, Behavior Change Wheel Classification of Intervention Components, for details on multicomponent interventions.

**2 Assessment of risk of bias for the included studies**

**2.1 Analysis 1.1 – intervention vs comparator – RCTs, outcome 1: preparedness supplies**


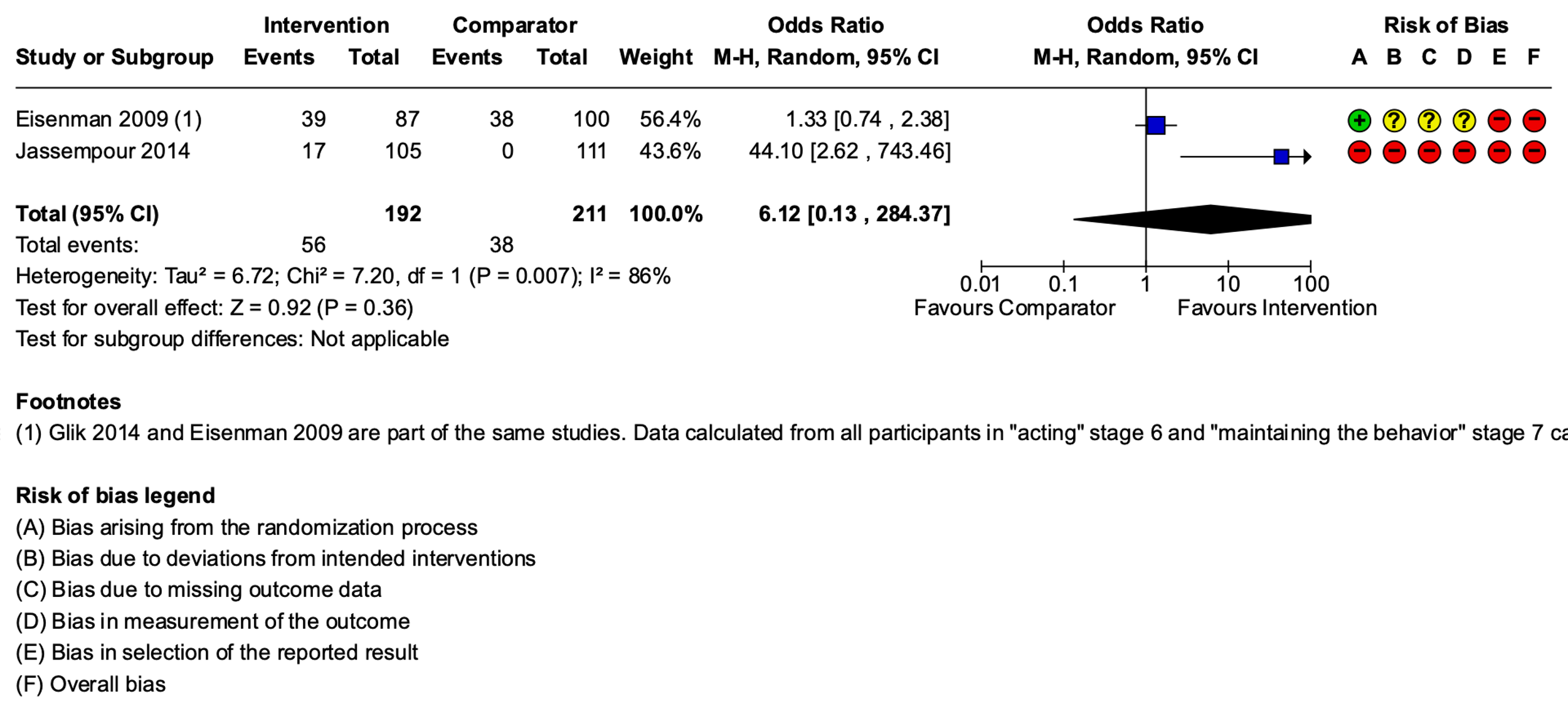


**2.2 Analysis 1.2 - intervention vs comparator – RCTs, outcome 2: preparedness behavior**


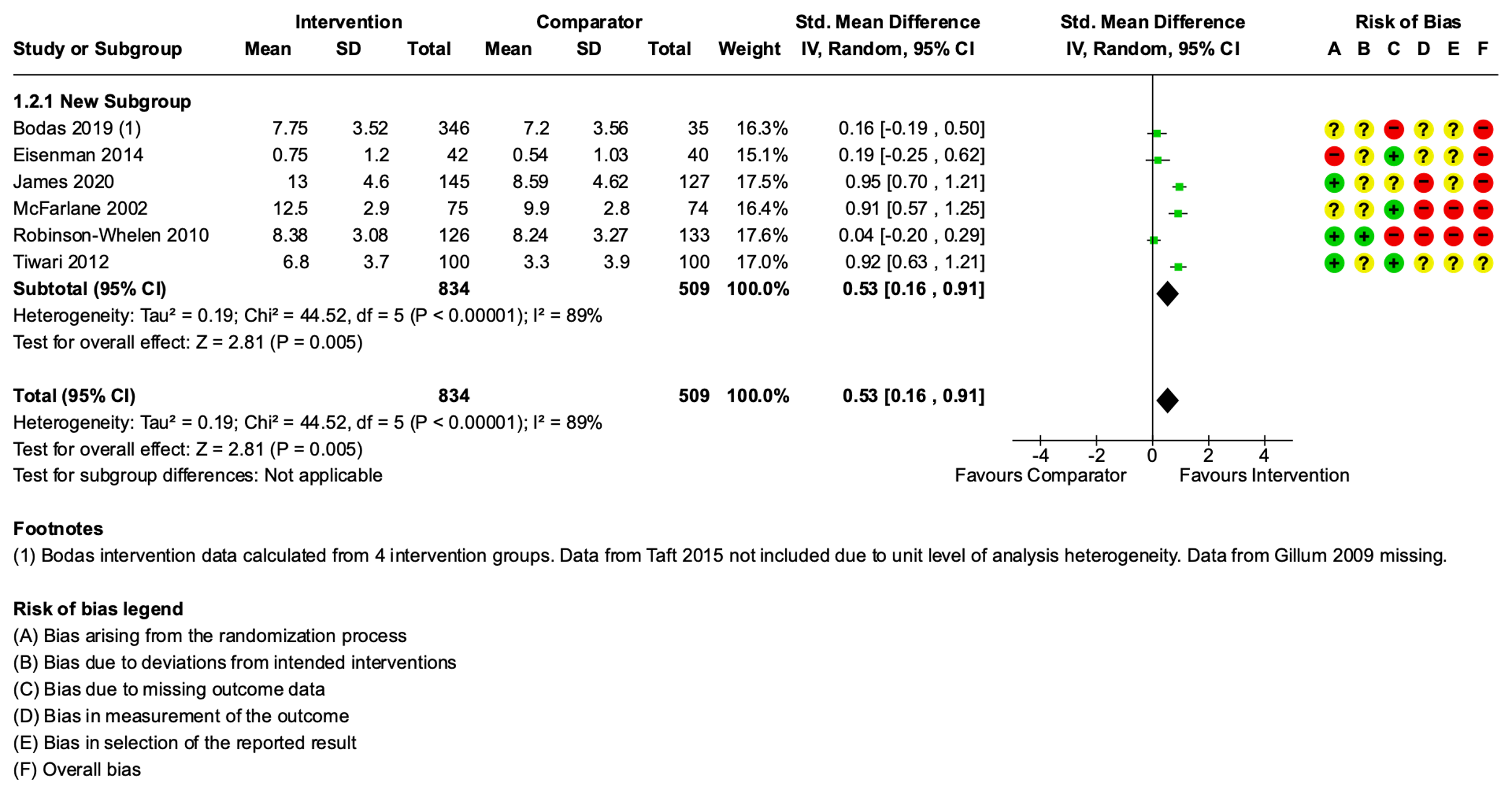


**2.3 Analysis 1.3 - intervention vs comparator – RCTs, outcome 3: preparedness behavior (last measured)**


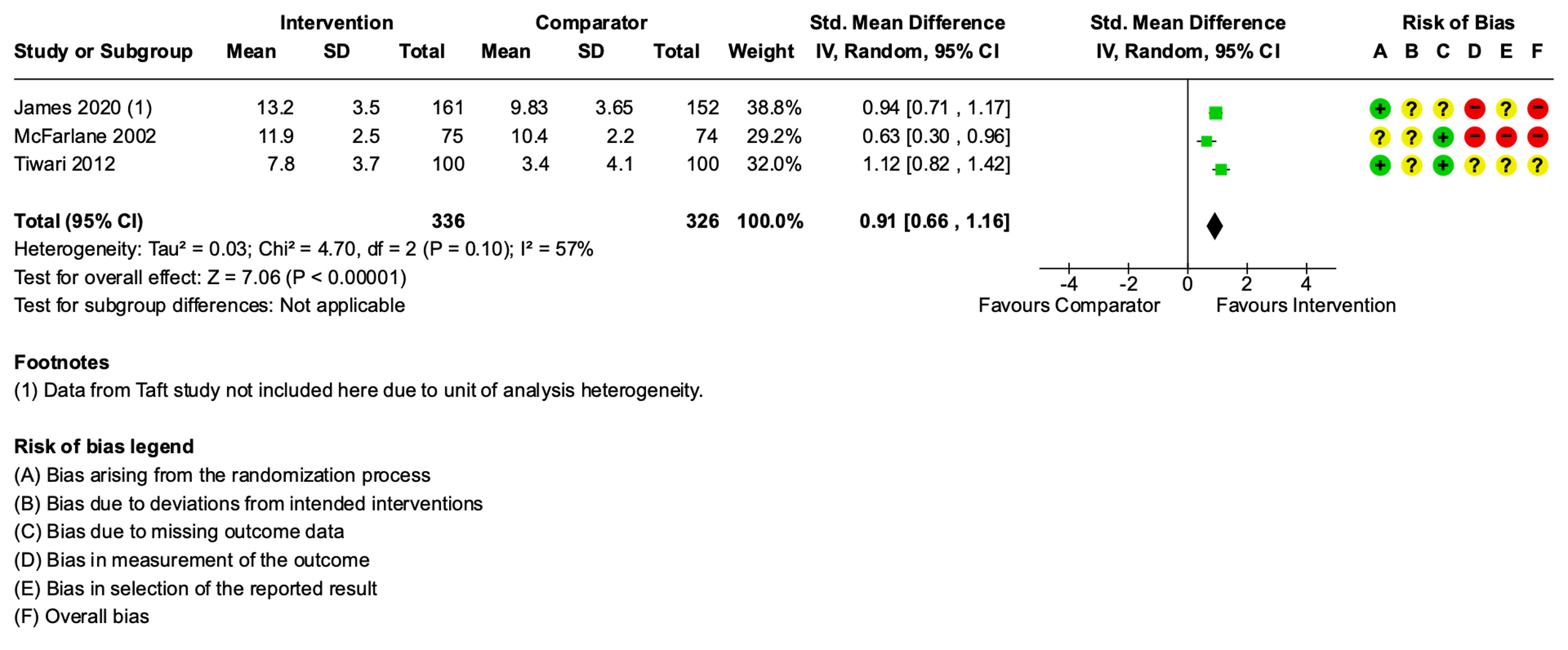


**2.4 Analysis 1.4 - intervention vs comparator – RCTs, outcome 4: preparedness knowledge**


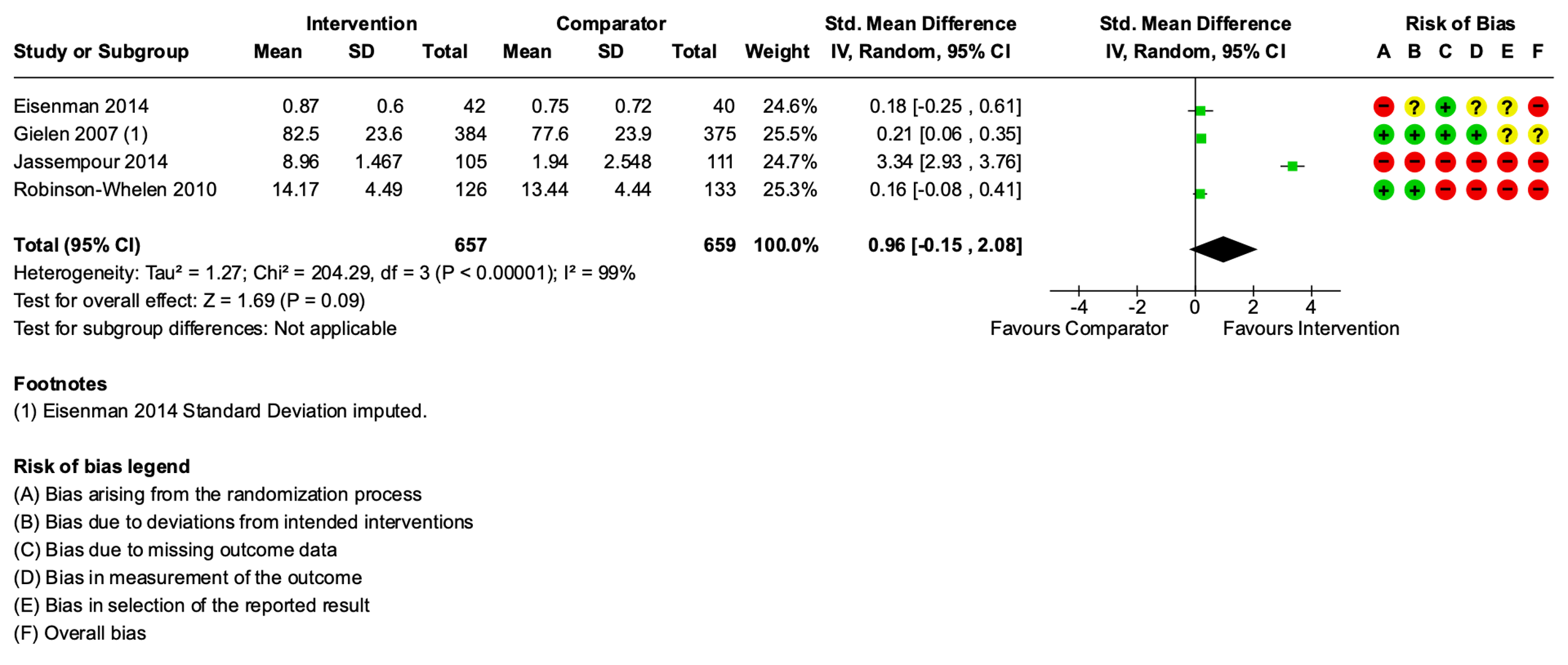


**2.5 Analysis 1.5 – intervention vs comparator – RCTs, outcome 5: potable water**


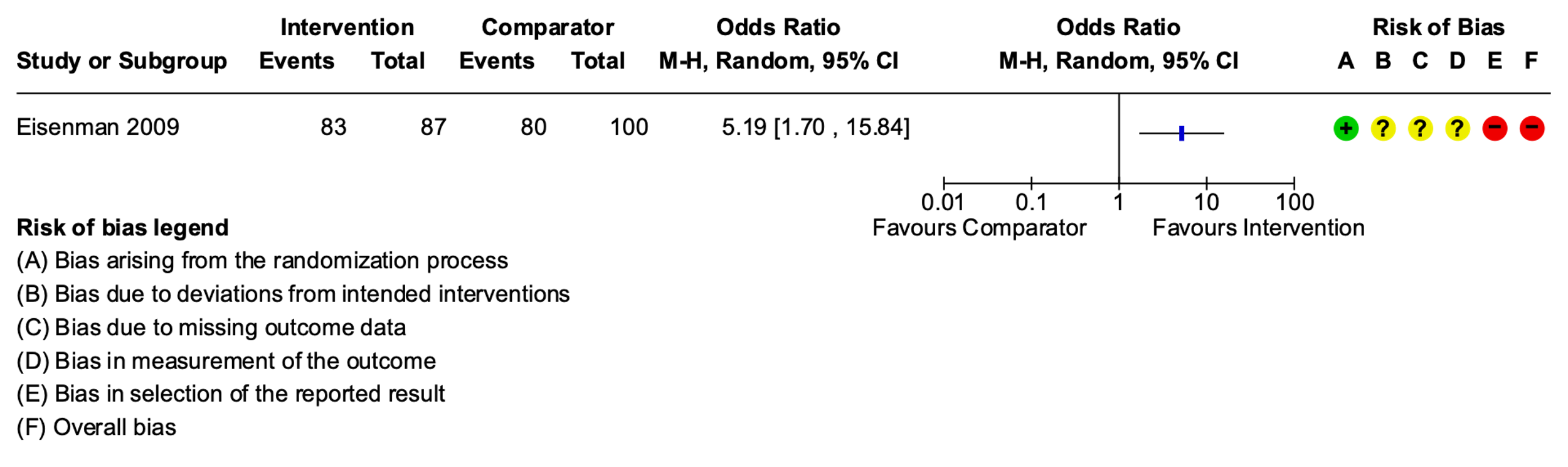


**2.6 Analysis 1.6 – intervention vs comparator – RCTs, outcome 6: non-perishable food**


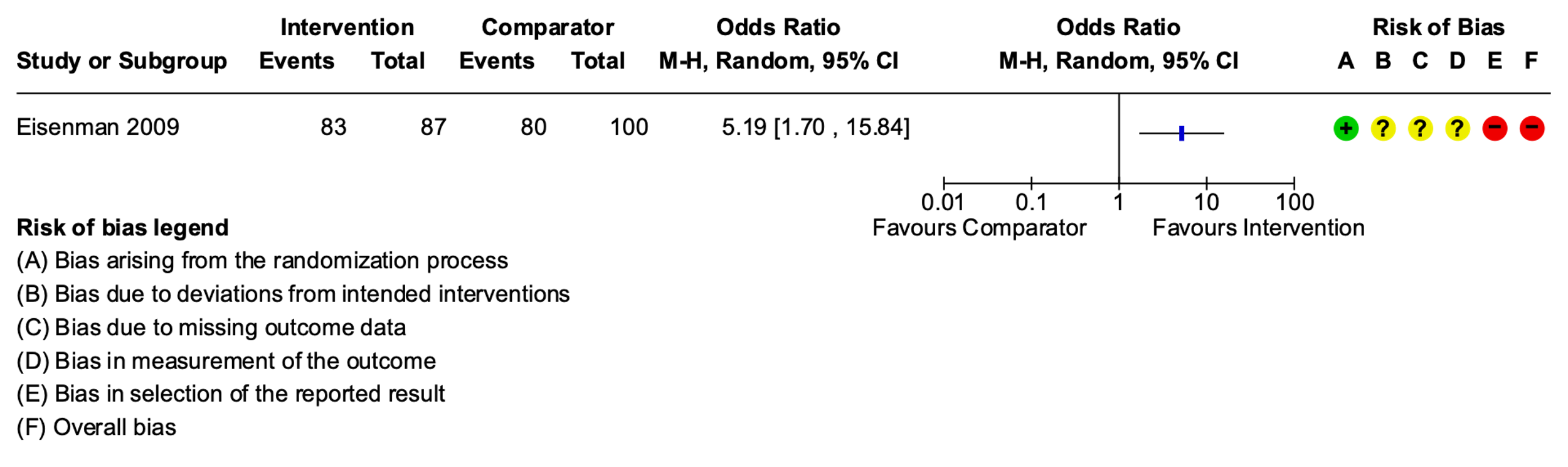


**2.7 Analysis 1.7 – intervention vs comparator – RCTs, outcome 7: medications**


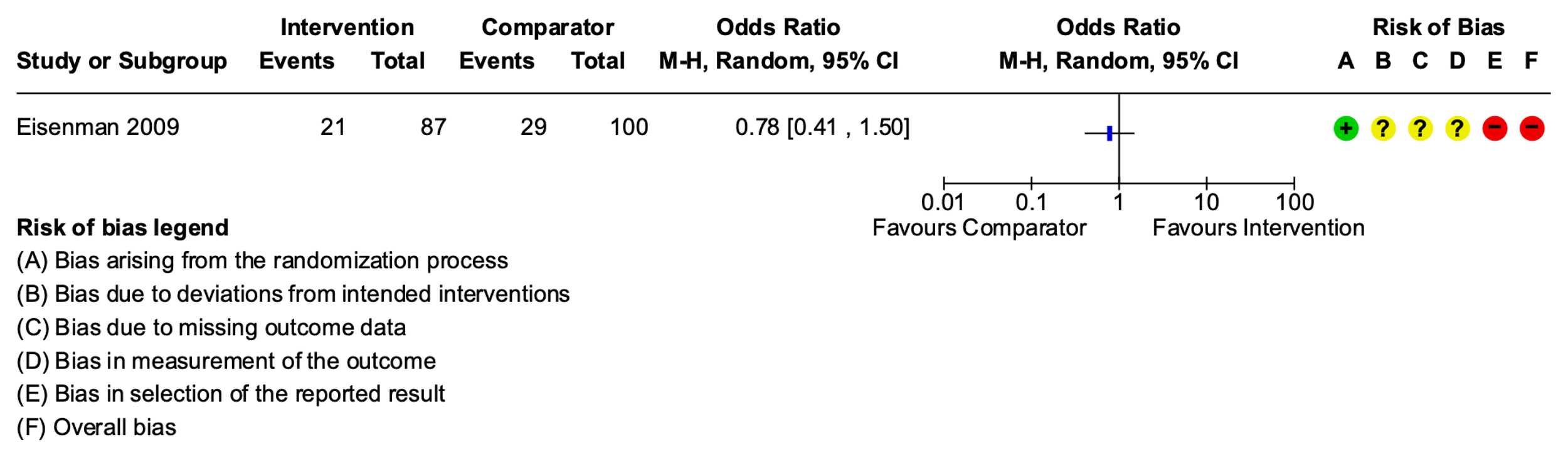


**2.8 Analysis 1.8 – intervention vs comparator – RCTs, outcome 8: light source**


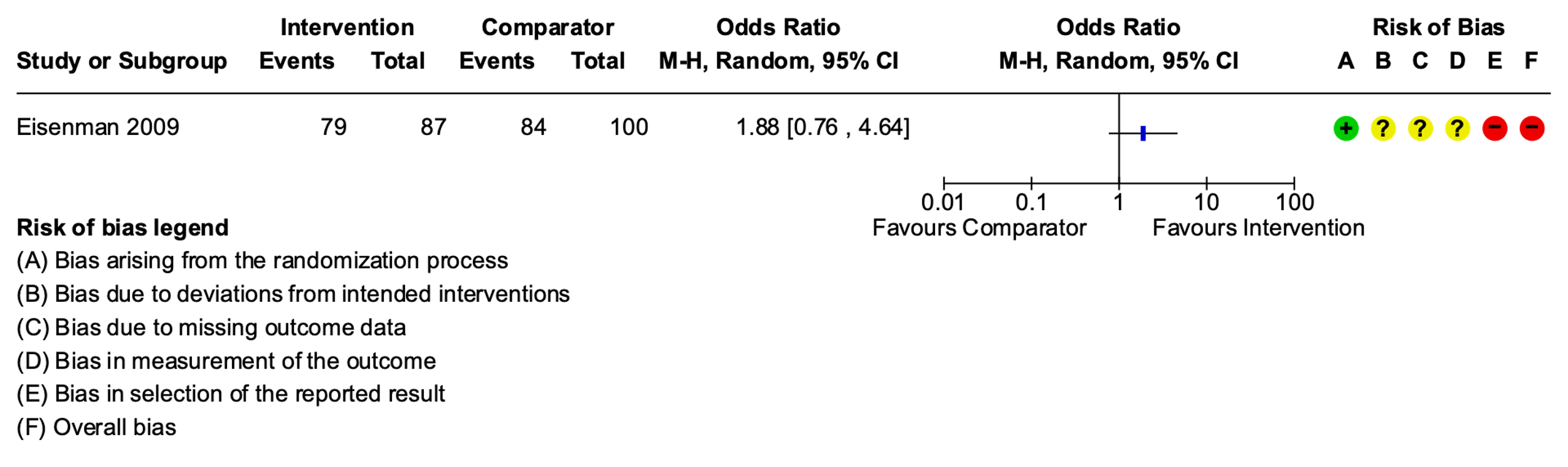


**2.9 Analysis 1.9 – intervention vs comparator – RCTs, outcome 9: radio**


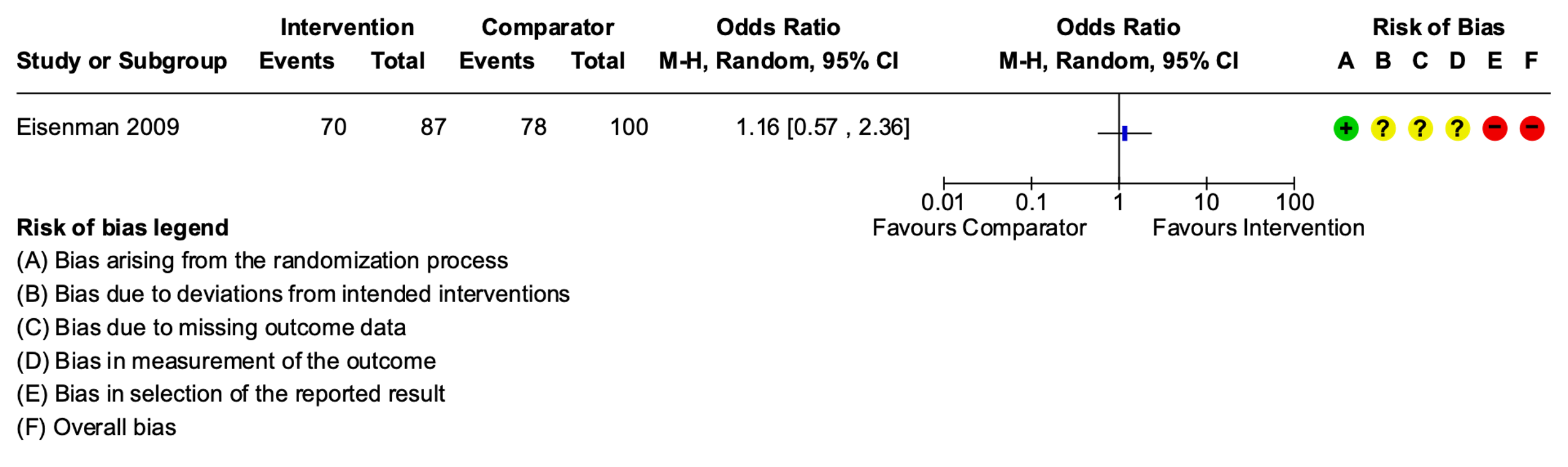


**2.10 Analysis 1.10 – intervention vs comparator – RCTs, outcome 10: first aid kit**


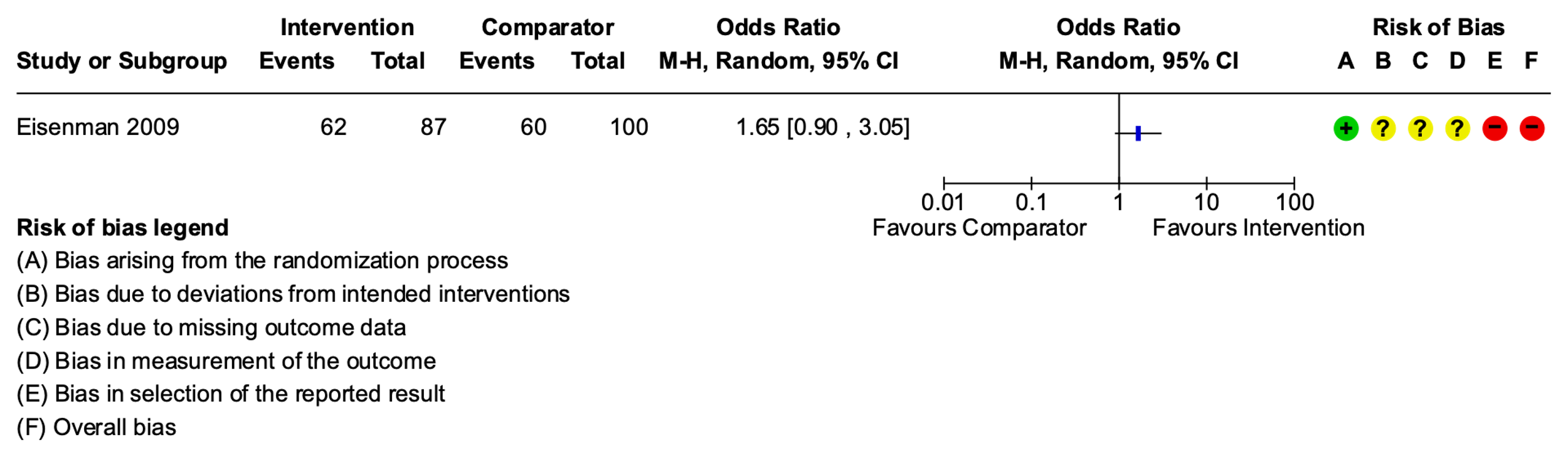


**2.11 Analysis 1.11 – intervention vs comparator – RCTs, outcome 11: recorded disaster plan**


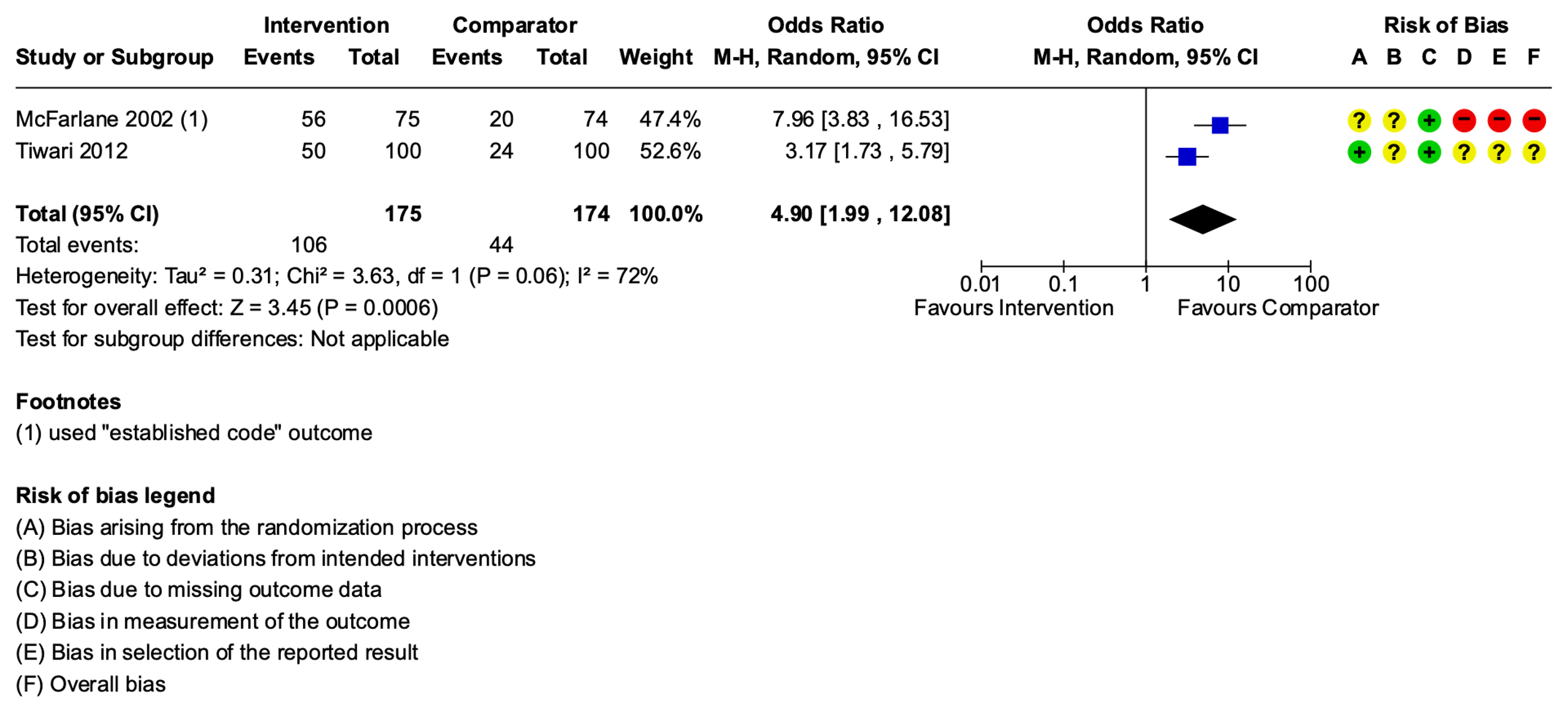


**2.12 Analysis 1.12 – intervention vs comparator – RCTs, outcome 12: recorded disaster (last measured)**


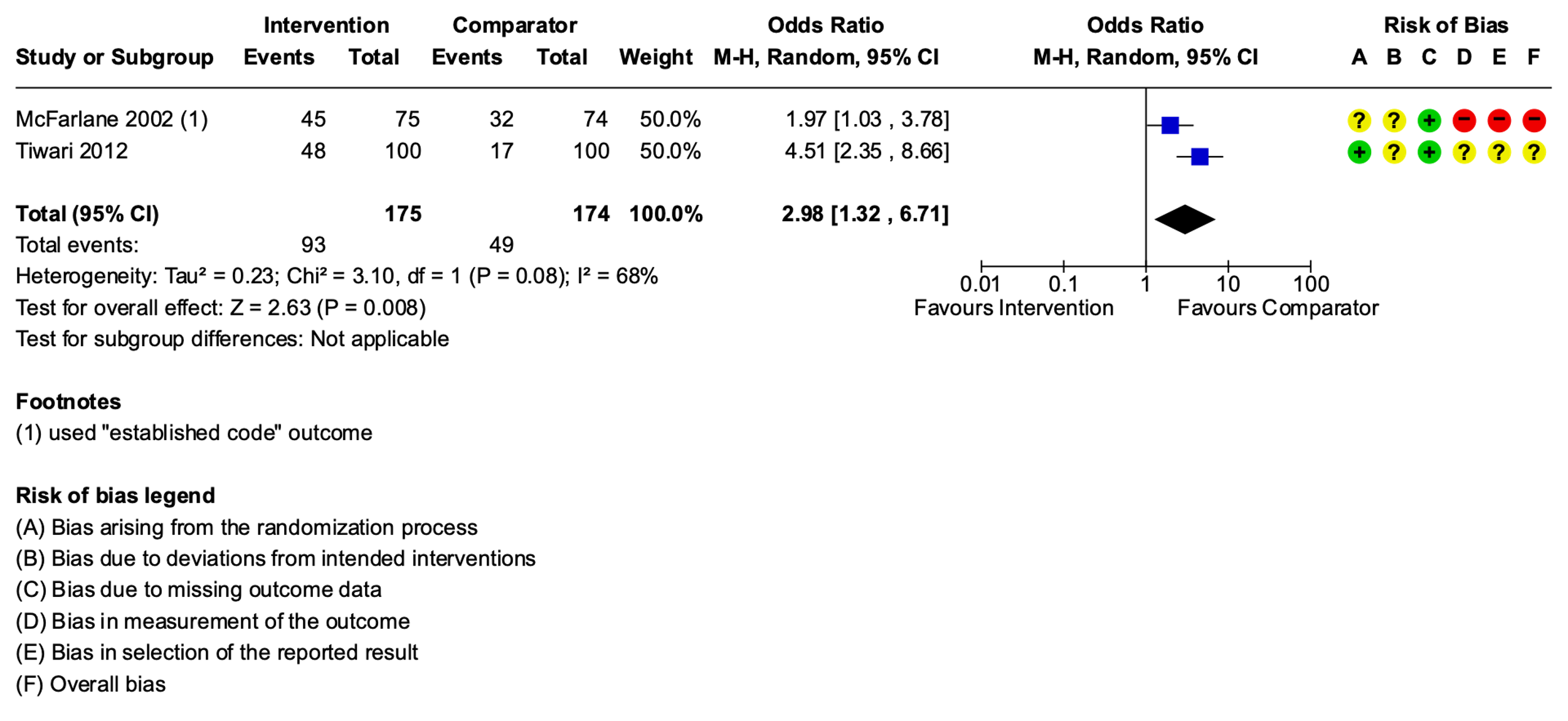


**2.13 Analysis 1.13 – intervention vs comparator – RCTs, outcome 13: recorded communication plan**


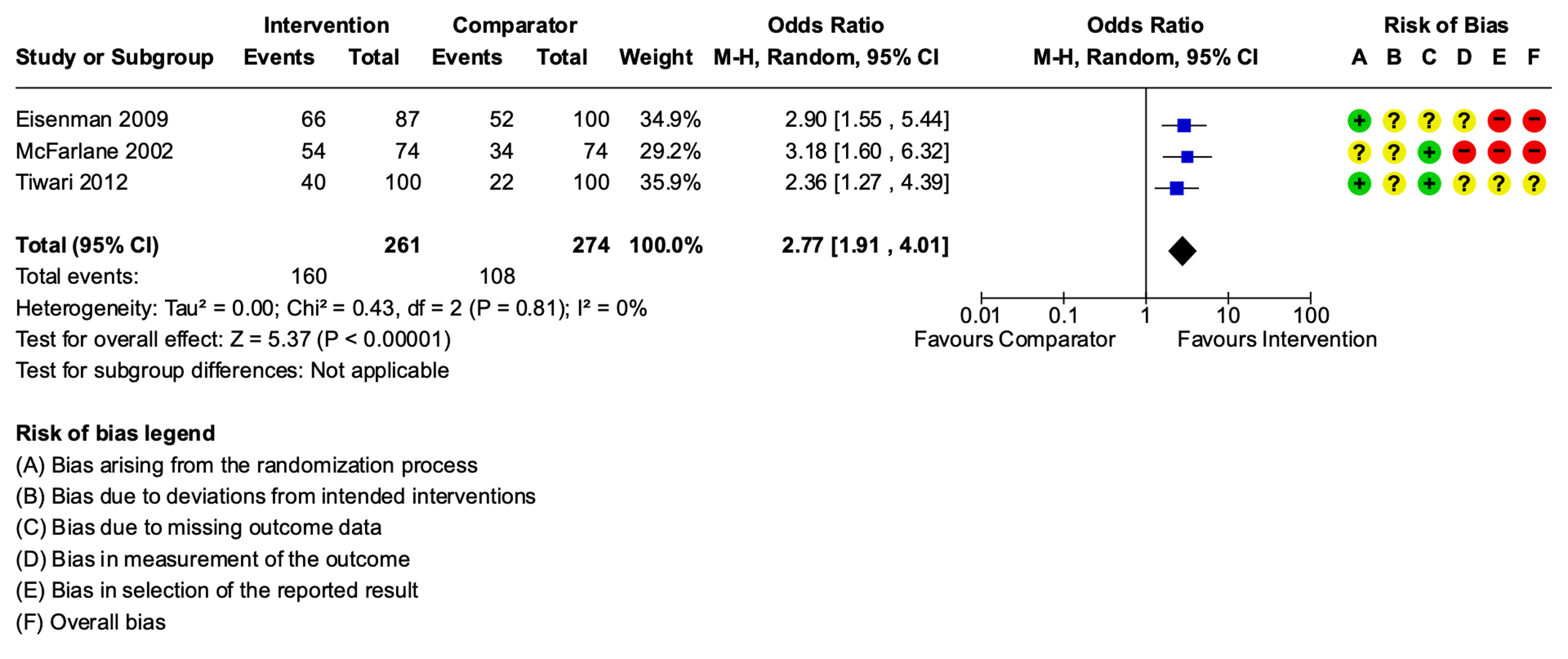


**2.14 Analysis 1.14 – intervention vs comparator – RCTs, outcome 14: recorded communication plan (last measured)**


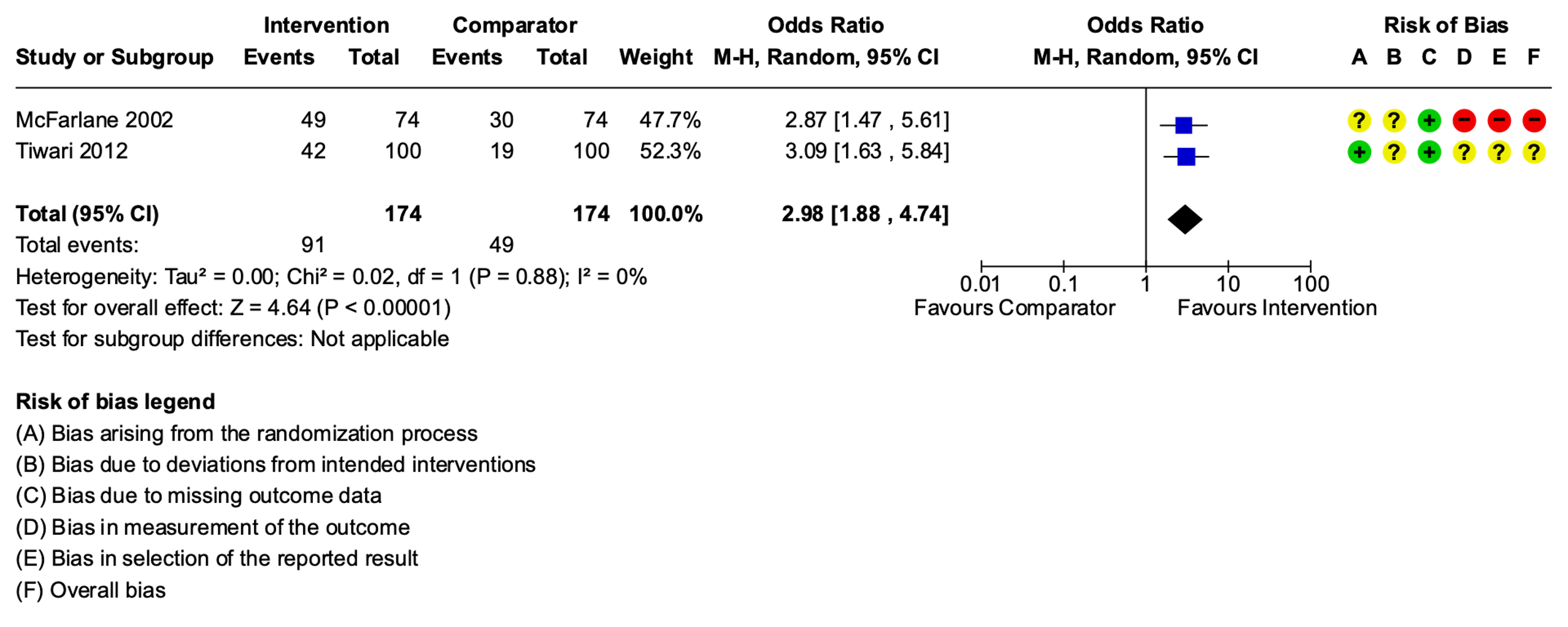


**2.15 Analysis 1.15 – intervention vs comparator – RCTs, outcome 15: stored documents**


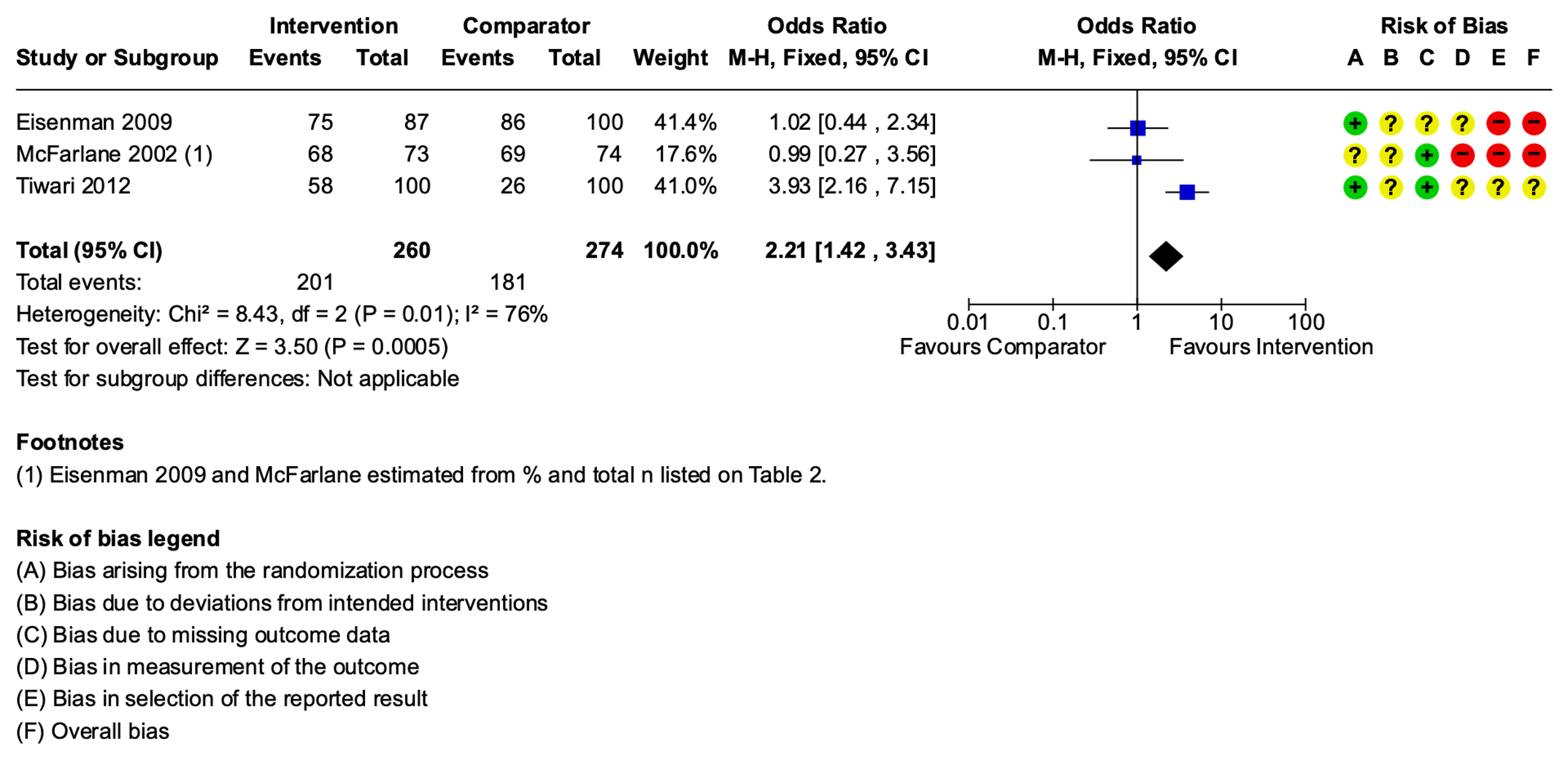


**2.16 Analysis 1.16 – intervention vs comparator – RCTs, outcome 16: stored documents (last measured)**


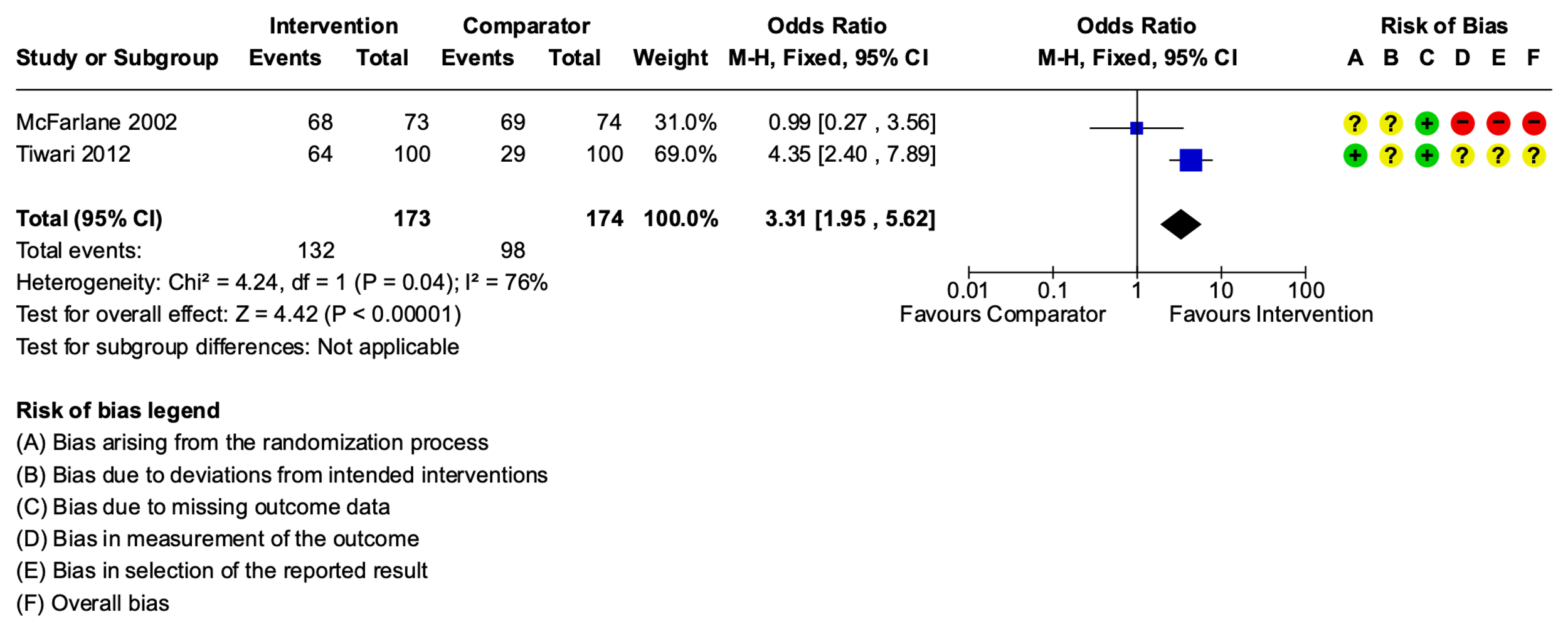


**2.17 Analysis 1.17 – intervention vs comparator – RCTs, outcome 17: recorded contact information**


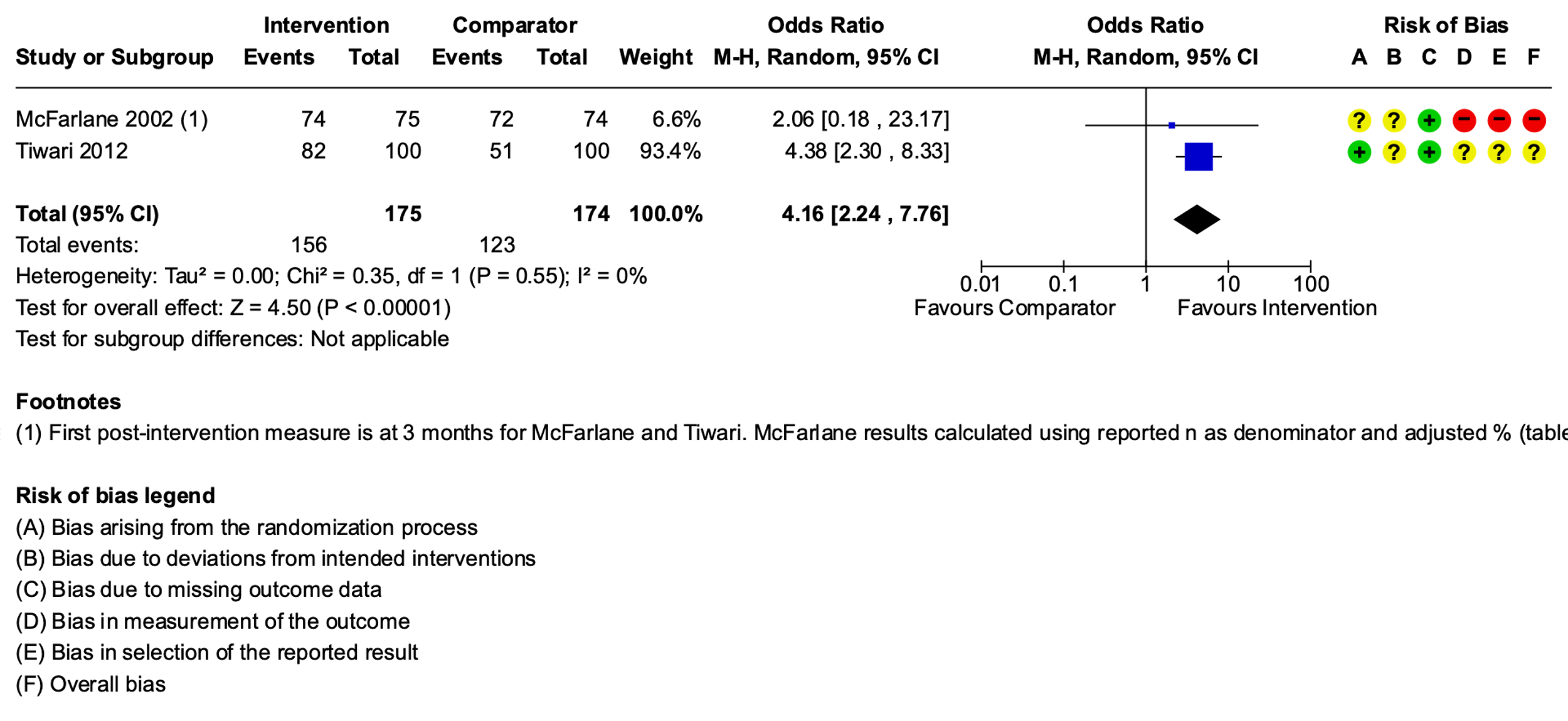


**2.18 Analysis 1.18 – intervention vs comparator – RCTs, outcome 18: recorded contact information (last measured)**


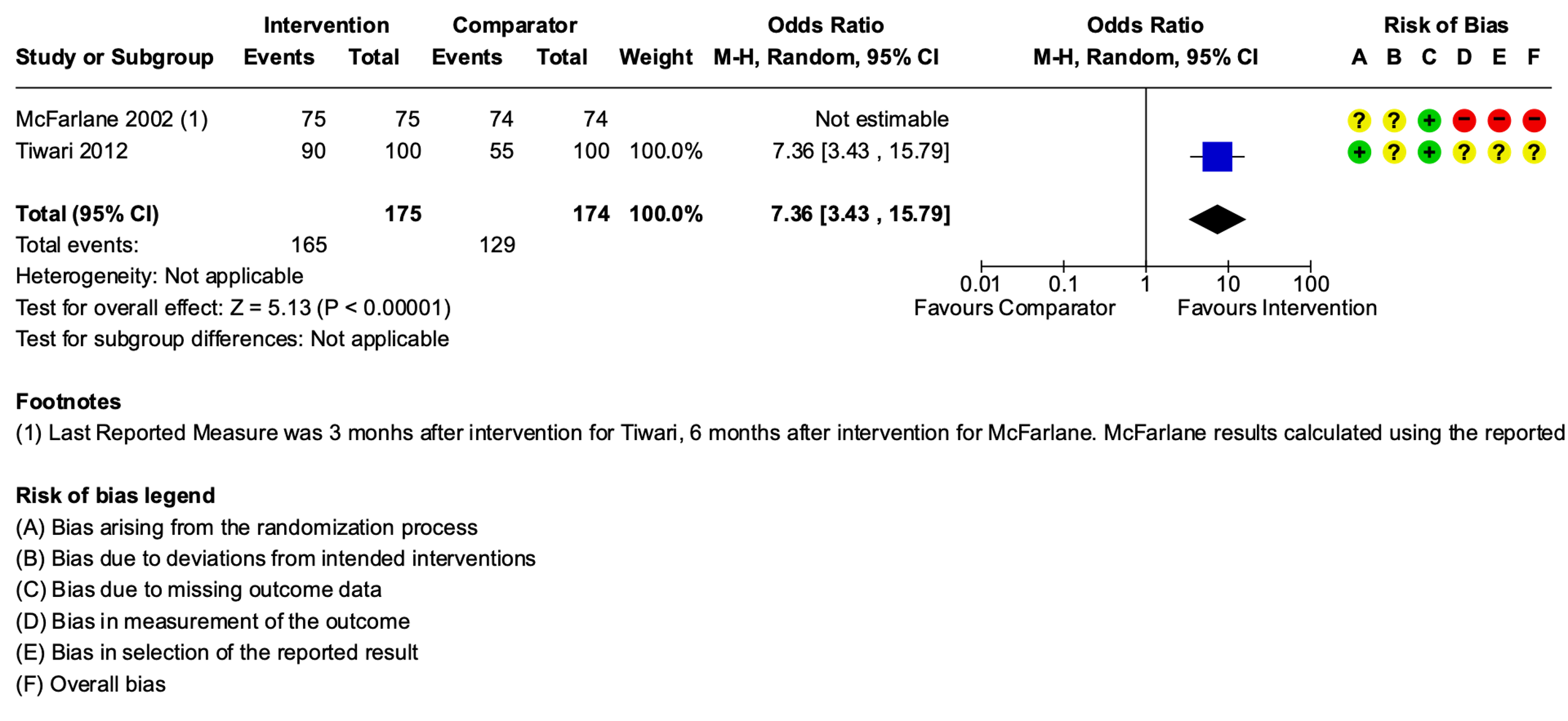


**2.19 Analysis 1.19 – intervention vs comparator – RCTs, outcome 19: ready-to-go bag**


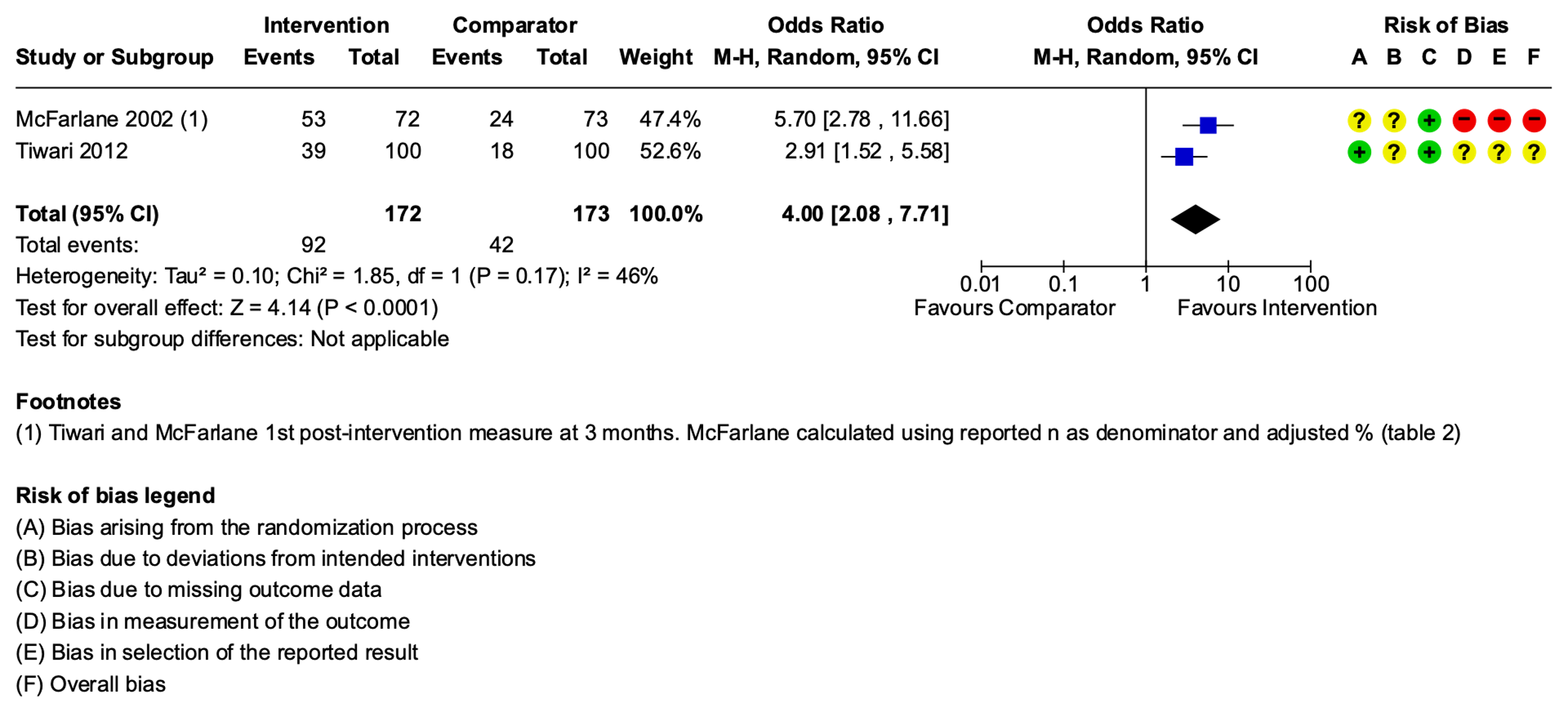


**2.20 Analysis 1.20 – intervention vs comparator – RCTs, outcome 20: ready-to-go bag (last measured)**


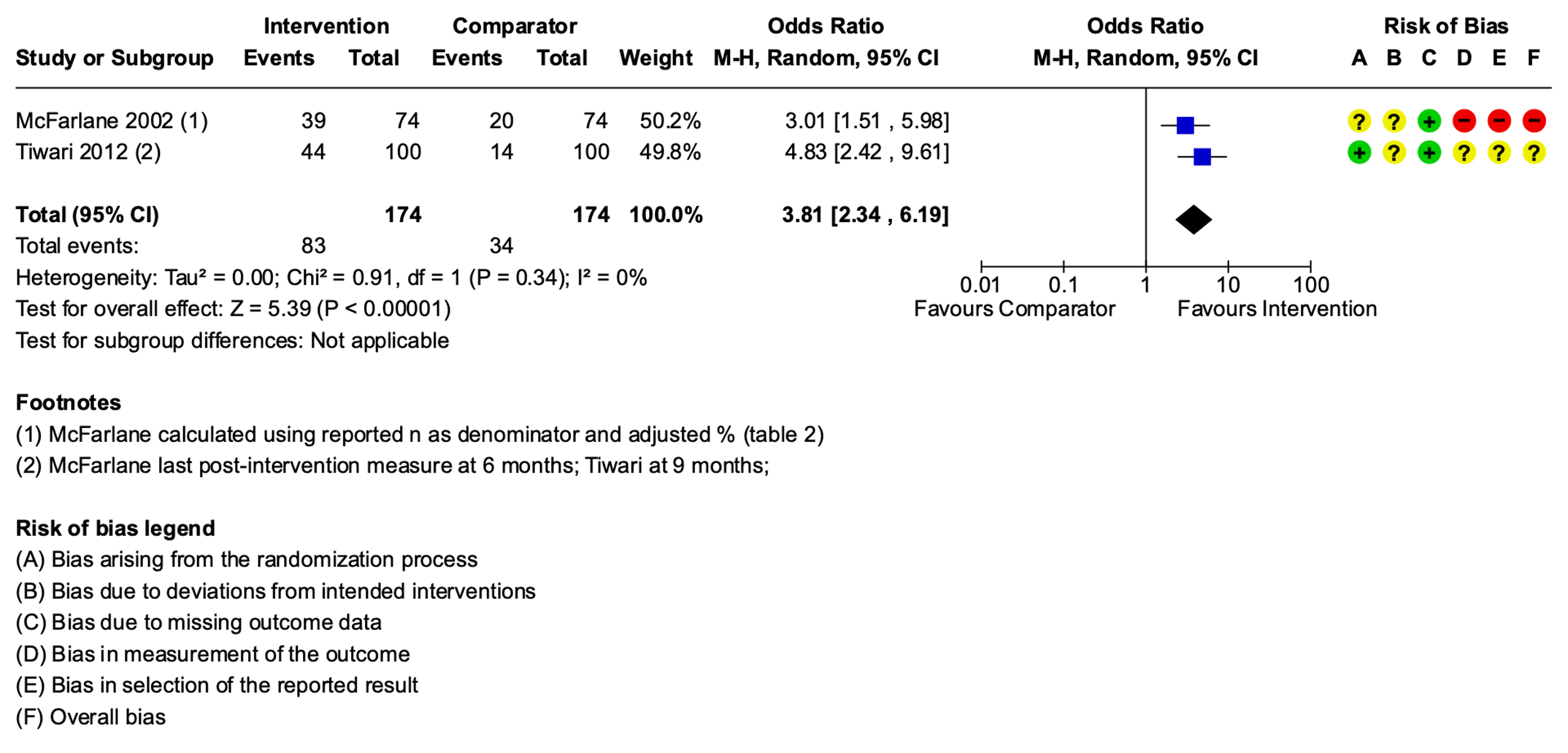


**2.21 Analysis 1.21 – intervention vs comparator – RCTs, outcome 21: knows most likely to occur types of disasters**


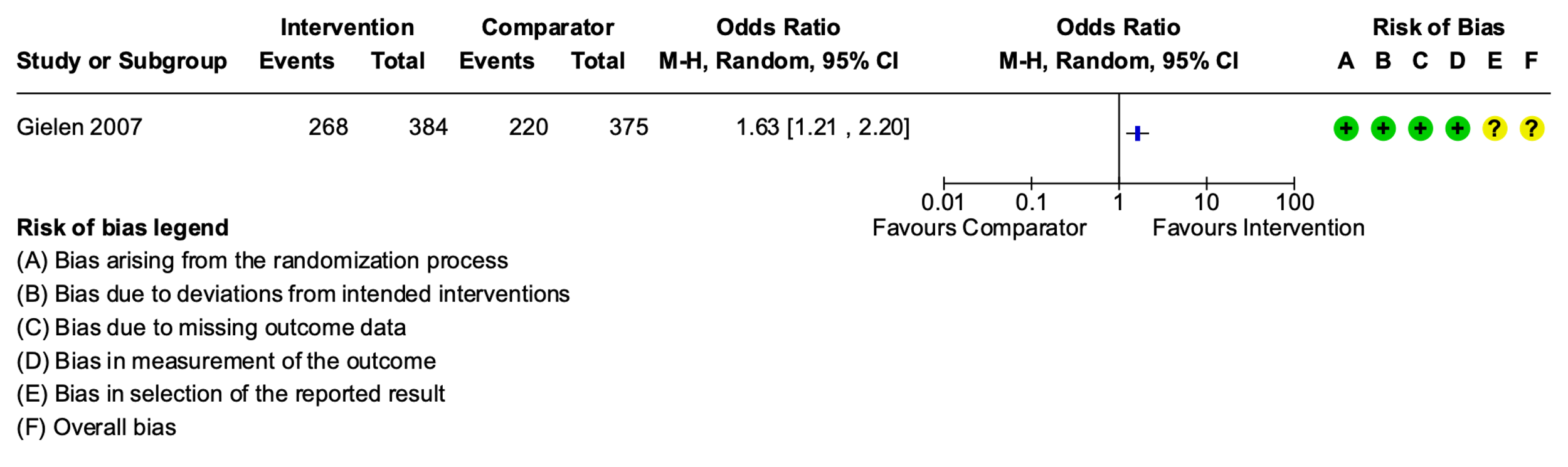


**2.22 Analysis 1.22 – intervention vs comparator – RCTs, outcome 22: mental health functioning**


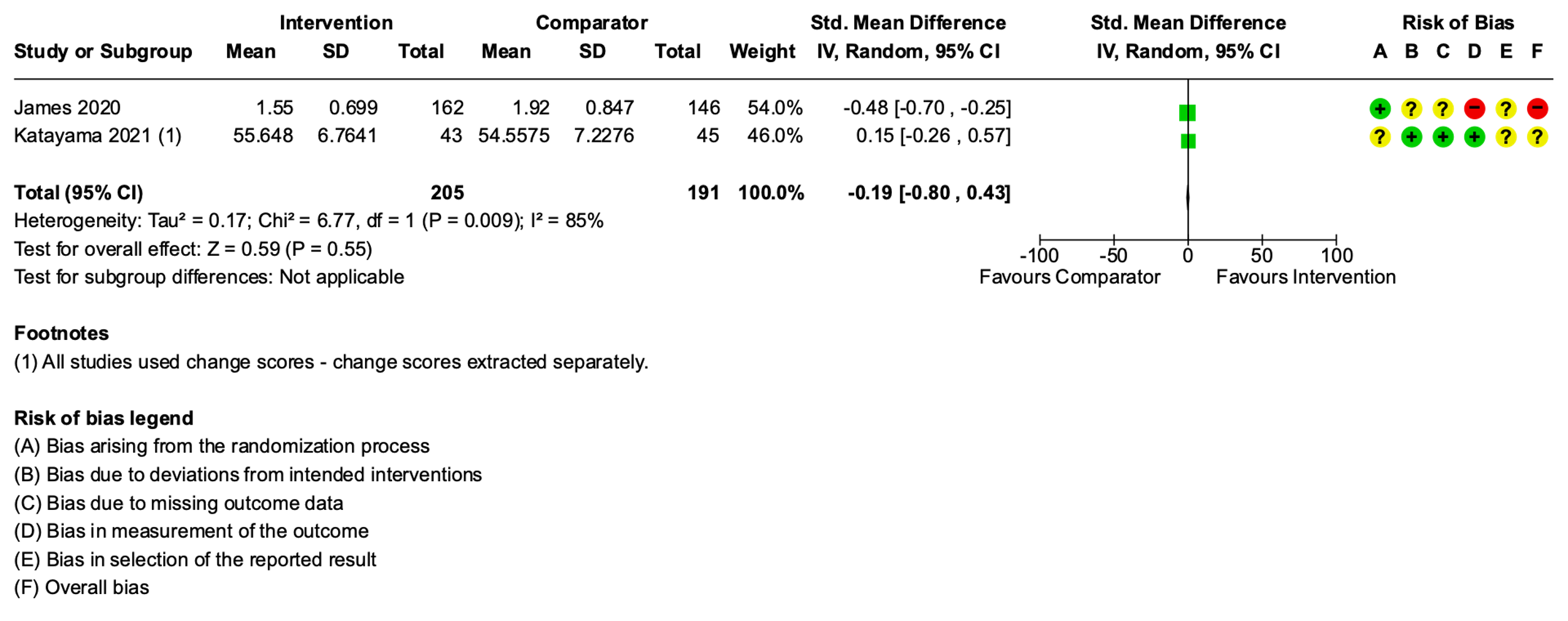


**2.23 Analysis 1.23 – intervention vs comparator – RCTs, outcome 23: physical health functioning**


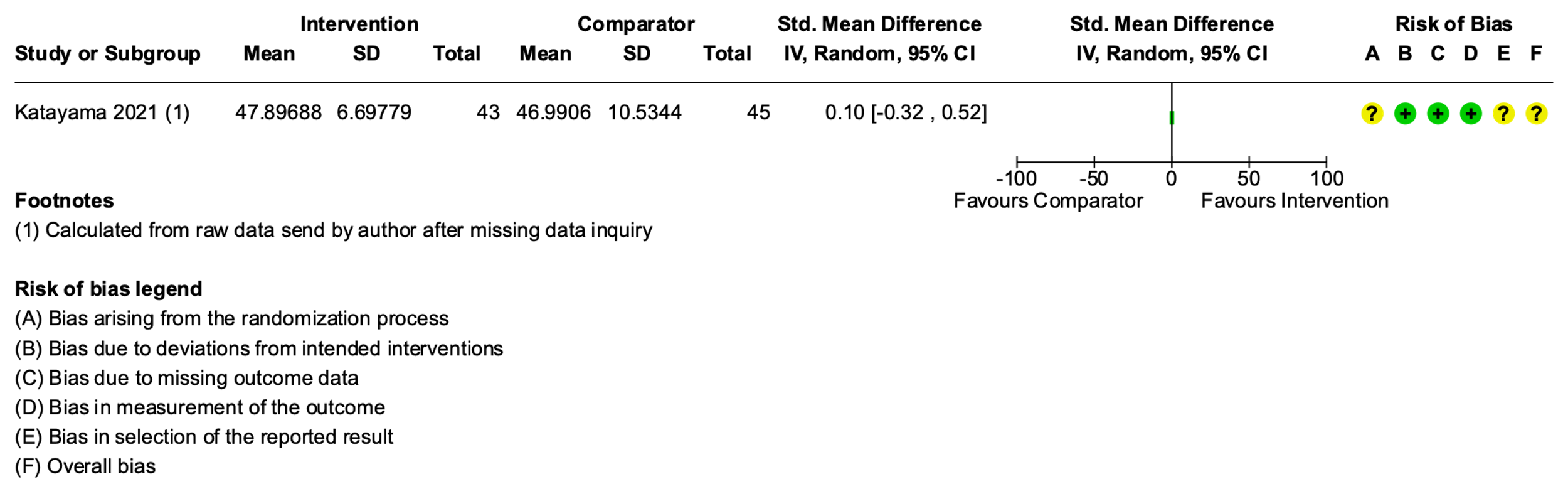


**2.24 Analysis 2.1 – intervention vs comparator – nRCTs, outcome 1: preparedness behavior**


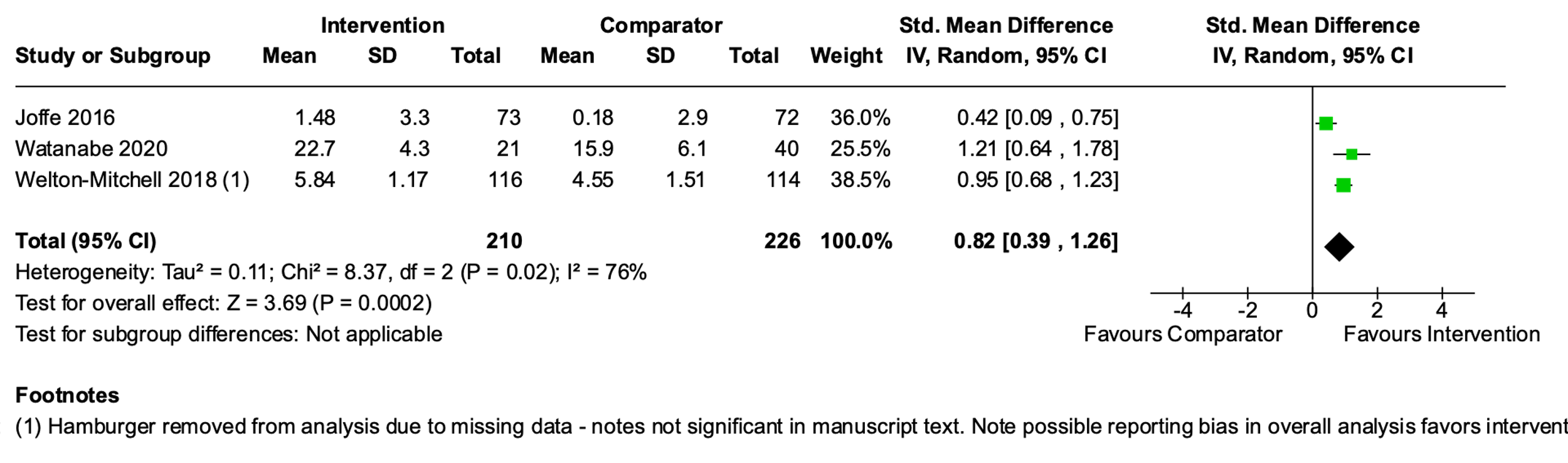


**2.25 Analysis 2.2 – intervention vs comparator – nRCTs, outcome 2: preparedness behavior (last measured)**


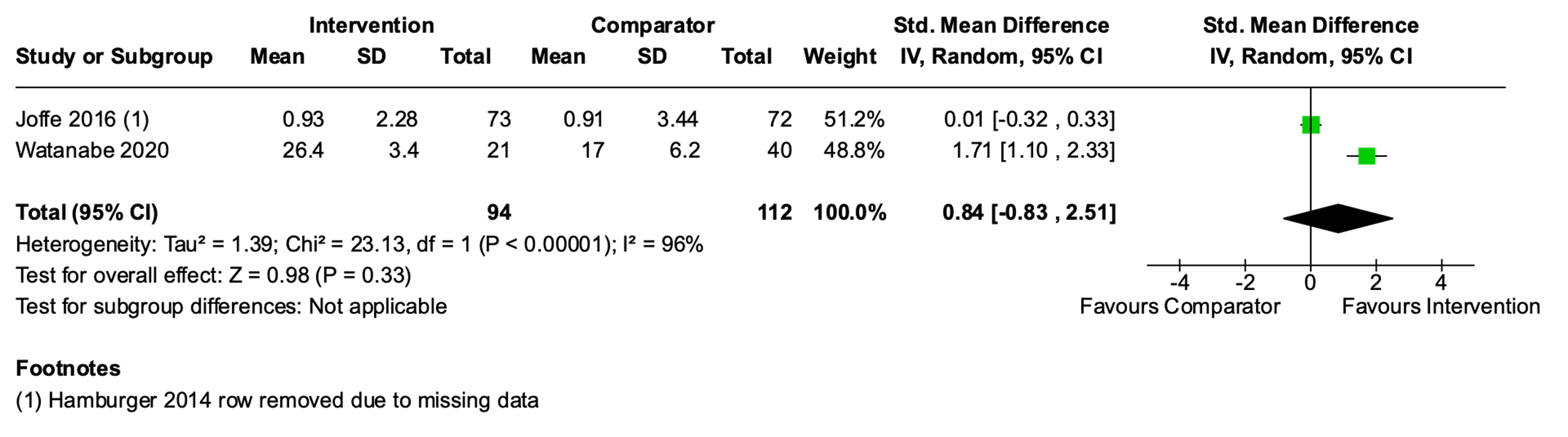


**2.26 Analysis 2.3 – intervention vs comparator – nRCTs, outcome 3: preparedness knowledge**


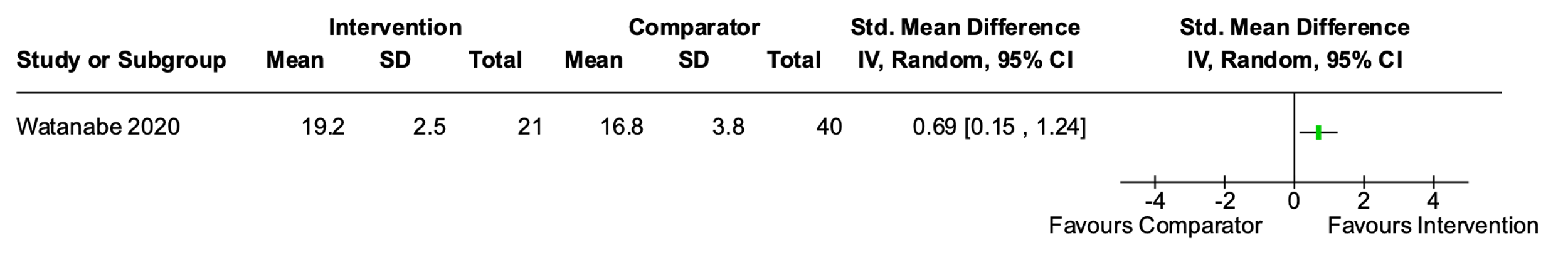


**2.27 Analysis 2.4 – intervention vs comparator – nRCTs, outcome 4: preparedness knowledge (last measured)**


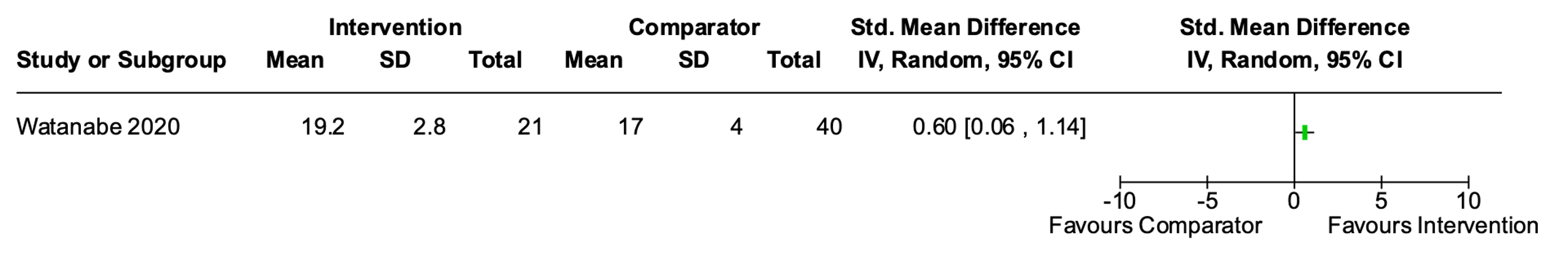


**2.28 Analysis 2.5 – intervention vs comparator – nRCTs, outcome 5: potable water**


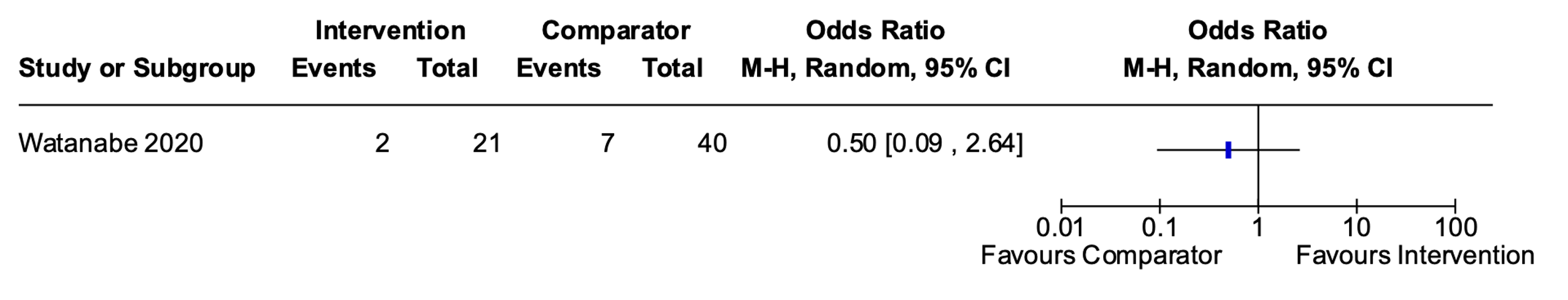


**2.29 Analysis 2.6 – intervention vs comparator – nRCTs, outcome 6: potable water (last measured)**


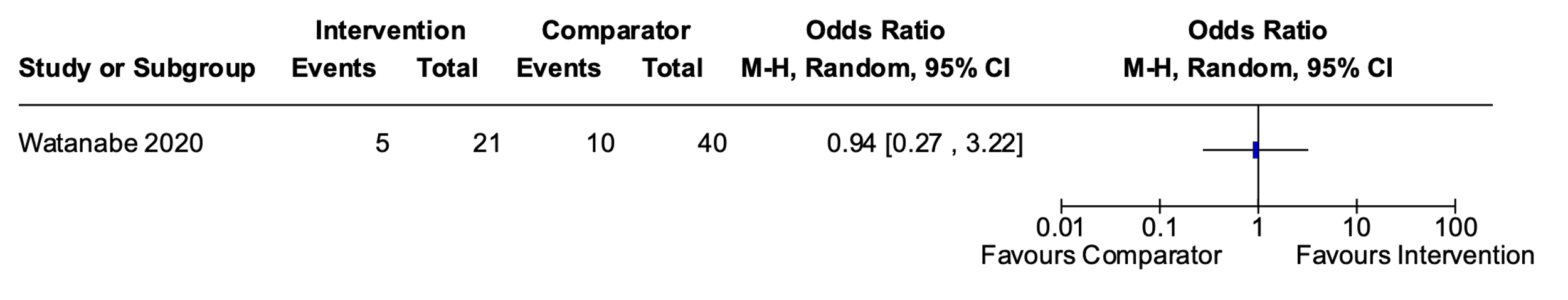


**2.30 Analysis 2.7 – intervention vs comparator – nRCTs, outcome 7: light source**


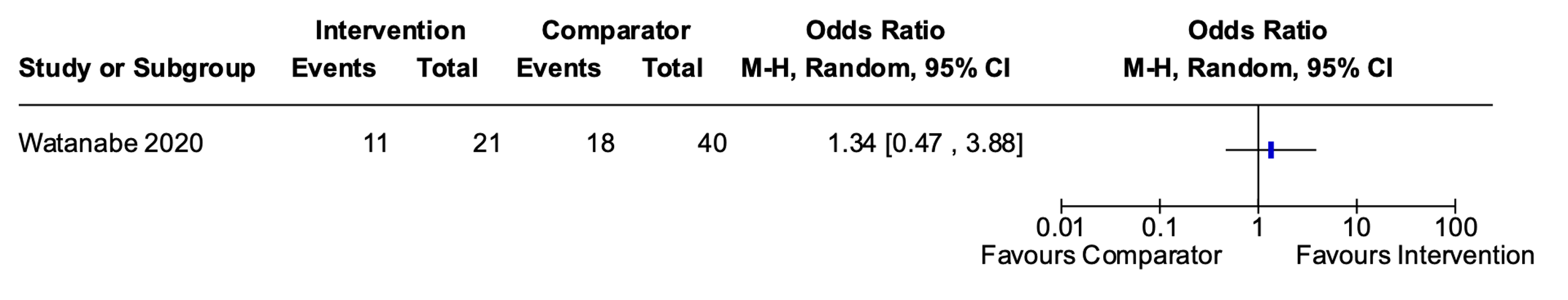


**2.31 Analysis 2.8 – intervention vs comparator – nRCTs, outcome 8: light source (last measured)**


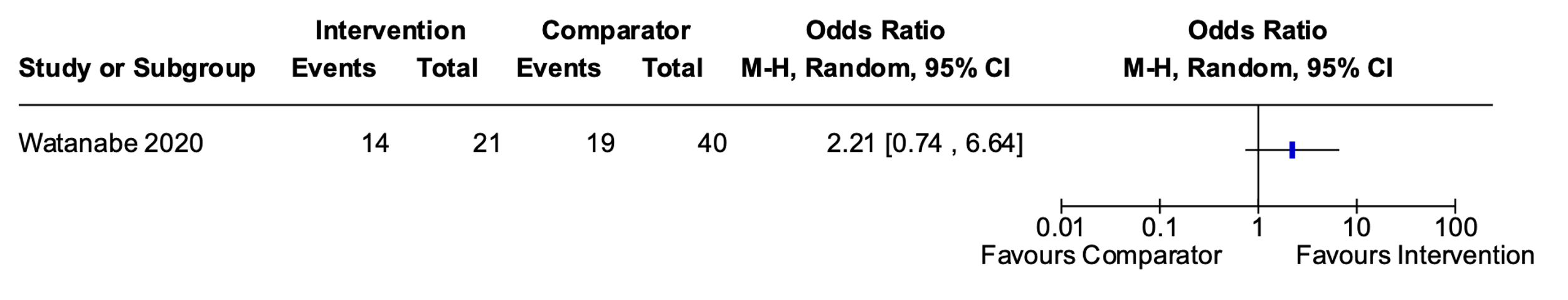


**2.32 Analysis 2.9 – intervention vs comparator – nRCTs, outcome 9: recorded evacuation plan**


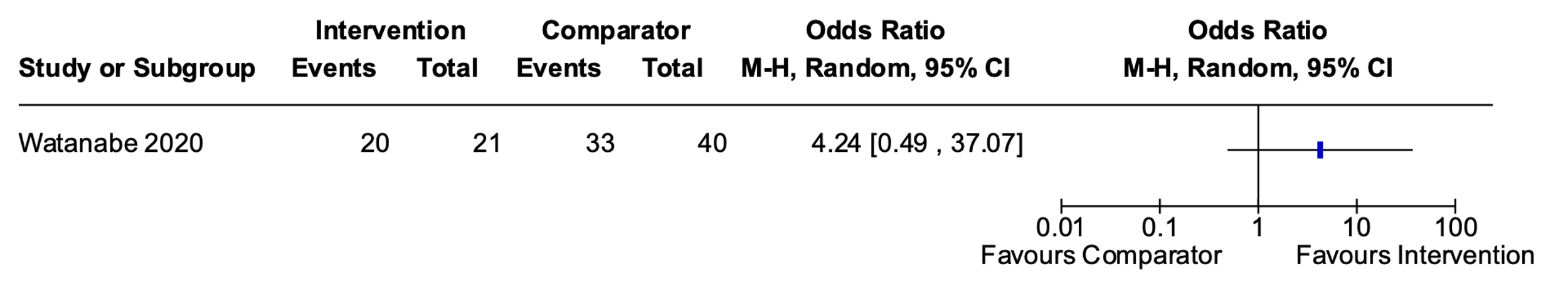


**2.33 Analysis 2.10 – intervention vs comparator – nRCTs, outcome 10: recorded evacuation plan (last measured)**


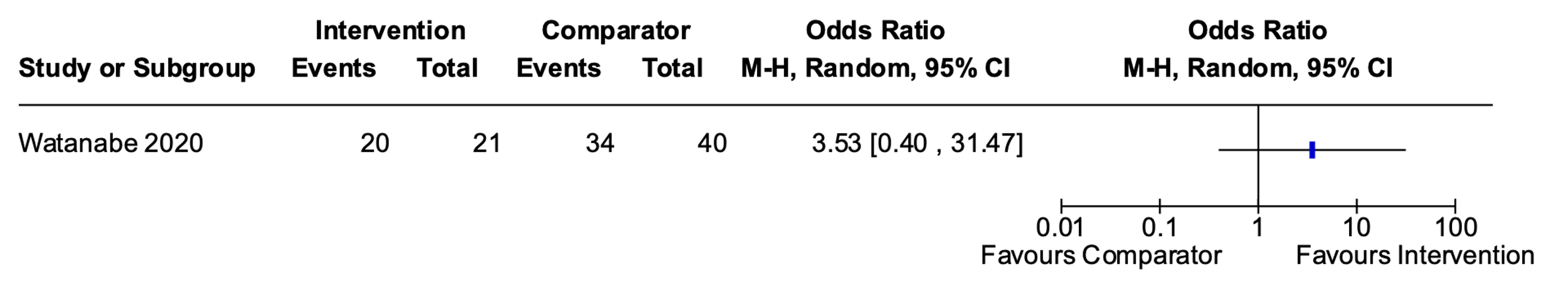


**2.34 Analysis 2.11 – intervention vs comparator – nRCTs, outcome 11: recorded communication plan**


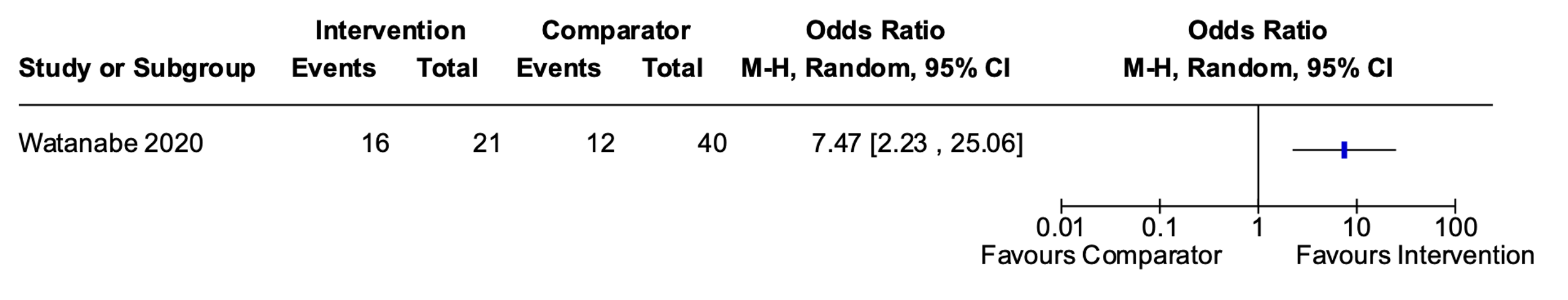


**2.35 Analysis 2.12 – intervention vs comparator – nRCTs, outcome 12: recorded communication plan (last measured)**


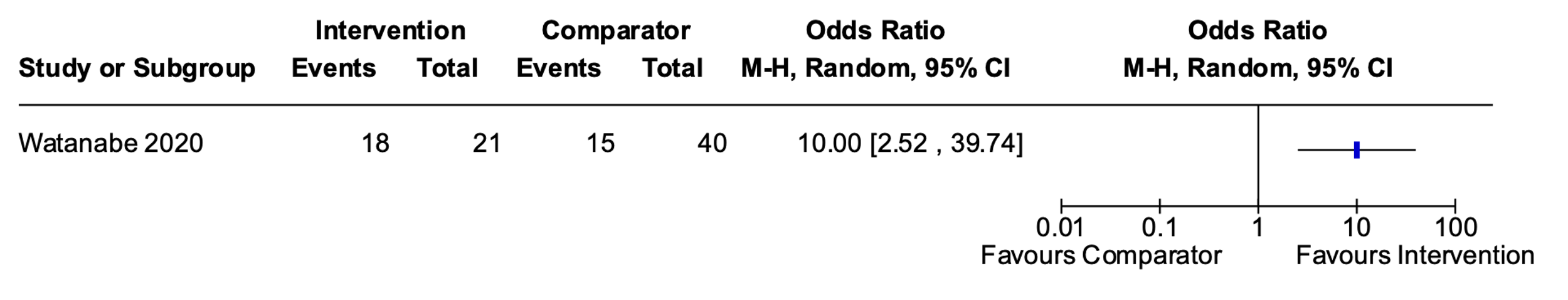


**2.36 Analysis 2.13 – intervention vs comparator – nRCTs, outcome 13: stored documents**


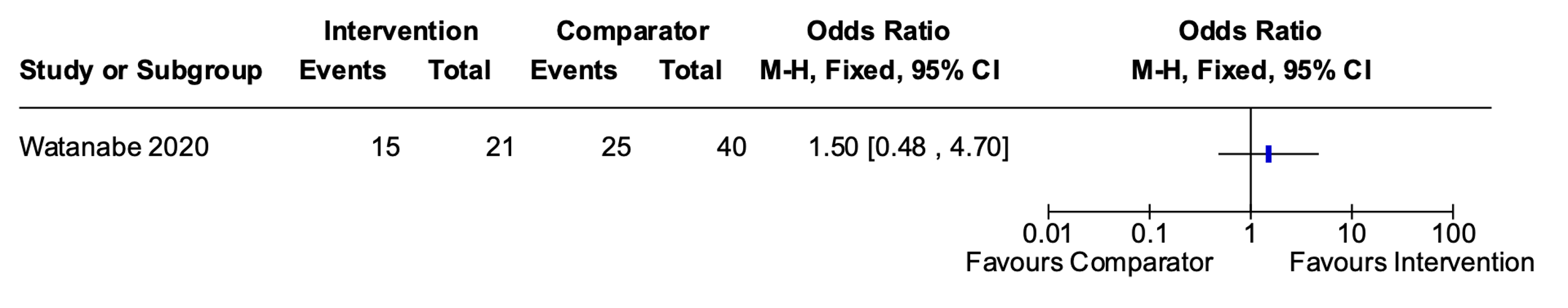


**2.37 Analysis 2.14 – intervention vs comparator – nRCTs, outcome 14: stored documents (last measured)**


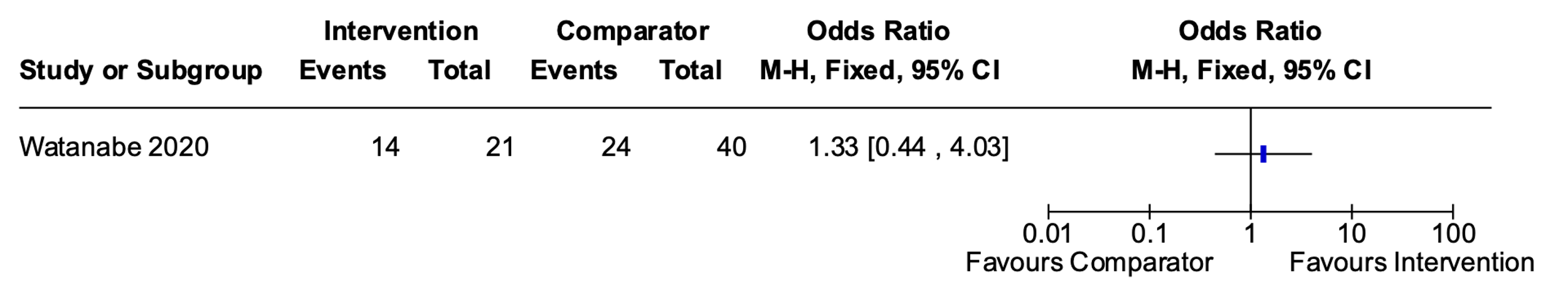


**2.38 Analysis 2.15 – intervention vs comparator – nRCTs, outcome 15: health history**


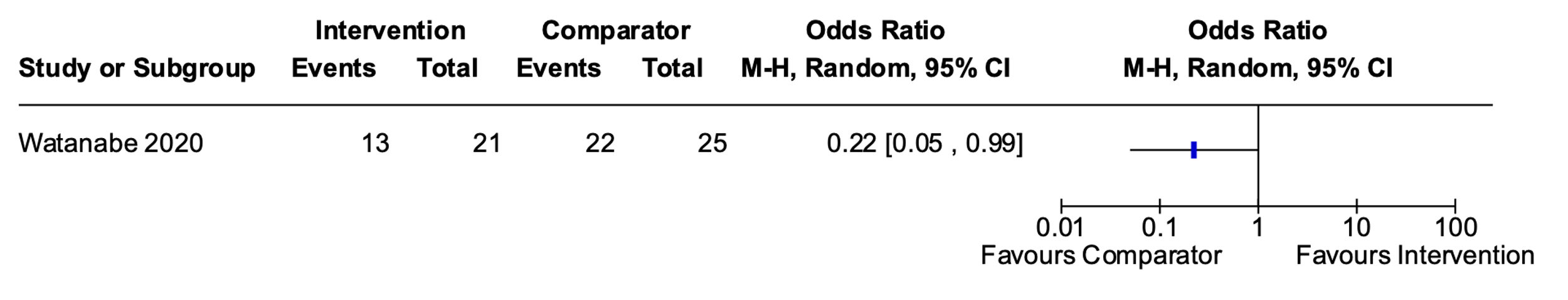


**2.39 Analysis 2.16 – intervention vs comparator – nRCTs, outcome 16: health history (last measured)**


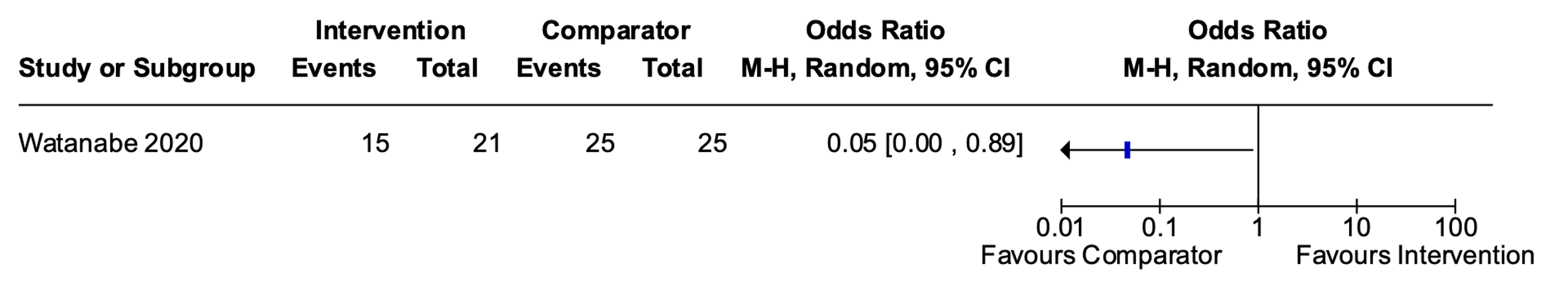


**2.40 Analysis 2.17 – intervention vs comparator – nRCTs, outcome 17: recorded contact information**


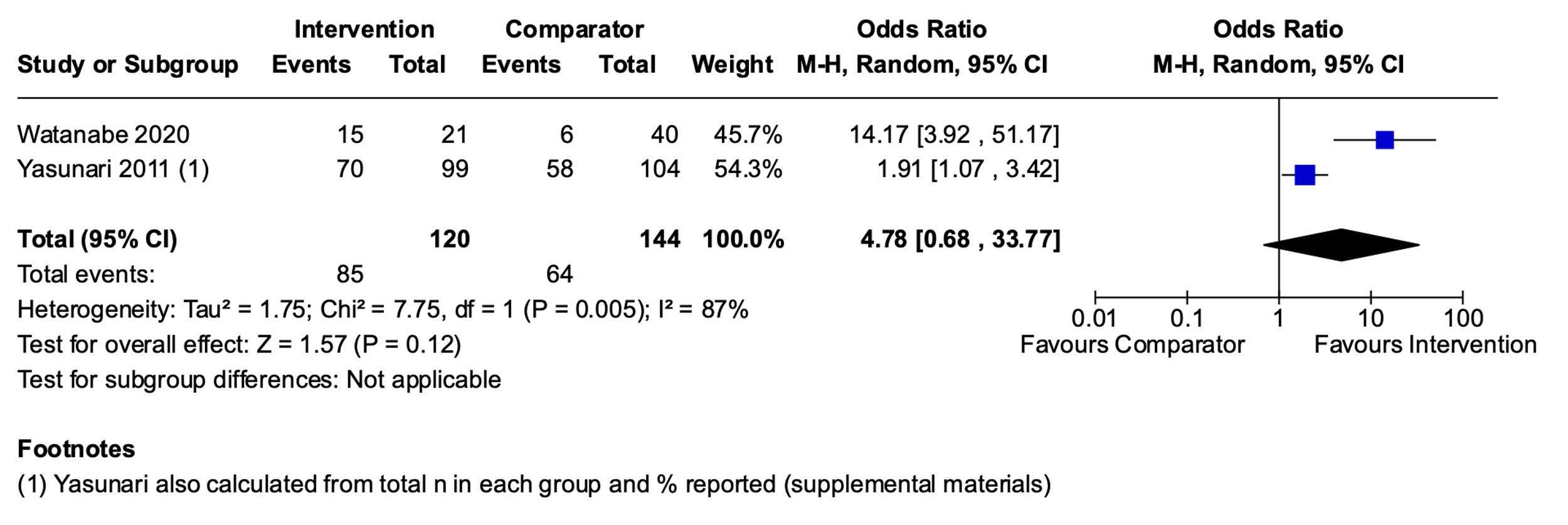


**2.41 Analysis 2.18 – intervention vs comparator – nRCTs, outcome 18: recorded contact information (last measured)**


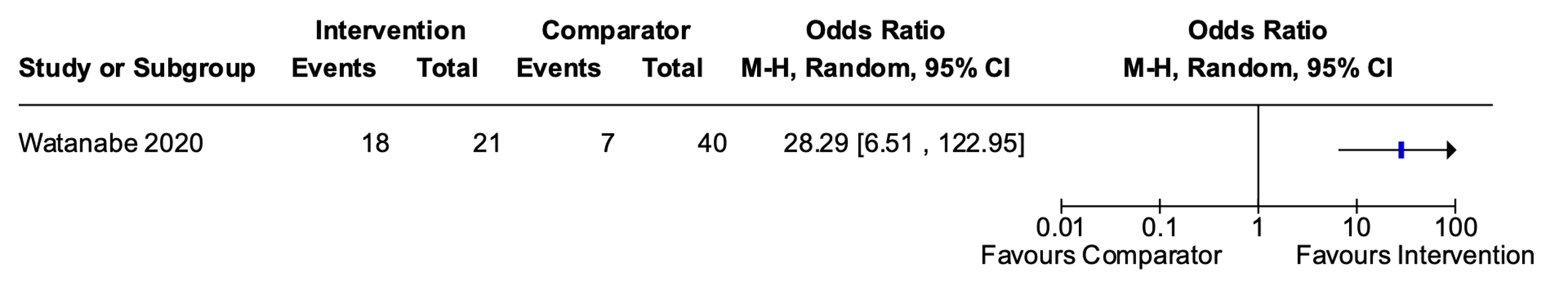


**2.42 Analysis 2.19 – intervention vs comparator – nRCTs, outcome 19: ready-to-go bag**


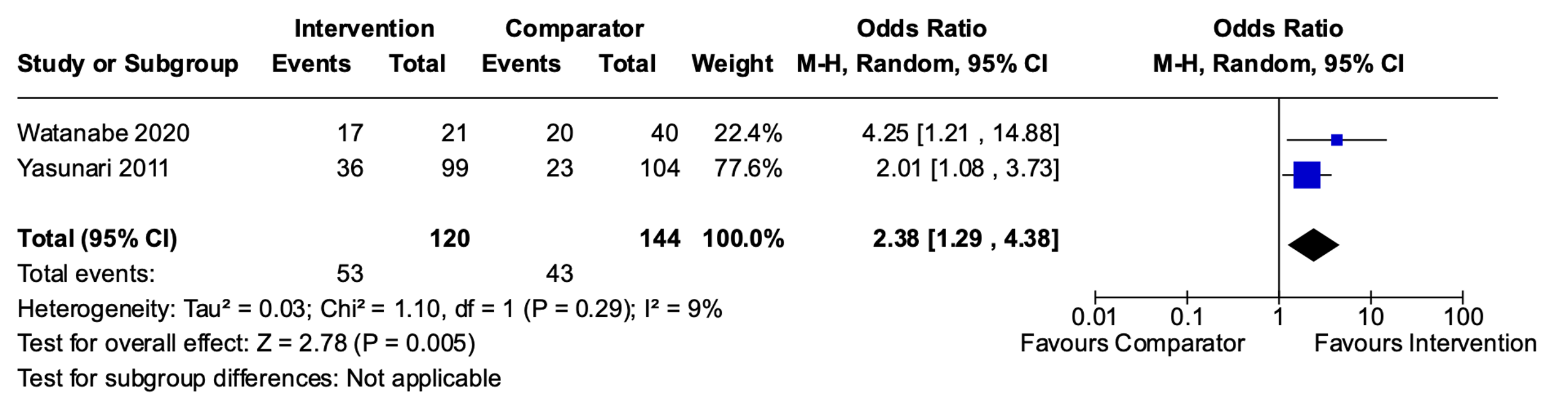


**2.43 Analysis 2.20 – intervention vs comparator – nRCTs, outcome 20: ready-to-go bag (last measured)**


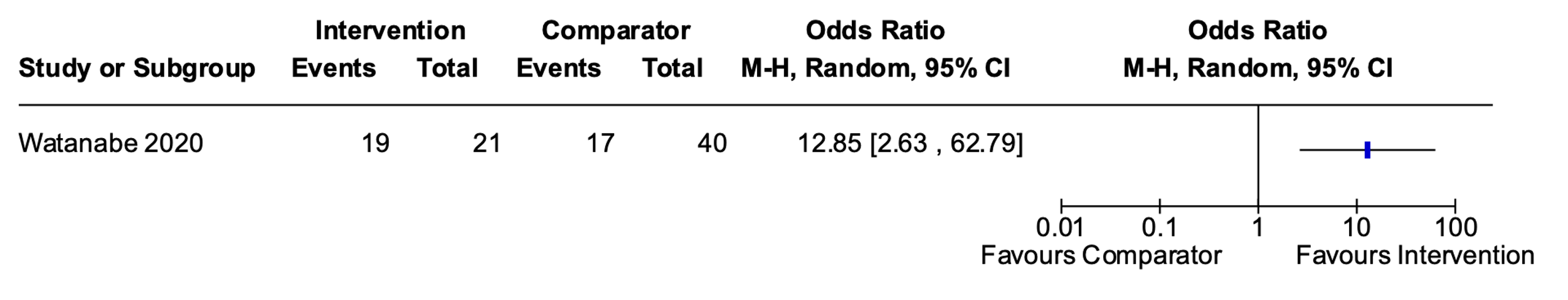


**2.44 Analysis 2.21 – intervention vs comparator – nRCTs, outcome 21: knows location of emergency shelter**


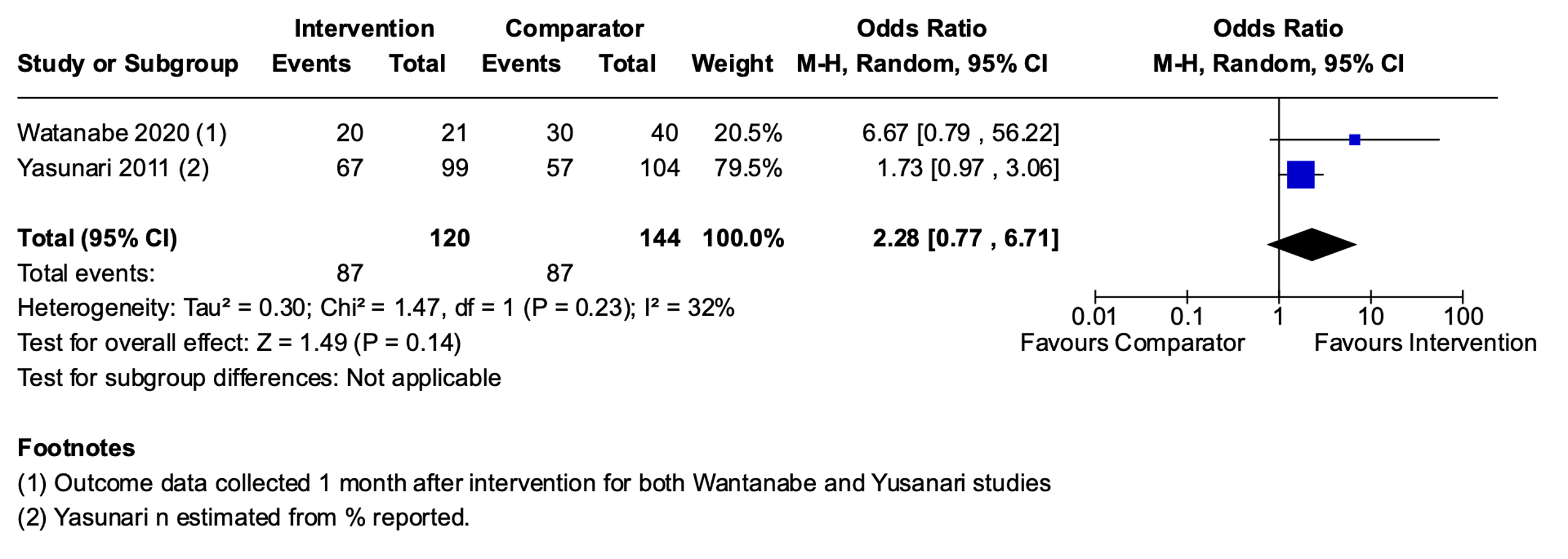


**2.45 Analysis 2.22 – intervention vs comparator – nRCTs, outcome 22: knows location of emergency shelter (last measured)**


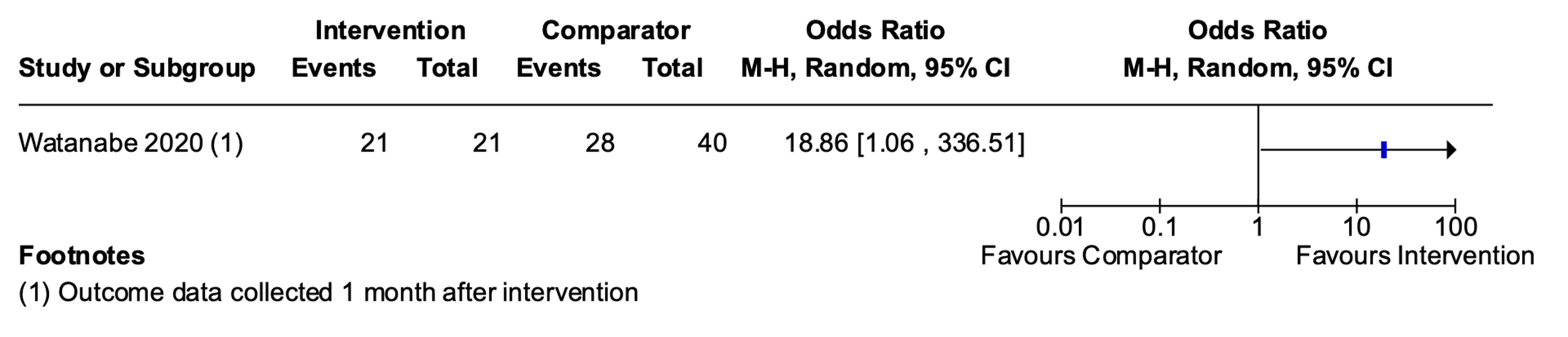


**2.46 Analysis 2.23 – intervention vs comparator – nRCTs, outcome 23: health care utilization**


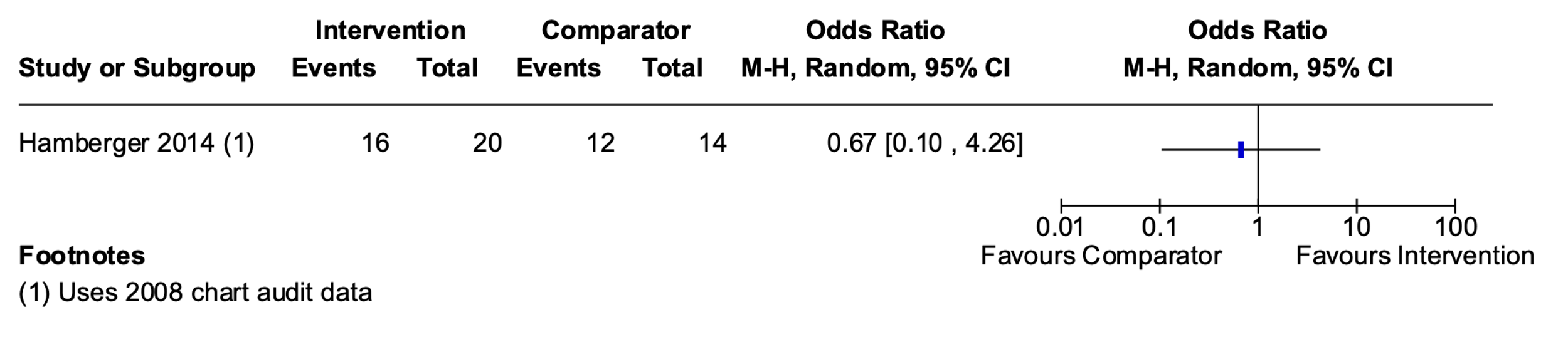


**2.47 Analysis 2.24 – intervention vs comparator – nRCTs, outcome 24: mental health functioning**


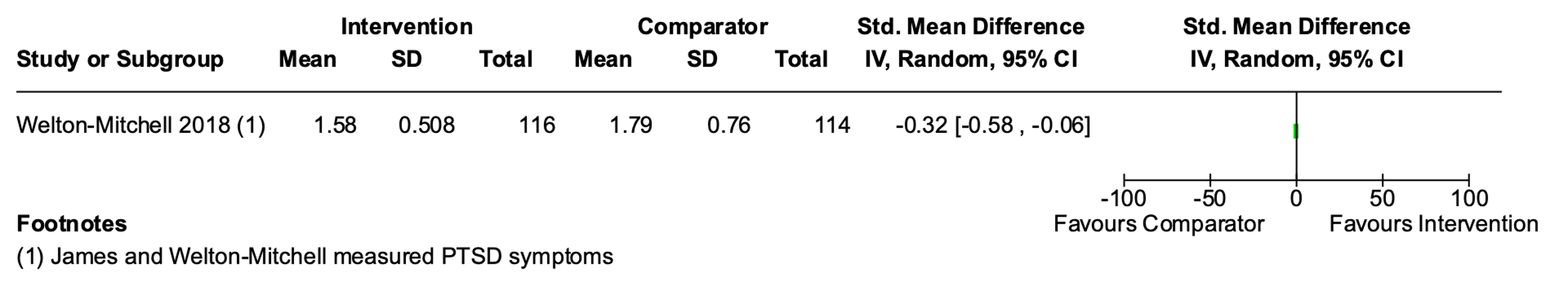

Supplement: Supplementary file 1 [file Data_Sheet_1.ZIP › Supplementary_Material.docx]
